# Solving a running crab spider puzzle: delimiting *Cleocnemis* Simon, 1886 with implications on the phylogeny and terminology of genital structures of Philodromidae

**DOI:** 10.1186/s40850-022-00136-7

**Published:** 2022-09-07

**Authors:** André Wanderley do Prado, Renner Luiz Cerqueira Baptista, Hector Baruch Pereira Schinelli, Daniela Maeda Takiya

**Affiliations:** 1 Laboratório de Diversidade de Aracnídeos, Universidade Do Brasil/Universidade Federal Do Rio de Janeiro, Av. Carlos Chagas Filho 373, 21941-902, Ilha Do Fundão, Rio de Janeiro, RJ Brazil; 2 Programa de Pós-Graduação em Biodiversidade e Biologia Evolutiva, Universidade Federal Do Rio de Janeiro, Rio de Janeiro, RJ Brazil; 3 Laboratório de Entomologia, Departamento de Zoologia, Instituto de Biologia, Universidade Federal do Rio de Janeiro, Cidade Universitária, Rio de Janeiro, RJ 21941-902 Brazil

**Keywords:** Systematics, Araneae, *Tibelloides*, Thanatinae, Philodrominae

## Abstract

**Background:**

Among the 16 Neotropical genera of Philodromidae, *Cleocnemis* has the most troublesome taxonomic situation. Remarkable morphological differences among several genera historically said to be related to *Cleocnemis* denote controversial notions and general uncertainty about the genus identity. Thus, to clarify the genus limits and contribute to the understanding of Neotropical Philodromidae, we conducted a morphological analysis, along with Bayesian Inference and Maximum Likelihood molecular phylogenetic analyses focusing on *Cleocnemis* and related genera of Thanatinae. All of the 14 species previously placed in *Cleocnemis* were studied, and eight of them included in the molecular analyses based on fragments of 28S rDNA, histone H3, 16S rDNA, and cytochrome oxidase I (COI).

**Results:**

*Cleocnemis* was recovered as polyphyletic. Most of its species are distributed into six lineages allocated into five morphologically recognizable groups: Group I [*Cleocnemis heteropoda*], representing *Cleocnemis *sensu stricto and two new junior synonyms, *Berlandiella* and *Metacleocnemis*; Group II [*Tibelloides bryantae* **comb. nov.**, *Tibelloides punctulatus* **comb. nov.**, *Tibelloides reimoseri* **nom. nov.**, and *Tibelloides taquarae* **comb. nov.**], representing *Tibelloides* **gen. rev.**, which was not recovered as monophyletic; Group III [*Fageia moschata* **comb. nov.**, *Fageia rosea* **comb. nov.**], representing the genus *Fageia*; Group IV [*“Cleocnemis” lanceolata*]; and Group V [*“Cleocnemis” mutilata*, *“Cleocnemis” serrana*, and *“Cleocnemis” xenotypa*]. Species of the latter two groups are considered *incertae sedis*. *Cleocnemis spinosa* is maintained in *Cleocnemis*, but considered a *nomen dubium*. *Cleocnemis nigra* is considered both *nomen dubium* and *incertae sedis*. We provide a redelimitation of *Cleocnemis*, redescription, neotype designation, and synonymy of type-species *C. heteropoda*. Taxonomic notes on composition, diagnosis, and distribution for each cited genus are also provided. Phylogenetic results support the division of Philodromidae into Thanatinae **new stat.** and Philodrominae **new stat.** and suggest expansion of their current compositions. Terminology of genital structures of Philodromidae is discussed.

**Conclusions:**

Our results bring light to *Cleocnemis* taxonomy and enhance the understanding of the relationships within Philodromidae, especially through the assessment of neglected Neotropical taxa.

**Supplementary Information:**

The online version contains supplementary material available at 10.1186/s40850-022-00136-7.

## Background

Philodromidae Thorell, 1870 comprises 535 species in 30 genera [1] and was originally proposed as a subfamily of Thomisidae, but raised to family level by Homann [2]. The so-called running crab spiders are agile hunters that wander usually on branches and leaves and do not use silk to catch prey. Currently, large-scale phylogenetic analyses support the monophyly of Philodromidae based on both morphological [3, 4] and molecular data [5]. Despite its well-recognized family status, relationships among its genera are poorly known, especially regarding Neotropical ones, which are often difficult to recognize by lack of proper informative descriptions and taxonomic reviews.

The first suprageneric division in Philodromidae was proposed by Schick [6], splitting the family into two tribes: Thanatini, including originally *Thanatus* C. L. Koch, 1837, *Tibellus* Simon, 1875, *Apollophanes* O. Pickard-Cambridge, 1898 and *Pelloctanes* Schick, 1965 (later synonymized with *Apollophanes* by Dondale & Redner [7]); and Philodromini, including *Ebo* Keyserling, 1884, *Titanebo* Gertsch, 1933 (as a subgenus of *Ebo*), *Philodromus* Walckenaer, 1826, and *Rhysodromus* Schick, 1965.

In the morphological phylogeny of Philodromidae of Muster [3], the preferred tree indicates that “the Thanatini sensu Schick [6] are moderately supported” (albeit represented only by *Thanatus* and *Tibellus*) and nested within “Clade I”, one of four main clades of Philodromidae, which comprises also a paraphyletic assemblage of several Philodromini species of *Rhysodromus*, *Titanebo*, and palearctic species of *Ebo*. Similarly, Thanatini (*Thanatus* + *Tibellus*) was also recovered as monophyletic with moderate support by molecular analyses of Wheeler et al. [5], on a phylogeny of Araneae including both mitochondrial (COI, 16S, and 12S) and nuclear (H3, 18S, and 28S) markers. In contrast to Muster [3], Thanatini was recovered as the sister group to a monophyletic group comprising all other five Philodromini genera sampled by Wheeler et al. [5]. The only Philodromidae phylogeny in which Thanatini did not emerge as monophyletic was the one by Griotti et al. [8] based on molecular and morphological data, with Thanatini groups placed as a polytomy at the basis of the family. By contrast, it supported an expanded composition of Philodromini including *Pulchellodromus* Wunderlich, 2012, *Pagiopalus* Simon, 1900, *Pedinopistha* Karsch, 1880 and *Petrichus* Simon, 1886.

Regarding the 16 Neotropical genera of Philodromidae, only three of them were formally revised in the last decades: *Berlandiella* Mello-Leitão, 1929 by Lise & Silva [9], *Gephyrellula* Strand, 1932 by Santos & Rheims [10] and *Petrichus* by Griotti et al. [8]. Among the remaining Neotropical genera, *Cleocnemis* Simon, 1886 has probably the most troublesome taxonomic situation. Without a modern taxonomic revision, our knowledge about the genus relies on old descriptions with few, if any, illustrations, which hampers its recognition and comparison to other genera of Philodromidae, as already marked by Pantoja et al. [11] and Dupérré [12].

*Cleocnemis* is currently represented by a diverse array of small running crab spiders, which are mainly ambush hunters on small trees, bushes, and grasses. With records in Venezuela, Guyana, Peru, Brazil, Paraguay, and Argentina, the genus is composed of 14 species of which only three have both sexes described. It was erected by Simon [13] based only on the type species, *Cleocnemis heteropoda* Simon, 1886, which was described based on an adult male and an immature female from Tijuca, Rio de Janeiro City, Brazil. Nine years later, Simon [14] mentioned that four species described by Keyserling as *Thanatus* C. L. Koch, 1837 from South America should be placed in *Cleocnemis*, but he did not name them.

A major study involving *Cleocnemis* was conducted by Mello-Leitão [15], who redescribed the type-species, described three new species from Brazil – *Cleocnemis lanceolata* Mello-Leitão, 1929, *Cleocnemis serrana* Mello-Leitão, 1929, and *Cleocnemis xenotypa* Mello-Leitão, 1929 –, and transferred three Brazilian species from other genera to *Cleocnemis*: *Cleocnemis meridionalis* (Keyserling, 1891) – later transferred to *Petrichus* Simon, 1886 by Dondale & Redner [16], *Cleocnemis mutilata* (Mello-Leitão, 1917), and *Cleocnemis taquarae* (Keyserling, 1891). In four different papers [17–20], Mello-Leitão described other five species from Brazil and Argentina: *Cleocnemis moschata* Mello-Leitão, 1943, *Cleocnemis nigra* Mello-Leitão, 1943, *Cleocnemis rosea* Mello-Leitão, 1944, *Cleocnemis rudolphi* Mello-Leitão, 1943, and *Cleocnemis spinosa* Mello-Leitão, 1947. Another species, *Cleocnemis punctulata* (Taczanowski, 1872), with records from Brazil, French Guiana, Venezuela, and Peru, was transferred to the genus by Caporiacco [21]. The two remaining species, *Cleocnemis bryantae* (Gertsch, 1933) and *Cleocnemis paraguensis* (Gertsch, 1933), were described from Paraguay (Gertsch, 1933) and transferred to *Cleocnemis* by Dondale & Redner [7].

Historically, *Cleocnemis* have been associated with members of Thanatini sensu Schick [6]. In its original description, Simon [13] stated that the genus is similar to *Thanatus* (“*Thanato* affinis”) and throughout the last years, members of *Cleocnemis* have been mentioned as similar to *Berlandiella* [9, 11] and to *Tibellus* [22]. However, some characters used to differentiate *Cleocnemis* from other genera, such as, the presence or absence of scopulae, are often contradictory among different papers (e.g. [9] and [22]).

Other Neotropical Philodromidae genera that have been connected in some way to *Cleocnemis* are *Metacleocnemis* Mello-Leitão, 1929, *Procleocnemis* Mello-Leitão, 1929 [14], and *Paracleocnemis* Schiapelli & Gerschman, 1942 [23]. *Metacleocnemis* is a monotypic genus including only *Metacleocnemis borgmeyeri* Mello-Leitão, 1929, described based on an immature specimen from Petrópolis, Rio de Janeiro State, Brazil. *Procleocnemis* includes also only its type-species, *Procleocnemis concolor* Mello-Leitão, 1929, also described based on a female from Petrópolis. *Paracleocnemis* was described to accommodate only one species, *Paracleocnemis termalis* Schiapelli & Gerschman, 1942, based on a female from Termas de Río Hondo, Santiago del Estero, Argentina. Later, a second species was described as *Paracleocnemis apostoli* Mello-Leitão, 1945, based on a male from Manantiales, Corrientes, Argentina.

The remarkable morphological differences among all these genera historically related to *Cleocnemis* denote controversial notions and general uncertainty about the genus identity. Thus, in order to redescribe *Cleocnemis* and its species, it becomes crucial to elucidate the identity of the genus through a rigorous analysis of type-materials and original descriptions. In addition, despite the putative association of *Cleocnemis* with Thanatini, a phylogenetic analysis including also Philodromini sensu Schick [6] terminals is needed to clarify its systematic position and placement of the distinct species currently allocated in the genus.

Herein we aimed to elucidate the identity, limits, and systematic position of *Cleocnemis.* We conducted a detailed analysis of available type-materials and original descriptions of all *Cleocnemis* species. Also, in order to test its monophyly and infer its phylogenetic placement, we performed phylogenetic analyses based on DNA sequences, including representatives of eight of its 14 included species, as well as, members of both Thanatini and Philodromini. Considering that phylogenies including multiple representatives of Neotropical genera of Philodromidae are scarce, our analyses represent a valuable contribution to the knowledge of the family’s systematics. Furthermore, we provide a detailed discussion on Philodromidae genitalia terminology in order to clarify the use of anatomical terms in this family.

This is the first of a series of studies on *Cleocnemis* and related genera aiming to improve the knowledge of Neotropical running crab spiders, as part of the results of the Ph. D. dissertation of the first author.

## Methods

### Material examined

Specimens studied are deposited in the following institutions (abbreviations and curators within parentheses): Instituto Butantan, São Paulo, Brazil (IBSP, A. Brescovit); Laboratório de Diversidade de Aracnídeos, Universidade Federal do Rio de Janeiro, Rio de Janeiro, Brazil (LABAR, R. Baptista); Museo Nacional de Historia Natural del Paraguay, Asunción, Paraguay (IBNP, J. Kochalka); Museu de Ciências e Tecnologia, Pontifícia Universidade Católica do Rio Grande do Sul, Porto Alegre, Brazil (MCTP, R. Teixeira); Museu Nacional, Universidade Federal do Rio de Janeiro, Rio de Janeiro, Brazil (MNRJ, A. Kury); Museu Paraense Emílio Goeldi, Belém, Brazil (MPEG, A. Bonaldo); Museum and Institute of Zoology, Polska Akademia Nauk (Polish Academy of Sciences), Warsaw, Poland (MIZ, W. Wawer); Muséum National d ´Histoire Naturelle, Paris, France (MNHN, C. Rollard); Museum of Comparative Zoology, Harvard University, Cambridge, USA (MCZ, G. Giribet); Natural History Museum, London, England (NHMUK, J. Beccaloni); Naturhistorisches Museum Basel, Basel, Switzerland (NHMB, A. Hänggi); and Universidade Federal de Minas Gerais, Belo Horizonte, Brazil (UFMG, A. Santos). Type-material of seven out of 14 species of *Cleocnemis* were examined through photographs or loans, namely of *C. bryantae* (Gertsch, 1933), *C. lanceolata* Mello-Leitão, 1929, *C. paraguensis* (Gertsch, 1933), *C. punctulata* (Taczanowski, 1872), *C. rosea* Mello-Leitão, 1944, *C. rudolphi* Mello-Leitão, 1943, and *C. taquarae* (Keyserling, 1891). Types of the other seven species are considered lost. Information about type-materials of each analyzed species is given in taxonomy section, but that of additional material examined is provided in Additional file 1 and was standardized with Automatex [24].

### Terminology and abbreviations

Identification of the material was carried out by comparison with type-material and consulting original species descriptions and other taxonomic works. Descriptions and terminology were adapted from current taxonomic papers dealing with Philodromidae and other spider families (e.g., [3, 6, 9, 16, 25, 26]). A discussion on nomenclature and definitions of genitalic characters is given in the section “Notes on genitalic morphology of Philodromidae” below. Anatomical abbreviations used were as follows: (AGP) anterior guide pockets, (ALE) anterior lateral eyes, (AME) anterior median eyes, (AT) atrium, (BA) bilateral atria, (BS) base of spermatheca (receptacula sensu Muster, 2009a [3]), (C) conductor, (CA) copulatory atria, (CD) copulatory ducts, (CG) copulatory guides (guide pocket sensu Schick, 1965 [6]), (CO) copulatory openings (intromittent orifices sensu Schick, 1965 [6] and Muster, 2009a [3]), (CoP) conductor process, cymbial process (CP), dorsal tibial apophysis (DTA), (E) embolus, (EB) embolic base, **(**EP) epigynal plates, (FD) fertilization ducts, (GH) glandular head of spermatheca (spermathecal organ sensu Schick, 1965 [6] and Dondale & Redner, 1976 [7]), (GP) guide pockets (lateral guide pocket sensu Schick, 1965 [6]), (GS) glandular head stalk (spermathecal organ sensu Schick, 1965 [6]), (LP) lateral plates, (MA) mesal atrium (simply atrium sensu Schick, 1965 [6]), (MD) mesal depression, (MF) membranous field, (MS) median septum, (MOQ) median ocular quadrangle, (PCA) paraconductor bulbar apophysis, (PLE) posterior lateral eyes, (PME) posterior median eyes, (PR) posterior rim, (RMC) retrolateral marginal conductor, (RTA) retrolateral tibial apophysis, (S) spermathecae, (TC) tegular conductor, (TS) tegular suture, (VBA) ventral bulbar apophysis, and (VTA) ventral tibial apophysis. All measurements were given in millimeters. Carapace length was measured from the anterior margin of clypeus to the posterior border. Total length was measured from the anterior margin of the clypeus to the posterior border of abdomen, including the spinnerets.

### Images and material preparation

Images and descriptions of external morphology and genitalia were obtained from specimens preserved in 75% ethanol under a LEICA M205 C binocular stereoscopic microscope with a Leica DFC 450 digital camera attached. Female genitalia were dissected and clarified using a borax solution following Álvarez-Padilla & Hormiga [27] and digestive enzyme tablets “Orthoplex D.E.F.” (Bioconcepts Pty Ltd) consisting of Pancreatin (200 mg), Bromelain (100 mg), and Trypsin (30 mg), in order to remove soft tissues.

For scanning electron microscopy (SEM), specimens were cleaned ultrasonically, critical-point dried at Laboratório de Ultraestrutura Celular Hertha Meyer (LUCHM – UFRJ), mounted and coated with gold–palladium for observation. Images were obtained with a Jeol JSM 6510 microscope at Laboratório de Microscopia (Labim), Instituto de Biologia, UFRJ. Figures were edited in the software Adobe Photoshop CS6 and plates designed with Adobe Illustrator 24.1.1.

### DNA extraction, amplification, and sequencing

Genomic DNA was extracted from leg muscles of 14 specimens with the DNeasy blood & tissue kit (Qiagen, Hilden, Germany). Four molecular markers were used for phylogeny: 28S rDNA, histone H3, 16S rDNA, and cytochrome oxidase I (COI). These markers were chosen as targets due to their use in a recent publication on the phylogeny of Araneae [5].

Amplification was conducted through PCR. Most PCR reactions had a total volume of 25 μl, consisting of 12.5 –13.5 μl of H2O, 5.0 μl of PCR buffer GoTaq® Flexi (Promega, Madison, USA), 3 μl of MgCl2 (25 mM, Promega), 0.5 μl of dNTP mix (20 mM, Promega), 1 μl of each forward and reverse primer (10 pmol/l), 0.1 μl of GoTaq® Flexi Polymerase (5U/μl, Promega), and 1–2 μl of DNA extract.

Fragments of COI were amplified using the following thermocycling settings: 94 °C for 3 min, followed by 35 cycles at 94 °C for 1 min, 48 °C for 1 min, and 72 °C for 2 min, and a final extension at 72 °C for 7 min. Fragments of H3, 16S, and 28S were amplified as above, but with an annealing temperature of 50 °C. Amplified products were stained with GelRed® (Biotium, Fremont, USA) and observed under UV light after 1% agarose gel electrophoresis and compared with a molecular weight standard. Successful amplicons were purified with ExoSAP-IT™ (Applied Biosystems, Waltham, EUA) and sent to Macrogen® (South Korea) for Sanger sequencing. Primers used for PCR and sequencing are listed in Additional file 2. Complementary electropherograms were assembled and edited in GeneStudio v2.2.0. Consensus sequences generated were checked by comparison with similar sequences in GenBank® using BLAST® [28] to verify correct homology or taxonomic contamination. Sequences generated herein were deposited in GenBank® under accessions OM773126-OM773137, OM936911-OM936924, OM913603-OM913615, and OM902669-OM902673.

### Taxon sampling for phylogenetic analyses

Phylogenetic analyses were based on DNA sequences of 31 terminals (14 newly sequenced herein), comprising eight species of the 14 species placed in *Cleocnemis* before the taxonomic decisions stated in the present study. Other 17 taxa of Philodromidae were included, representing the following genera: *Apollophanes*, *Fageia* Mello-Leitão, 1929, *Gephyrellula*, *Pedinopistha*, *Philodromus*, *Thanatus*, *Tibellus*, *Pagiopalus*, *Petrichus*, and *Titanebo*. Representatives of closely related families, such as Miturgidae and Cheiracanthiidae (including genera of both Cheiracanthiinae and Eutichurinae) were also sampled, as the latter family (cited as Eutichuridae) was considered the sister group of Philodromidae by Wheeler et al. [5]. A list with species included in our phylogenetic analysis and DNA voucher specimen information is given in Table 1.

**Table 1:** Species included as terminal taxa for the phylogeny of Philodromidae. Species marked with a “*” were placed in *Cleocnemis* previous to this work. Specimen voucher codes, collecting locality (country and state), and GenBank accession codes for each of the markers used are given. Sequences generated herein are marked in bold face

Species	Voucher code	Locality	COI	H3	16S	28S
**Cheiracanthiidae**						
*Cheiracanthium inclusum* (Hentz, 1847)	ENT5214	Brazil (Rio de Janeiro)	**OM773133**	**OM936911**	**OM913615**	**OM902673**
*Cheiracanthium mildei* L. Koch, 1864	ARAMR000018	USA (New York)	KY017714^2^	KY018224^2^	KY015868^2^	KY017080^2^
Eutichuridae sp.	ARAMR000090	Madagascar (Fianarantsoa)	KY017718^2^	KY018228^2^	KY015871^2^	KY017084^2^
*Eutichurus ravidus* Simon, 1897	ARAMR000014	Argentina (Misiones)	KY017719^2^	-	KY015873^2^	KY017086^2^
**Miturgidae**						
*Miturga lineata* Simon, 1897	ARASP000098	Australia (Queensland)	KY017796^2^	-	KY015969^2^	KY017199^2^
*Teminius insularis* (Lucas, 1857)	ARAMR000032	Argentina (Entre Ríos)	KY017799^2^	-	KY015974^2^	KY017204^2^
**Philodromidae**						
*Apollophanes* sp.	9,031,470	USA (California)	KM225093^1^	KM225195^1^	-	KM225039^1^
*Cleocnemis heteropoda Simon, 1886	ENT5104	Brazil (Rio de Janeiro)	**OM773132**	**OM936912**	**OM913613**	**OM902669**
*Cleocnemis zabele* **comb. nov**	ENT5394	Paraguay (Canindeyú)	**OM773134**	**OM936914**	**OM913612**	**OM902670**
*Cleocnemis* sp.1	ENT4999	Brazil (Pernambuco)	**OM773130**	**OM936913**	**OM913611**	-
* “Cleocnemis” *lanceolata*	ENT5398	Paraguay (Canindeyú)	**OM773136**	**OM936916**	**OM913610**	-
* “Cleocnemis” mutilata	ENT4993	Brazil (Rio de Janeiro)	**OM773126**	**OM936917**	**OM913603**	-
*Fageia moschata **comb. nov**	ENT5396	Paraguay (Paraguari)	**OM773135**	**OM936915**	**OM913614**	-
*Gephyrellula violacea* (Mello-Leitão, 1918)	ENT5105	Brazil (Rio de Janeiro)	-	**OM936918**	-	-
*Pagiopalus nigriventris* Simon, 1900	USNM	USA (Maui, HI)	EU168155^2^	EU157106^2^	EU168142^2^	-
*Pedinopistha stigmatica* (Simon, 1900)	USNM	EUA (Hawaii, HI)	EU168156^2^	EU157107^2^	EU168144^2^	-
*Petrichus* sp.	ARAMR000696	Argentina (San Juan)	-	KY018348^2^	KY016028^2^	KY017260^2^
*Philodromus aureolus* (Clerck, 1758)	LEGO_42_1	Not informed	JN817234^3^	-	JN816600^3^	JN817021^3^
*Philodromus spinitarsis* Simon, 1895	LEGO_42_8	Not informed	JN817238^3^	-	JN816604^3^	JN817024^3^
*Philodromus cespitum* (Walckenaer, 1802)	LEGO_42_4	Not informed	JN817235^3^	-	JN816602^3^	JN817023^3^
*Thanatus formicinus* (Clerck, 1758)	ARAMR000118	Uzbequistan (Farish)	KY017843^2^	KY018349^2^	-	KY017261^2^
*Thanatus* sp.	ARAMR000481	Senegal (Ndiass)	KY017844^2^	KY018350^2^	KY016029^2^	-
*Tibelloides taquarae **comb. nov**	ENT5400	Brazil (Paraná)	**OM773137**	**OM936924**	**OM913609**	**OM902672**
*Tibelloides reimoseri **nom. nov**	ENT5399	Paraguay (Itapúa)	-	**OM936920**	**OM913608**	**OM902671**
*Tibelloides bryantae **comb. nov**	ENT5000	Brazil (Pernambuco)	**OM773131**	**OM936919**	**OM913607**	-
*Tibelloides* sp. 1	ENT4997	Brazil (Minas Gerais)	**OM773128**	**OM936922**	**OM913605**	-
*Tibelloides* sp. 2	ENT4998	Brazil (Rio de Janeiro)	**OM773129**	**OM936923**	**OM913606**	-
*Tibelloides punctulatus **comb. nov**	ENT4994	Brazil (Rio de Janeiro)	**OM773127**	**OM936921**	**OM913604**	-
*Tibellus chamberlini* (Gertsch, 1933)	ARAMH000015	USA (Nevada)	KY017845^2^	KY018351^2^	KY01603^2^	KY017262^2^
*Tibellus oblongus* (Walckenaer, 1802)	ARAMR000102	Kazakhstan (Zhambyl)	KY017846^2^	KY018352^2^	KY016031^2^	KY017263^2^
*Titanebo mexicanus* (Banks, 1898)	ARAMR000106	USA (California)	KY017847^2^	KY018353^2^	KY016032^2^	KY017264^2^

^1^Polotow et al*.* (2015)

^2^Wheeler et al*.* (2017)

^3^Jang & Hwang (Unpublished)

### Alignment and phylogenetic analyses

Multiple alignments were conducted in MAFFT for ribosomal markers, with Q-INS, taking into account the secondary structure of RNA molecules [29, 30], and in CLUSTAL W in MEGA 7 [31] for other markers. The concatenated molecular data matrix included 3,020 bp (COI: 546, H3: 294, 16S: 320, 28S: 1,860).

Phylogenetic analyses under Maximum Likelihood (ML) and Bayesian Inference (BI) were conducted based on the concatenated molecular matrix with a partition scheme and respective evolutionary model selected by BIC [32] in ModelFinder [33] of IQ-TREE 1.6.12 [34]. Two separate analyses for model and partition scheme selection were conducted, the first for the ML analysis allowed testing of all models implemented in IQ-TREE including FreeRate [35, 36] for modeling heterogeneity across sites, and the second for the BI analysis limiting testing solely models that can be implemented in MrBayes. Initially, our dataset was partitioned by marker and codon (for coding genes) for a total of eight partitions. Both analyses resulted in a final seven-partition scheme reported in Additional file 3.

ML analysis of the concatenated dataset (partitioned and modeled as mentioned above) was performed in IQ-TREE (-lnL = 16,051.144). Clade support was inferred through 1,000 replicates of approximate Likelihood Ratio Test with the nonparametric Shimodaira-Hasegawa correction (SH-aLRT, [37]) and Ultrafast bootstrap (UFBoot, [38]).

BI analyses of the concatenated dataset (partitioned and modeled as mentioned above) and of each separate marker (Additional file 4) were performed in MrBayes 3.2.7 [39] through two independent searches, each with four Markov chains, for 50,000,000 generations, saving a tree every 5,000 generations. Of these, 12,500,000 generations were considered burn-in, corresponding to 12,501 trees. All analyses showed adequate convergence of independent analyses and parameter mixing, which were assessed by average standard deviation of split frequencies < 0.005, parameter Potential Scale Reduction Factor = 1.00, and ESS values > 10,000. Clade support of BI analysis is shown by Bayesian posterior probabilities (PP).

## Results

### Phylogenetic results

**Fig. 1:**
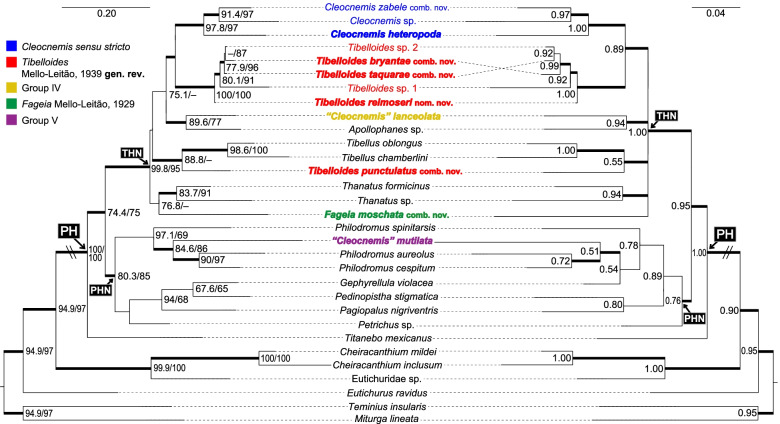
Maximum likelihood (-lnL = 16,051.144) (left) and Bayesian consensus (right) trees of Philodromidae and outgroups based on four molecular markers (COI, H3, 16S, and 28S). Terminal species formerly included in *Cleocnemis* are presented in bold type. Internal branches recovered in both analyses are thickened. Colors of terminals refer to representatives of species groups or genera that comprise species previously placed in *Cleocnemis*: blue for *Cleocnemis *sensu stricto; red for *Tibelloides* Mello-Leitão, 1939 **gen. rev.**; yellow for Group IV; green for *Fageia* Mello-Leitão, 1929; and purple for Group V. Clade support associated to each node are SH-aLRT/UFBoot (left) and Bayesian posterior probabilities (right). Abbreviations: PH, Philodromidae; PHN, Philodrominae; THN, Thanatinae

ML and BI topologies are very similar (Fig. 1) being incongruent only in the position of few taxa, e.g., *Petrichus* sp. and *Gephyrellula violacea*, and few clades that are not recovered in BI because of polytomies (see thick branches for clades recovered in both analyses in Fig. 1).

The eight species currently placed in *Cleocnemis *sensu lato included in our ML and BI phylogenetic analyses (see species in **bold type** in Fig. 1) were recovered into six distantly related lineages of running crab spiders. Six of the terminals are grouped in the Thanatini sensu Schick [6], herein treated as subfamily Thanatinae **new status** (THN in Fig. 1) with *Apollophanes*, *Fageia*, *Thanatus*, and *Tibellus*. This expanded concept of Thanatinae was recovered as monophyletic in both analyses, with high/moderate (SH-aLRT/UFBoot = 99.8/95) to maximum support (PP = 1.0). Only one of the species placed in *Cleocnemis *sensu lato, “*Cleocnemis*” *mutilata,* was grouped with *Gephyrellula*, *Pagiopalus*, *Pedinopistha*, *Petrichus*, and *Philodromus* in a modified Philodromini sensu Schick [6] (without *Titanebo*), herein treated as subfamily Philodrominae **new status** (PHN in Fig. 1). This clade had weak (SH-aLRT/UFBootBS = 80.3/85) to no significant support (PP = 0.76) in our analyses. Both analyses recovered *Titanebo* (inserted in Philodromini by Schick [6]) as the sister-group to the clade Thanatinae + Philodrominae. For name changes and new combinations, see 15 section and discussion below.

From the eight species formerly placed in *Cleocnemis *sensu lato and included in our phylogeny, only the type-species *Cleocnemis heteropoda* should remain as a valid species in the genus. It is grouped with *Cleocnemis* sp. and *Cleocnemis zabele* (Pantoja, Drago-Bisneto & Saturnino, 2020) **comb. nov.** (formerly *Berlandiella zabele*) in a clade called herein *Cleocnemis *sensu stricto (SH-aLRT/UFBoot = 97.8/97, PP = 1.00) for practical purposes. The other species of *Cleocnemis *sensu lato shall be transferred to other genera or considered *incertae sedis* (see Table 2)*.*

**Table 2:** Summary of taxonomic changes proposed herein concerning species placed in *Cleocnemis* previous to this study. Species and Taxonomic proposals columns also include the sexes (**♂♀)** known before and after this study respectively. “*” marks type species

Species	Taxonomic proposals	Synonyms
*Cleocnemis bryantae* (Gertsch, 1933) ♀	*Tibelloides bryantae* **comb. nov.** ♂♀	*Cleocnemis rudolphi* Mello-Leitão, 1943 **syn. nov**
*Cleocnemis heteropoda* Simon, 1886* ♂	♂♀	*Berlandiella polyacantha* Mello-Leitão,1929 **syn. nov** *Berlandiella meridionais* Mello-Leitão,1929 **syn. nov** *Metacleocnemis borgmeyeieri* Mello-Leitão, 1929 **syn. nov**
*Cleocnemis lanceolata* Mello-Leitão, 1929 ♀	***incertae sedis*** ♂♀	-
*Cleocnemis moschata* Mello-Leitão, 1943 J	*Fageia moschata* **comb. nov.** ♂♀	-
*Cleocnemis nigra* Mello-Leitão, 1943 ♂	***nomen dubium, incertae sedis***	-
*Cleocnemis paraguensis* (Gertsch, 1933) ♂♀	*Tibelloides reimoseri* **nom. nov. **♂♀	-
*Cleocnemis punctulata* Taczanowski, 1872 ♂♀	*Tibelloides punctulatus* **comb. nov.** ♂♀	*Tibelloides spatuliferus* Mello-Leitão, 1939 *Tibellus paraguensis* Simon, 1897 **syn. nov**
*Cleocnemis rosea* (Mello-Leitão, 1944) J	*Fageia rosea* **comb. nov.** J	-
*Cleocnemis spinosa* Mello-Leitão, 1947 ♂	***nomen dubium***	-
*Cleocnemis taquarae* (Keyserling, 1891) ♂♀	*Tibelloides taquarae* **comb. nov.** ♂♀	-
*Cleocnemis mutilata* Mello-Leitão, 1917 ♀	***incertae sedis*** ♂♀	*Gephyrina imbecilla* Mello-Leitão, 1917 **syn. nov**
*Cleocnemis xenotypa* Mello-Leitão, 1929 ♀	***nomen dubium, incertae sedis***	-
*Cleocnemis serrana* Mello-Leitão, 1929 ♀	***nomen dubium, incertae sedis***	-

“*Cleocnemis” bryantae*, “*Cleocnemis” reimoseri* (= *Cleocnemis paraguensis* (Gertsch, 1933)), and “*Cleocnemis” taquarae* are grouped together with two undescribed species as a highly supported monophyletic group in both analyses (SH-aLRT/UFBoot = 100/100, PP = 1.00). This group was recovered as sister to *Cleocnemis *sensu stricto in a poorly supported clade (SH-aLRT/UFBoot < 50, PP = 0.89). In ML, this clade arises as sister group of “*Cleocnemis” lanceolata* + *Apollophanes* (SH-aLRT/UFBoot = 89.6/77, PP = 0.94), with no significant support (SH-aLRT/UFBoot = 75.1/ < 50), and then relating to a clade containing “*Cleocnemis” punctulata* (= *Tibellus paraguensis* Simon, 1897) and the two *Tibellus* species (SH-aLRT/UFBoot = 88.8/ < 50), PP = 0.55), again with no significant support (SH-aLRT/UFBoot < 50). Relationships among these lineages of Thanatinae are poorly supported or unresolved in a polytomy in BI. Although “*Cleocnemis*” *punctulata* is recovered with the two *Tibellus* species with no significant support (SH-aLRT/UFBoot = 88.8/ < 50, PP = 0.55) in our molecular analyses, we are herein treating the former species as more related to the lineage of “*Cleocnemis*” *bryantae* + “*Cleocnemis*” *reimoseri* + “*Cleocnemis*” *taquarae* and two undescribed species cited before due to the striking morphological similarity of those species. The grouping of “*Cleocnemis*” *punctulata* and the other five species will compose the genus *Tibelloides* **gen. rev.** (see 11 section below).

Finally, the three other species formerly placed in *Cleocnemis *sensu lato, “*Cleocnemis” lanceolata, “Cleocnemis" moschata,* and “*Cleocnemis” mutilata*, were recovered as distantly related to other *Cleocnemis *sensu lato sampled. “*Cleocnemis*” *lanceolata* was recovered as sister to *Apollophanes* sp. (SH-aLRT/UFBoot = 89.6/77, PP = 0.94), while “*Cleocnemis*" *moschata* was placed in a clade with two species of Thanatus in ML (SH-aLRT/UFBoot = 76.8/ < 50) or in a polytomy with the other Thanatinae in BI. On the other hand, “*Cleocnemis*” *mutilata* was recovered as sister to two species of *Philodromus* (SH-aLRT/UFBoot = 84.6/86, PP = 0.51) in both analyses. Nevertheless, based on morphological characters, just “*Cleocnemis*” *moschata* is placeable in a Philodromidae valid genera and is herein transferred to *Fageia* (see 23 below). The other two species probably represent new genera, but are herein considered as *incertae sedis* pending further investigation.

### Morphological analysis and taxonomic treatment

Morphological analysis of somatic and genitalic characters allowed recognition of five different diagnosable informal species groups, which are discussed below, together with comments on species not recognizable or surely placed in a genus. We also include in those species groups some species we transferred herein from other genera of Philodromidae, besides the species previously placed in *Cleocnemis*.

Group I refers to *Cleocnemis heteropoda*, one undescribed species and five similar species currently placed in other genera of Philodromidae (see below). It corresponds to the redelimited *Cleocnemis *sensu stricto, for which we provide a new diagnosis and redescription of its type-species, *Cleocnemis heteropoda*. Despite *C. heteropoda*, the only current species of *Cleocnemis *sensu lato which is considered a member of *Cleocnemis *sensu stricto is *Cleocnemis spinosa* Mello-Leitão, 1947, but it is treated herein as *nomen dubium*. Group II is composed of *Cleocnemis taquarae* and similar species, herein included in *Tibelloides* **gen. rev.** This genus is removed from the synonymy with *Tibellus* and revalidated, for which we provide the redescription of *Tibelloides punctulatus* **comb nov.** and *Tibelloides bryantae* **comb nov.** *Cleocnemis moschata and C. rosea,* representing the Group III, are transferred to *Fageia*, and comments on this genus are provided. Two species represent Groups IV and V, respectively *Cleocnemis lanceolata* and *Cleocnemis mutilata* (senior synonym of *Gephyrina imbecilla* Mello-Leitão, 1917 **syn. nov**.), but are left as *incertae sedis* for now. Group V also includes *“Cleocnemis” xenotypa* Mello-Leitão, 1929, and *“Cleocnemis” serrana* Mello-Leitão, 1929, both species are considered *incertae sedis* and *nomina dubia*.

The only species of *Cleocnemis *sensu lato we are not sure about its identification or generic placement is *Cleocnemis nigra* Mello-Leitão, 1943, based on a male from Paraíba, Northeastern Brazil ([18], p. 169). The holotype was not found at MNRJ arachnological collection even before the tragic fire in 2018, and the short and little informative description, without any illustration, precludes even a tentative generic placement. So, “*Cleocnemis” nigra* is considered both as *nomen dubium* and *incertae sedis*.

Detailed discussion on the rationale for splitting *Cleocnemis*, as well as for transfers and synonymies, are given in the Discussion section. We provide under each taxon a bibliographic list limited to those that significantly contributed to diagnoses and composition of taxa, but a complete list of citations for genera and species is available at World Spider Catalog [1]. Additionally, a summary table with taxonomic changes proposed herein for all taxa previously included in *Cleocnemis* is provided in Table 2.

**Taxonomy**

Philodromidae Thorell, 1870.

#### ***Cleocnemis* Simon, 1886**

Figures 2, 3, and 4

**Fig. 2:**
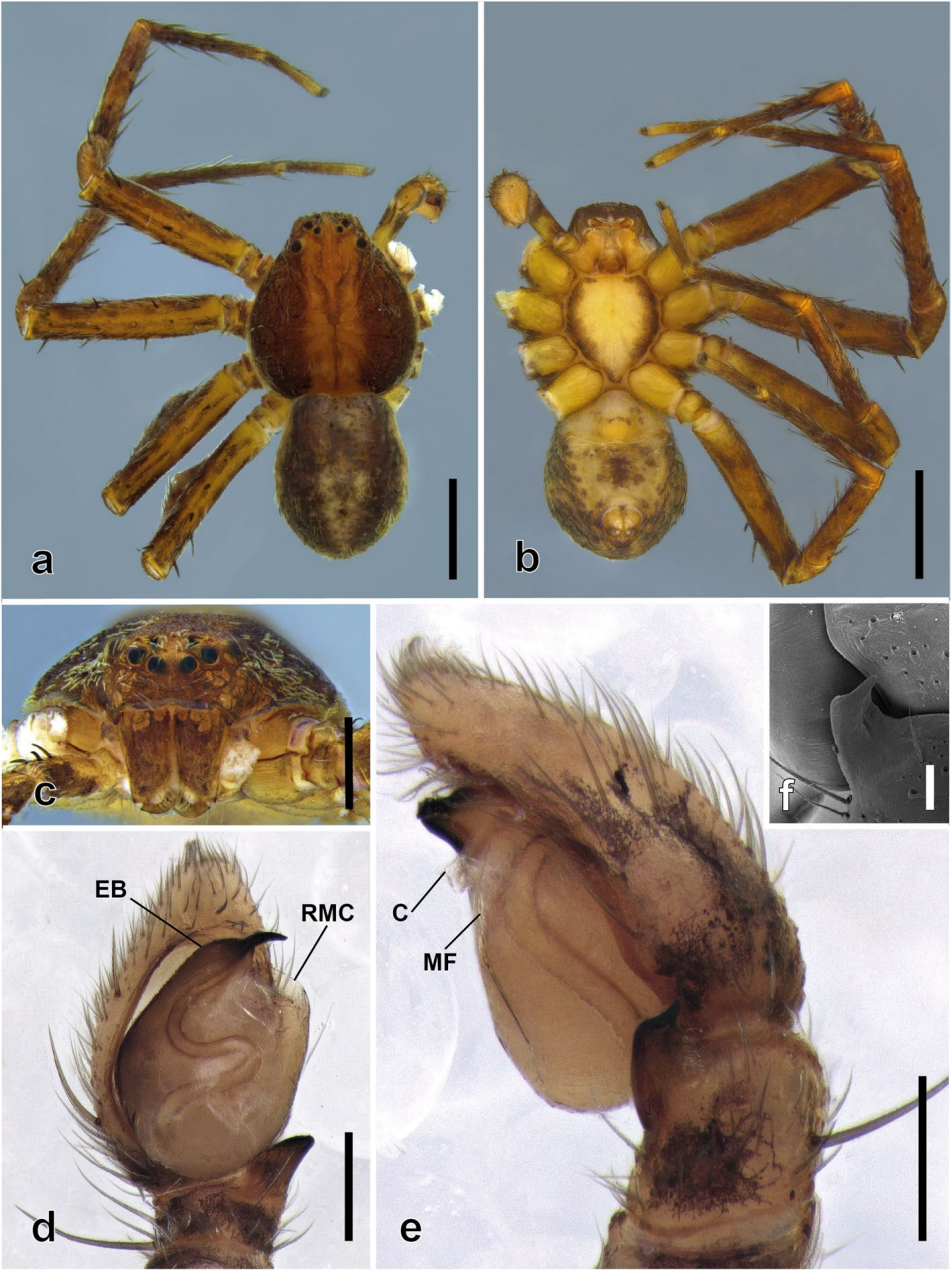
*Cleocnemis heteropoda* Simon, 1886, male. **a**, dorsal habitus; **b**, ventral habitus; **c**, cephalothorax frontal; **d–f**, left palpus (**d**, ventral; **e**, retrolateral; **f**, retrolateral tibial apophysis). **a–e**, (UFRJ 1562, neotype); **f**, (UFRJ 1637). Abbreviations: C, conductor; EB, embolic base; MF, membranous field; RMC, retrolateral marginal conductor. Scale bars: **a–c**, 1 mm; **d**,**e**, 0.2 mm; **f**, 0.05 mm

**Fig. 3:**
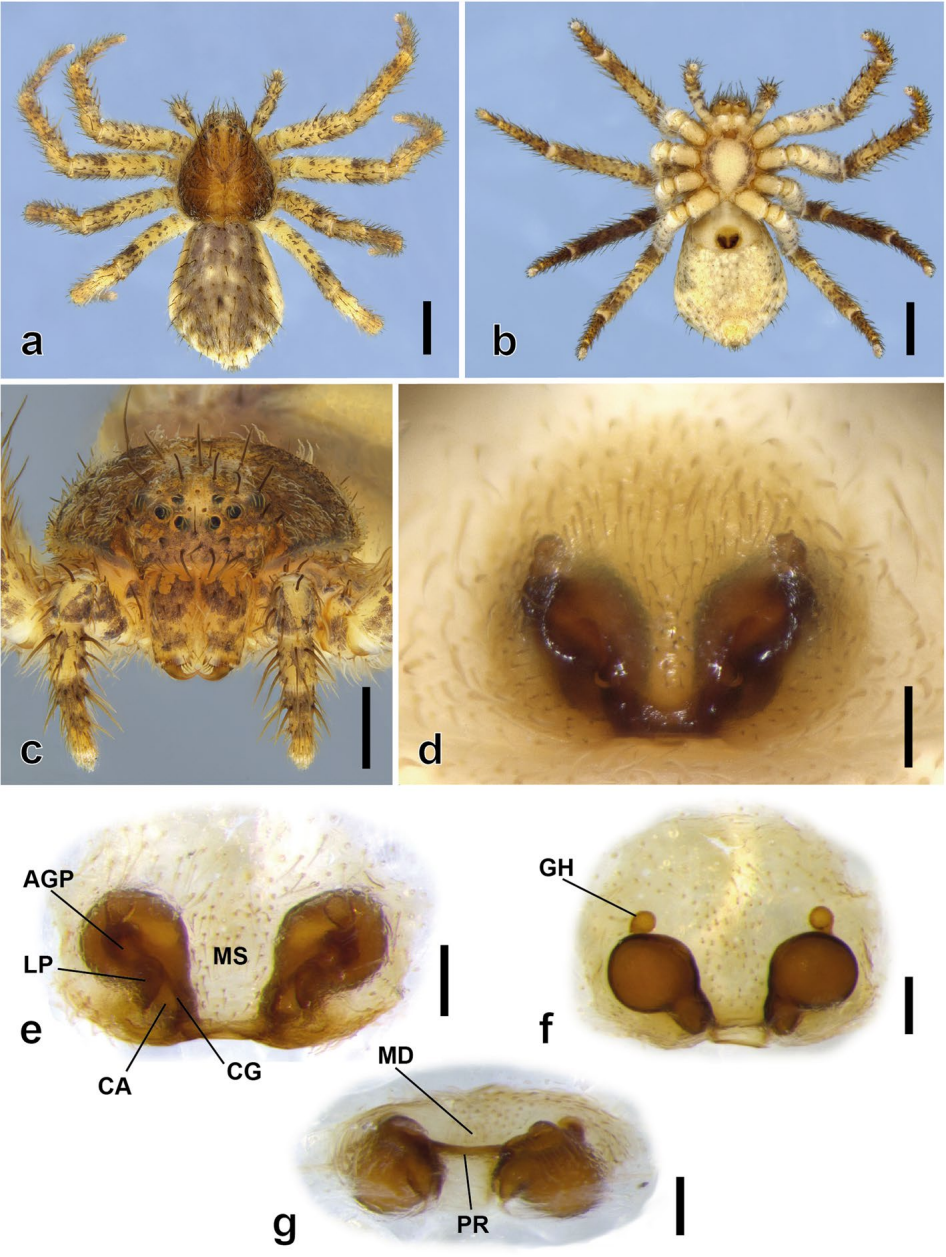
*Cleocnemis heteropoda* Simon, 1886, female. **a**, dorsal habitus; **b**, ventral habitus; **c**, cephalothorax frontal; **d**, epigynum; **e–g**, vulva (**e**, ventral; **f**, dorsal; **g**, posterior). **a–d**, (UFRJ 1630); **e–g**, (MNRJ 06525). Abbreviations: AGP, anterior guide pockets; CA, copulatory atria; CG, copulatory guides; GH, glandular head of spermatheca; LP, lateral plates; MD, mesal depression; MS, median septum; PR, posterior rim. Scale bars: **a,b**, 1 mm; **c**, 0.5 mm; **d–g**, 0.1 mm

**Fig. 4:**
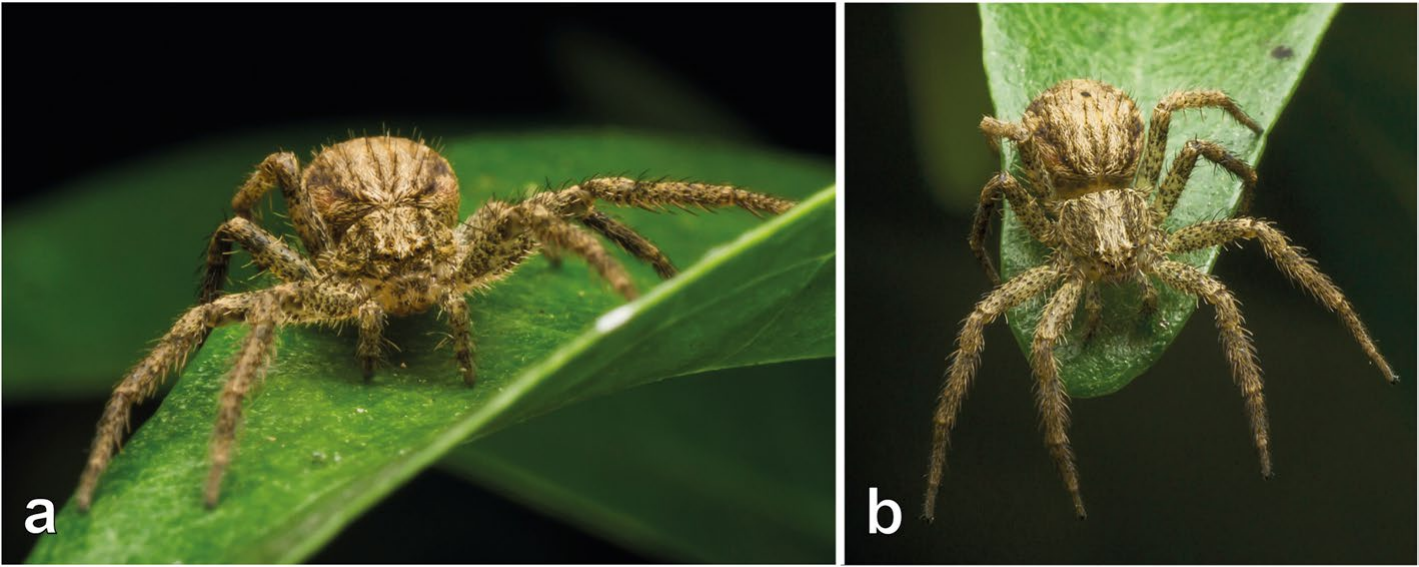
*Cleocnemis heteropoda* Simon, 1886, female (UFRJ 1638). Live specimen. **a**, frontal view; **b**, dorsal view. Photo credits: **a,b**, André Alves

*Cleocnemis* Simon, 1886: 186–187.

*Cleocnemis* Simon, 1895: 1064.; Mello-Leitão, 1929: 113–121; Dondale & Redner, 1975b: 1177.

*Metacleocnemis* Mello-Leitão, 1929: 121–122 **syn. nov**.

*Berlandiella* Mello-Leitão, 1929: 122–125 **syn. nov**.

*Berlandiella*: Lise & Silva, 2011: 350–371; Pantoja et al*.*, 2020: 1–13.

**Type-species**: *Cleocnemis heteropoda* Simon, 1886.

**Composition**: *Cleocnemis heteropoda* Simon, 1886, *Cleocnemis insignis* (Mello-Leitão, 1929) **comb. nov**., *Cleocnemis magna* (Mello-Leitão, 1929) **comb. nov**., *Cleocnemis querencia* (Lise & Silva, 2011) **comb. nov**., and *Cleocnemis robertae* (Lise & Silva, 2011) **comb. nov.,** and *Cleocnemis zabele* (Pantoja, Drago-Bisneto & Saturnino, 2020) **comb. nov**.

**Diagnosis**: *Cleocnemis* is a typical Thanatinae, with a male palp without tegular suture, paraconductor and with a simple membranous conductor, not massive or modified. It is similar to *Apollophanes* and *Paracleocnemis*, in general morphology, leg proportion (legs relatively short and robust, leg II longest), and color pattern (presence of dark lateral stripes in carapace and abdomen). It differs from *Apollophanes* and *Paracleocnemis* by the rigid setae spread over carapace, abdomen, and legs, and by the oblong, not elongated abdomen (at most $\frac13$ longer than wide). Females of *Cleocnemis* are distinguished from *Paracleocnemis* by carapace longer than wide, posterior eye row more recurved than the anterior one, epigyne with median septum reaching the epigastric furrow, or sometimes covered by enlarged lateral plates, but without a large deep concavity formed by fused posterior GP near the epigastric furrow. *Cleocnemis* is also separated from *Apollophanes* by palpus without VTA, embolus short, not curved ventrally, originating from a distinct embolic base, and with a flattened and elongated translucent conductor; epigynum with lateral plates projecting over or even hiding most of the central depression of septum, and glandular heads placed usually midway on the inner side of the main spermatheca or without an evident transition duct when placed closer to the anterior end of it (*C. heteropoda*).

**Description**: Total body length 2.84 mm (*C. querencia*) – 4.25 mm (*C. robertae*) in males and 3.05 mm (*C. robertae*) – 5.50 mm (*C. insignis*) in females. Carapace slightly longer than wide, usually wider between legs II and III, and narrowed anteriorly; background color usually brown, with paler wide longitudinal median band, and two wider and darker lateral bands, with many darker brown streaks; many covering setae, particularly on its margins, and long, erect bristles near the middle line, but more abundant in the eye region. Sternum approximately as long as wide, or slightly longer than wide; usually pale yellow and bordered with some black spots or stripes in most species. Labium usually wider than long, pale yellow to brown. Median ocular quadrangle (MOQ) variable, from a little wider than long (*C. heteropoda*) to a little longer than wide (*C. insignis*). Median eyes slightly smaller than lateral eyes, in general, with PLE usually larger than the others. Clypeus vertical or slightly slanted, ornated with a conspicuous set of macrosetae. Chelicerae with paturon pale yellow to dark brown. Legs yellow to dark brown, usually with dusky spots. Leg formula variable with second leg always longer than others, which are usually subequal. Femora, patellae, tibiae, metatarsi, and tarsi ornated with conspicuous erect macrosetae and bristles, tibiae I-II with four pairs of ventral macrosetae (also called spines) (only two in *C. querencia*), metatarsi I-II with three (sometimes two) pairs of ventral macrosetae. Scopula variable among species, occurring as ventrolateral scopulated setae along tarsi and metatarsi in *C. zabele* and *C. querencia*, whose abundance highly vary among individuals, while in other species it remains less conspicuous or absent. Trochanters with distinct set of bristles. Abdomen longer than wide, dorsally covered with erect bristles. Male palpi with small tibia, a little longer than wide, RTA variable, formed by a simple stem placed at the ventral angle of the upper margin of the retrolateral tibial face (*C. heteropoda*, *C. magna*) (Fig. 2e,f) to an excavated projection, laterally directed and inserted more medially at tibia (*C. robertae*, *C. querencia*, *C. zabele*), sometimes presenting a ventral lobe, also excavated and widely connected to the dorsal projection (*C. robertae*). Cymbium oblong, almost egg-shaped; with tip round and narrower than its middle, with a tuft of tenant hairs at the prolateral edge of its tip. Tegulum piriform, with base and middle region inflated and a thinner apical region with a distinct embolic base (EB) located at the prolateral edge of apical margin (Fig. 2d). In that region, a projected roundish mound forms the base of embolus, followed by a membranous field (MF), a membranous or poorly sclerotized concavity of variable size (sometimes collapsed) at the retrolateral region. Conductor translucent formed by thin membranous veil usually covering most of the retrolateral distal part of the tegulum, from near its retrolateral margin to the inner margin of the base of EB (Fig. 2d,e) but restricted to near retrolateral margin of tegulum in *C. zabele*, which has an inflated tegular mound filling the gap between the conductor and the EB. Retrolateral marginal conductor (RMC) placed near C at retrolateral margin, rounded, usually thin and translucent. Embolus originating from the embolar base at retrolateral upper edge of embolic base, forming a black projection, that varies from short and almost straight to relatively long and regularly curved claw (Fig. 2d, e). Sperm duct usually visible through most of its extension, forming initially a large curved tube near retrolateral margin of cymbium, disappearing near basal margin of tegulum and reappearing near prolateral basal margin of tegulum as a large S-shaped tube, with long median loop and its distal portion tapering and vanishing in black embolus (Fig. 2d, e). Epigynum very variable, with a very large mesal atrium that covers most of its area, forming a large and shallow mesal depression (MD) when the pair of elevated lateral plates (LP) are small and limited to sides of epigynal area (*C. heteropoda*) (Fig. 3d, e). An anterior atrium and posterior depressions associated with posterior GP are found in species with LP touching in the middle area (*C. querencia, C. robertae*) ([9], figs. 56, 106), a small anterior atrium in species with LP almost fused to each other (*C. insignis*); or just a posterior shallow depression when copulatory guides (CG) are fused anteriorly (*C. zabele*) ([11], figs. 4, 6). Posterior border of septum may be a little over or at the same level of the MD or strongly raised forming a high, almost vertical, posterior rim (PR) (*C. heteropoda*) (Fig. 3e–g). Copulatory openings (CO) usually at sides of central or anterior depression and followed by CG directed posteriorly (*C. heteropoda*) (Fig. 3d, e, g) or anteriorly (*C. zabele*) ([11], figs. 4, 6). There is an additional anterior guide pocket (AGP) at anterior region of the epigynum depression in *C. heteropoda* (Fig. 3 d, e). Spermathecae usually reniform, of variable size, placed at outer side of median or anterior depression; glandular heads rounded, with variable origin and position, usually midway at inner face of major spermathecae (*C. magna*, *C. zabele*) to anterior and outer margins of the same (*C. heteropoda*).

**Natural history**: Little is known about the natural history of the genus. Specimens are usually collected in closed and well-preserved forests, through sweeping and beating tray on bushes and lower branches of trees. In Teresópolis (Rio de Janeiro, Brazil) many specimens were beaten from branches above 5 meters high, near the top of small trees.

**Distribution:** *Cleocnemis* species are found throughout Brazil, from Rondônia and Pará states (Northern Region) to Rio Grande do Sul state (Southern Region), and from Mato Grosso and Mato Grosso do Sul states (Centralwestern Region) to Pernambuco and Paraíba states (Northeastern Region). It is also found in most regions of Paraguay and at Misiones province, northern Argentina.

**Taxonomic notes**: *Cleocnemis* is considered herein as a senior synonym of both *Berlandiella* Mello-Leitão, 1929 **syn. nov.** and *Metacleocnemis* Mello-Leitão, 1929 **syn. nov.** We were unable to find characters distinguishing *Cleocnemis heteropoda* from *Berlandiella polyacantha* Mello-Leitão 1929 **syn. nov.**, the latter described based on specimens from Teresópolis, a city close to Rio de Janeiro city, also in the State of Rio de Janeiro, Brazil. The many specimens collected and studied from Teresópolis are indistinguishable from specimens from Tijuca (type locality of *C. heteropoda*). *Berlandiella polyacantha* is undoubtedly related to *B. insignis* Mello-Leitão, 1929, type species of the genus, based also on specimens from Teresópolis. The differences pointed by Mello-Leitão ([15], p. 107) in his key to genera of “Philodrominas” from Brazil between *Cleocnemis* and *Berlandiella* are not accurate. Examination of photographs of lectotypes of *B. insignis* and *B. polyacantha* do not show a “sternum widely truncate behind” as stated by him, but a “sternum ending in an obtuse point”, as in *Cleocnemis*. The other difference pointed out by Mello-Leitão is the absence of scopula in leg tarsi, but this is a highly variable character among species of *Cleocnemis*.

The immature holotype of *Metacleocnemis borgmeyeieri* Mello-Leitão, 1929 was the only specimen of *Metacleocnemis* ever cited in literature. Mello-Leitão mentioned that the holotype was a female, but this is doubtful taking in account its small size (3.2 mm) and absence of any mention of an epigynum in the description. The holotype was not found in the MNRJ collection even before the 2018 fire. A careful analysis of the original descriptions of both the genus and species ([15], p. 121–122) and the good illustration of the habitus of the immature holotype ([15], fig. 38) revealed that it is clearly a *Cleocnemis* specimen, judging by general color pattern, leg proportion, and row of rigid clypeal setae. Even more, there is the striking agreement among details of the color pattern of the immature holotype and the usual color pattern of *C. heteropoda* (see Fig. 3, for example), the only other species of *Cleocnemis* found in the type-locality, Petrópolis. They share the predominant dark reddish-brown hues at lateral stripes of the carapace, dark grey V-shaped mark on the central brown stripe of the carapace, black hue at the beginning of dark brown lateral stripes of abdomen, and dark brown roundish mark at middle of the posterior portion of abdomen. Another species found in a nearby locality of Serra dos Órgãos mountains is *B. insignis*, from Teresópolis. However, *B. insignis* is a much paler species, with less extensive dark brown stripes on carapace and abdomen and lacking the median dark dot at dorsum ([9], fig. 2). So, we conclude that *Metacleocnemis borgmeyeieri* Mello-Leitão, 1929 **syn. nov.** is a synonym of *Cleocnemis heteropoda* Simon, 1886, and consequently we propose the generic synonymy between *Metacleocnemis* Mello-Leitão, 1929 **syn. nov.** and *Cleocnemis* Simon, 1886. In the key to Brazilian genera of Philodromidae ([15], p. 107), the only characteristic to set *Metacleocnemis* apart from *Cleocnemis* was “Olhos médios anteriores muito mais próximos um do outro que dos laterais” (= anterior median eyes much closer to each other than to the lateral ones), compared to anterior median eyes farther from each other than to the lateral ones in *Cleocnemis*. However, this difference is not clearly seen in the original illustration ([15], fig. 38) and may have been not as clear as stated in the original description. In contrast to the possible difference in eye position pointed by Mello-Leitão [15], our conclusion is supported by the notable resemblance in color pattern, leg proportion, and all other somatic characters cited in the original description.

Lise & Silva [9] described *Berlandiella meridionalis* in 2011 stating that “male and female of *B. meridionalis* sp. nov. […] are similar to the ones of *B. polyacantha*”, but that males could “be distinguished by the mesial face of the RTA being not excavated” and females by the “turned edge of the posterior margin and the excavation in front of it… and by the conspicuous accessory spermathecae in ventral view”. However, examination of many dozens of males from Southern and Southeastern regions of Brazil show that RTA excavation varies from deep to non-existent, although its presence is the rule in males from northern areas. Specimens with RTA excavation from Southern Brazil, such as those from Paraná (e.g., MCTP 39023, MCTP 39087) or Rio Grande do Sul states, are common. A RTA excavation may be found even in specimens from the type-locality of *B. meridionalis* (e.g., MCTP 19476). In relation to females, the diameter of the globular anterior portion of the main spermathecae is variable, with larger spermathecae hiding almost completely the GS, while smaller ones reveal most of the head of GS. Also, the shape of MS varies from U to V-shaped throughout the distribution area of *C. heteropoda*, in relation to the wider or narrower posterior MS. So, we propose that *Berlandiella meridionalis* Lise & Silva, 2011 **syn. nov.** is also a junior synonym of *Cleocnemis heteropoda* Simon, 1886.

In addition, other species previously placed in *Berlandiella*, *B. insignis*, *B. polyacantha* (= *C. heteropoda*), and the other four species recently described by Lise & Silva (2011) must all be transferred to *Cleocnemis*. An additional species transferred from *Berlandiella* is *Cleocnemis zabele* (Pantoja, Drago-Bisneto & Saturnino, 2020) **comb. nov.**, also from Brazil.

Besides the six valid *Cleocnemis* species, another species that clearly belongs to the genus is *Cleocnemis spinosa* Mello-Leitão, 1947, based on a male from Paraná state, southern Brazil, judging by its original description and pedipalp illustration ([20], p. 273, fig. 28). However, the holotype and only specimen known is considered lost (A. Silva, MNHCI, personal communication, September 15, 2020) and its identity cannot be currently determined. Thus, *Cleocnemis spinosa* Mello-Leitão, 1947 is herein considered a *nomen dubium*.

Summing up, based on present results, *Cleocnemis *sensu stricto includes six valid species from Brazil which present a very similar color pattern, general morphology, and genitalia. Besides, there is no need for a complete redescription of all *Cleocnemis* species, as they have been recently revised by Lise & Silva [9] (sub *Berlandiella*), who provided informative illustrations and details on morphology and genitalia.

***Cleocnemis heteropoda*** Simon, 1886.

Figures 2, 3 and 4

*Cleocnemis heteropoda* Simon, 1886: 186.

*Cleocnemis heteropoda*: Mello-Leitão, 1929: 115–116.

*Metacleocnemis borgmeyeri* Mello-Leitão, 1929: 121–122, fig. 38 **syn. nov.**

*Berlandiella polyacantha* Mello-Leitão, 1929: 125, figs. 128–129 **syn. nov.**

*Berlandiella meridionalis* Lise & Silva, 2011: 356, figs. 1, 26–51 **syn. nov.**

**Type-material:** *Cleocnemis heteropoda*: Male and immature female syntypes, BRASIL: **Rio de Janeiro**: Rio de Janeiro, Tijuca, Gounelle col. (MNHN, lost, not examined); Male neotype [**proposed herein**], BRASIL: **Rio de Janeiro**: Rio de Janeiro, Parque Nacional da Tijuca, 4.xii.2016, Eq. Lab. Entomologia UFRJ (UFRJ 1562). *Berlandiella polyacantha*: Female lectotype, 2 male and 4 female paralectotypes (after Lise & Silva, 2011), BRASIL: **Rio de Janeiro**: Teresópolis (MNHN 13783, examined by photographs). *Metacleocnemis borgmeyeri*: Female holotype, BRASIL: **Rio de Janeiro**: Petrópolis (MNRJ 923, Mello-Leitão Private Collection 802, lost, not examined).

**Additional material examined**: provided in Additional file 1.

**Diagnosis:** *Cleocnemis heteropoda* is the only species of the genus with epigynum bearing lateral plates small and not projected over the wide and deep mesal depression (MD) that occupies most of the female epigynal area, posterior rim strongly raised, almost vertical, guide pockets formed by an isolated pair of curved lobes at its anterior region (AGP) and glandular heads (GH) placed near the anterior border of the main spermatheca (Fig. 3e–g). Males may be easily recognized from *C. querencia* and *C. zabele* by the absence of a cymbial process (CP) (Fig. 2d,e) ([11], figs. 8–10, 31–33) and from *C. magna* and *C. robertae* by a much simpler RTA, with a small slanted point (Fig. 2d,e), but without a flattened and broad distal portion ([9], figs. 22–23, 62–70).

**Description. Male (Fig. ****2****) Neotype (UFRJ 1562).** Carapace slightly longer than wide, wider between legs II and III, narrowed anteriorly; background color pale brown, with a wide pale longitudinal median band, bearing a conspicuous V-shaped dark brown spot between the eye region and the fovea, and two wider dark lateral bands, with many darker brown streaks; many whitish covering setae, particularly on its margins, and long, erect dark brown bristles in a small number in the thoracic area and more abundant in the eye region (Fig. 2a). Clypeus vertical, mostly brown and with irregular pale brown spots on laterals, with a row of eight long erect bristles at its margin (only sockets left). Chelicerae with paturon dark brown with irregular pale brown spots near its base, with two prominent teeth on promargin, cheliceral mound with a set of bristles; fangs dark brown (Fig. 2c). Labium pale brown, suffused with black pigment; slightly wider than long, with rounded apex, clearly surpassing the middle of endites. Endites pale brown, with some black pigment near the outer border, with one depression on its inner margin near its base and another on its outer margin near its middle. Sternum with concave anterior margin and narrow and rounded posterior margin; pale brown, lighter than endites, with an irregular dark brown stripe at margins, except at anterior border (Fig. 2b).

Legs covered by different types of setae and many robust macrosetae disposed on femora, patellae, tibiae, and metatarsi; with four ventral pairs on tibia I–II and three ventral pairs on metatarsus I–II. Coxae and trochanters with a set of erect bristles at distal margin of dorsolateral face. Coxae pale brown, with dark brown pigments on its lateral faces, femora pale brown on dorsal face, a little darker on ventral face and with large dark brown markings laterally; patellae brown; tibiae and metatarsi mostly brown, but tibia and metatarsus III slightly darker, and tibia and metatarsus IV slightly lighter. Metatarsi with a pale brown ring at its tip. Tarsi mostly brown, but with the distal third pale brown. Tarsal claws unequal and pectinated; prolateral claw (mesal) with a row of 10 barely blunt teeth, very close to each other; and retrolateral claw (ectal) with only 3–4 thick triangular acute teeth spaced out from each other. Claw tuft with abundant scopulated setae, tarsi and metatarsi with sparse, long and thin trichobothria and without scopulae. Palpi pale brown, with dark brown spots at lateral faces.

Abdomen oval; longer than wide, a little wider at the posterior third; with a clear notch medially at anterior margin; densely covered with many greyish white covering setae and bearing dozens of long erect dark brown bristles (only reddish brown sockets left). Two pairs of reddish brown sigillae at dorsum near the outer border of cardiac mark. Dorsum with dark gray cardiac mark on a pale brown longitudinal band that tapers posteriorly. Two large lateral bands mostly dark grey, with a series of pale brown spots near the edges of dorsum (Fig. 2a). Lateral faces mostly dark gray, with few pale brown spots aligned in a median stripe. Venter pale yellow with irregular black spots. Conic spinnerets, anterior pair pale yellow and posterior pair with basal article abundantly mottled with dark gray and distal one yellow (Fig. 2b).

Measurements. Total length 3.50. Carapace 1.87 long, 1.71 wide, 0.62 high. Chelicerae 0.55 long, 0.28 wide. Clypeus 0.17 high. Labium 0.25 long, 0.28 wide. Endites 0.42 long, 0.26 wide. Sternum 0.97 long, 0.87 wide. Abdomen 1.62 long, 1.29 wide, 1.05 high. Leg: I. femur 1.73; patella 0.72; tibia 1.51; metatarsus 1.31; tarsus 0.62; total length 5.89; II. 2.14; 0.75; 1.91; 1.66; 0.82; 7.28; III. 1.80; 0.65; 1.36; 1.35; 0.61; 5.77; IV. 1.84; 0.64; 1.42; 1.40; 0.62; 5.92. Leg formula II > IV > I > III. Eye diameters and eye interdistances. AME 0.07, ALE 0.09, PME 0.07, PLE 0.08, AME–AME 0.13, AME–ALE 0.05, ALE–ALE 0.49, PME–PME 0.15, PME–PLE 0.22, PLE–PLE 0.64. MOQ 0.26 long in dorsal view, anterior width 0.26, posterior width 0.29.

Palpus (Fig. 2d–f). Tibia small, a little longer than wide, with one large curved macroseta near the retrolateral margin of the basal half of its dorsal surface, other at the same region near the prolateral margin, and an additional smaller prolateral macrosetae is placed distally and laterally to the above cited marginal macroseta. RTA placed at ventral edge of distal margin, formed by a robust dark stem, at ventral view with a triangular shape and a blunt tip, and at retrolateral view with a more rectangular shape and a distinct slanted acute projection distally at its dorsal edge, below which there is a conspicuous excavation (or notch). Cymbium oblong, almost egg-shaped, with tip round and narrower than its middle; with tuft of tenant hairs at prolateral edge of its tip; a large macroseta at basal third of prolateral face and another more displaced towards apex at retrolateral face; additional shorter macroseta near end of middle third, close to retrolateral border of alveolus. Tegulum piriform, with inflated base and middle region and thinner apical region, and with distinct embolic base (EB) located at prolateral edge of apical margin, that forms a projected roundish mound and is followed by a clear MF concavity at retrolateral region. Conductor membranous (C) formed by thin transparent veil covering from near distal retrolateral margin of cymbium to embolic base. Retrolateral marginal conductor (RMC) between C and the retrolateral margin of cymbium, rounded, thin and translucent. Embolus (E) black, forming a relatively long and regularly curved claw, pointed retrolaterally, and originating from retrolateral upper edge of the EB. Sperm duct clearly visible through most of its extension, forming initially a large curved tube near retrolateral margin of cymbium, disappearing near the lower margin of tegulum and reappearing near prolateral lower margin of tegulum as a large S-shaped tube, with long median loop and distal portion tapering and vanishing in black embolus.

**Description. Female (Fig. ****3****) (UFRJ 1630).** Color and structure usually as in male, but general color paler. Legs mostly yellow, with many dark grey spots all over, except tibia, metatarsus III and patellae which are mostly dark brown, particularly ventrally. Palpi yellow, with scattered dark grey spots.

Measurements. Total length 4.76. Carapace 2.06 long, 1.93 wide, 0.89 high. Chelicerae 0.62 long, 0.32 wide. Clypeus 0.23 high. Labium 0.27 long, 0.24 wide. Endites 0.46 long, 0.31 wide. Sternum 1.16 long, 0.89 wide. Abdomen 2.68 long, 2.16 wide, 1.78 high. Leg: I. femur 1.56; patella 0.78; tibia 1.34; metatarsus 1.16; tarsus 0.61; total length 5.45; II. 1.83; 0.83; 1.54; 1.37; 0.67; 6.24; III. 1.67; 0.71; 1.29; 1.21; 0.58; 5.46; IV. 1.74; 0.66; 1.23; 1.27; 0.58; 5.48. Leg formula II > IV > III > I. Eye diameters and eye interdistances. AME 0.07, ALE 0.07, PME 0.07, PLE 0.08, AME–AME 0.17, AME–ALE 0.07, ALE–ALE 0.41, PME–PME 0.22, PME–PLE 0.24, PLE–PLE 0.73. MOQ 0.24 long in dorsal view, anterior width 0.27, posterior width 0.33.

Epigynum (Fig. 3d–e,g) wider than long; with  median septum (MS) bearing a wide and deep mesal depression (MD), occupying most of the epigynal area, and abruptly ending close to the epigastric furrow (or genital groove) in a sclerotized, raised posterior rim, which is thin and relatively wide, reaching less than half of the width of the epigynal area. Lateral plates (LP) small and placed far laterally, forming small roundish lobe at posterior half of epigynal area, covering anterior part of copulatory guides (CG) and copulatory atria (CA). CG describing a slanted arch, with visible portion going from the posterior margin of LP to posterior margin, where it merges with posterior rim of MS, and with what appears to be a hidden portion going from below the LP anteriorly up to the anterior guide pockets (AGP) and the probable location of CO near the base of the GH stalk. AGP placed laterally at anterior half of epigynal area, formed by a comma-like elevated notch. The set formed by the wide MD and bordered by AGP, LP and posterior rim has a “U” shaped form. There is an evident more or less triangular fovea at each side delimited by the overlapping of lobed LP and keel of CG, forming a foveal furrow conducting to CO. Spermathecae (S) large, piriform, placed at each side of the MD, with an anterior spheric and large portion and a smaller and much thinner posterior cylindrical portion. Glandular head (GH) placed ventrally to S, with its origin hidden by the AGP, being small and piriform and with its head surpassing the anterior margin of S and clearly seen at dorsal view (Fig. 3e,f).

**Variation**: Length variation: 4.33 to 4.92 in females (n = 10) and 3.42 to 3.88 in males (n = 10). Some specimens are much darker in color pattern, especially males. MOQ variable, rarely slightly longer than wide, but usually slightly wider than long. General appearance of MD may vary from the typical U-shaped to a more V-shaped area, due to narrower posterior rim. GH may be almost completely hidden in dorsal view, due to variation in size of cylindrical part of S, that may vary in diameter. RTA with slanted point of variable length and width; most specimens from northern areas of distribution bear an evident distal excavation under the slanted point of the RTA, but specimens from southern areas have excavation varying from clearly seen to absent, sometimes even in specimens from same collecting event.

**Distribution:** This species is found in a long but narrow area near the Atlantic coast of Southeastern and Southern regions of Brazil, with its northern limit in southern Minas Gerais State, eastern at central Rio de Janeiro State, western at central-east Paraná State, and southern limit at northern Rio Grande do Sul State. The male from Santarém, Pará State, northern Brazil, deposited at MNHN, is obviously wrongly labeled, as all other specimens were collected at Southeastern and Southern regions of Brazil.

**Taxonomic notes:** The need for designation of a neotype for *Cleocnemis heteropoda* is clear, following article 75 of the International Code of Zoological Nomenclature [40]. In particular, all of the qualifying conditions (article 75.3) are fulfilled, as the designation of the neotype: (1) will help to clarify the taxonomic status of the species and the genus, (2) is accompanied by a diagnosis and redescription of the taxa, (3) replaces the type series that was not found in MNHN despite several searches, and (4) make it sure that the specimen agrees well with the original description and comes from the type locality. Details about the synonymies of *Berlandiella meridionalis*, *Berlandiella polyacantha*, and *Metacleocnemis borgmeyeri* are above in the the Taxonomic notes of the genus, while detailed notes on the careful process to assure the true identity of *C. heteropoda* are given in the discussion section below.

#### ***Fageia* Mello-Leitão, 1929**

*Fageia* Mello-Leitão, 1929: 113.

**Type species:** *Fageia amabilis* Mello-Leitão, 1929.

**Composition:** *Fageia amabilis* Mello-Leitão, 1929, *Fageia concolor* Mello-Leitão, 1947, *Fageia meridionalis* Mello-Leitão, 1943, *Fageia moschata* (Mello-Leitão, 1943) **comb. nov.**, and *Fageia rosea* (Mello-Leitão, 1944) **comb. nov.**

**Diagnosis:** *Fageia* is easily recognized by the wide and flattened carapace and by the large, flat, and pentagonal abdomen, with many conspicuous spatulated setae. Its palpus has a translucent or light colored thin VTA pressed against the base of the RTA, which is shaped as a slanted, triangular, brown lobe of variable size. The epigynum has a large V or U-shaped MS, which harbors the large and deep MD and with the copulatory openings at its anterior angles, CG long and not quite elevated, following the anterior edges of the MS, and large and deep posterior GP at each side of the MS, near the epigastric furrow.

**Taxonomic notes:** *Fageia* is currently being reviewed and a paper with redescriptions and distribution updates will be published soon (H. Schinelli et al*.*, in prep.). Here, we only transfer to *Fageia* species formerly placed in *Cleocnemis* and add some comments on diagnosis, composition, and distribution of the genus. Additional notes and details about the synonyms are found in the discussion section below.

**Distribution**: Previously known only from Brazil, from Bahia (Northeastern region) to Rio Grande do Sul (Southern Region). With the inclusion of the species herein transferred and new records, the genus distribution needs to be expanded north up to Amazonas and Roraima states (Northern Region), west up to Mato Grosso state (Centralwestern Region), and south up to Paraguay and Argentina.

#### *Tibelloides* Mello-Leitão, 1939 gen. rev.

**Fig. 5:**
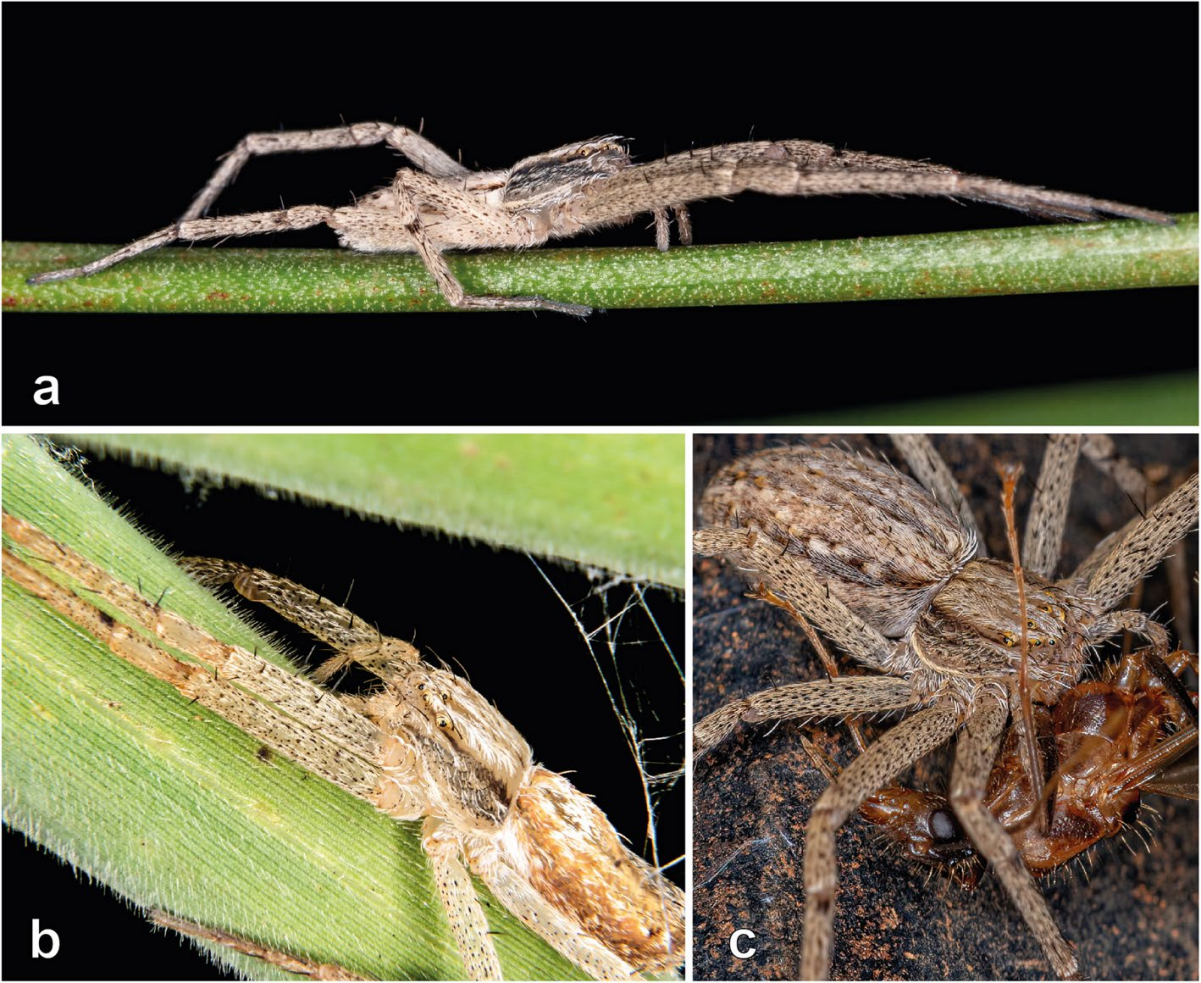
*Tibelloides* sp. 2, live specimens from Itajá, Goiás, Brazil. **a**, **b**, adult females in typical cryptic posture; **c**, adult female preying. Photo credits: **a–c**, Vinícius Souza

**Fig. 6:**
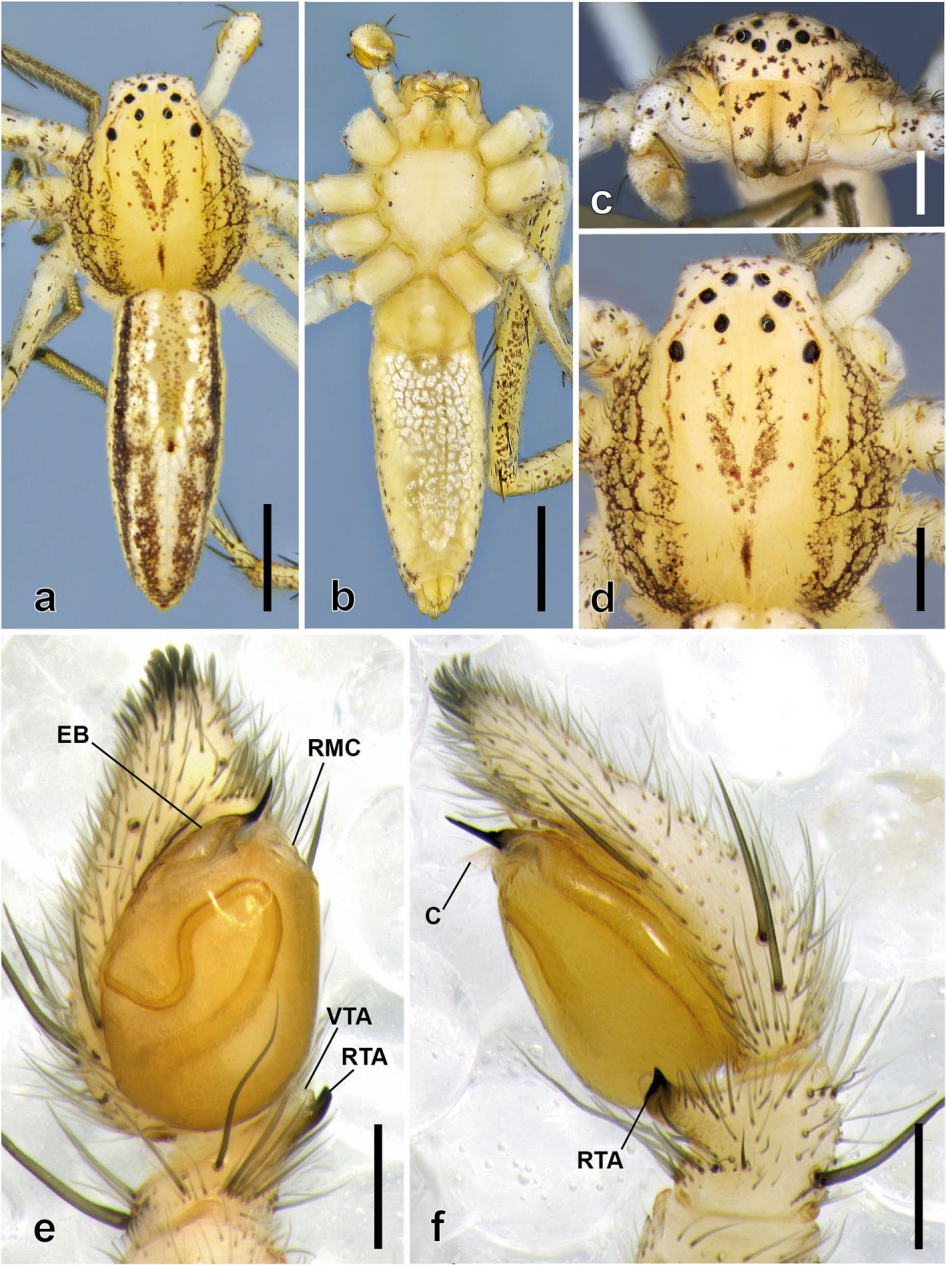
*Tibelloides bryantae* (Gertsch, 1933) **comb. nov.**, male (UFRJ 1561). **a**, dorsal habitus; **b**, ventral habitus; **c**, cephalothorax frontal; **d**, cephalothorax dorsal. **e**,**f**, left palpus (**e**, ventral; **f**, retrolateral). Abbreviations: C, conductor; EB, embolic base; RMC, retrolateral marginal conductor; RTA, retrolateral tibial apophysis; VTA, ventral tibial apophysis. Scale bars: **a**,**b** 1 mm; **c**,**d** 0.5 mm; **e**,**f**, 0.2 mm

**Fig. 7:**
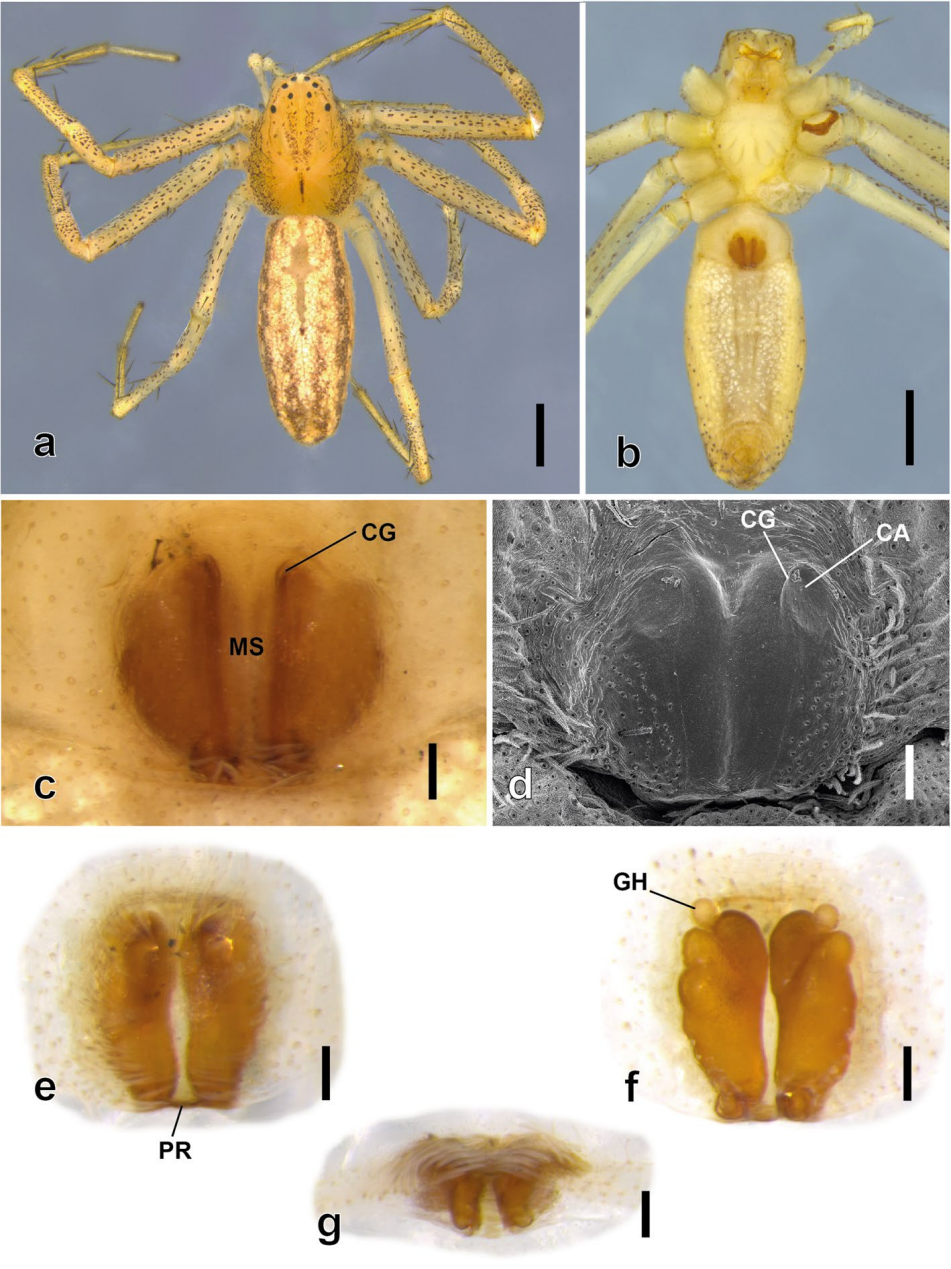
*Tibelloides bryantae* (Gertsch, 1933) **comb. nov.**, female. **a**, dorsal habitus; **b**, ventral habitus; **c**,**d**, epigynum; **e–g**, vulva (**e**, ventral; **f**, dorsal; **g**, posterior). **a–c**, (UFRJ 1560); **d**, (UFRJ 2001); **e–g** (IBNP 2978). Abbreviations: CA, copulatory atria; CG, copulatory guides; GH, glandular head of spermatheca; MS, median septum; PR, posterior rim. Scale bars: **a**,**b**, 1 mm; **c–g**, 0.1 mm

**Fig. 8:**
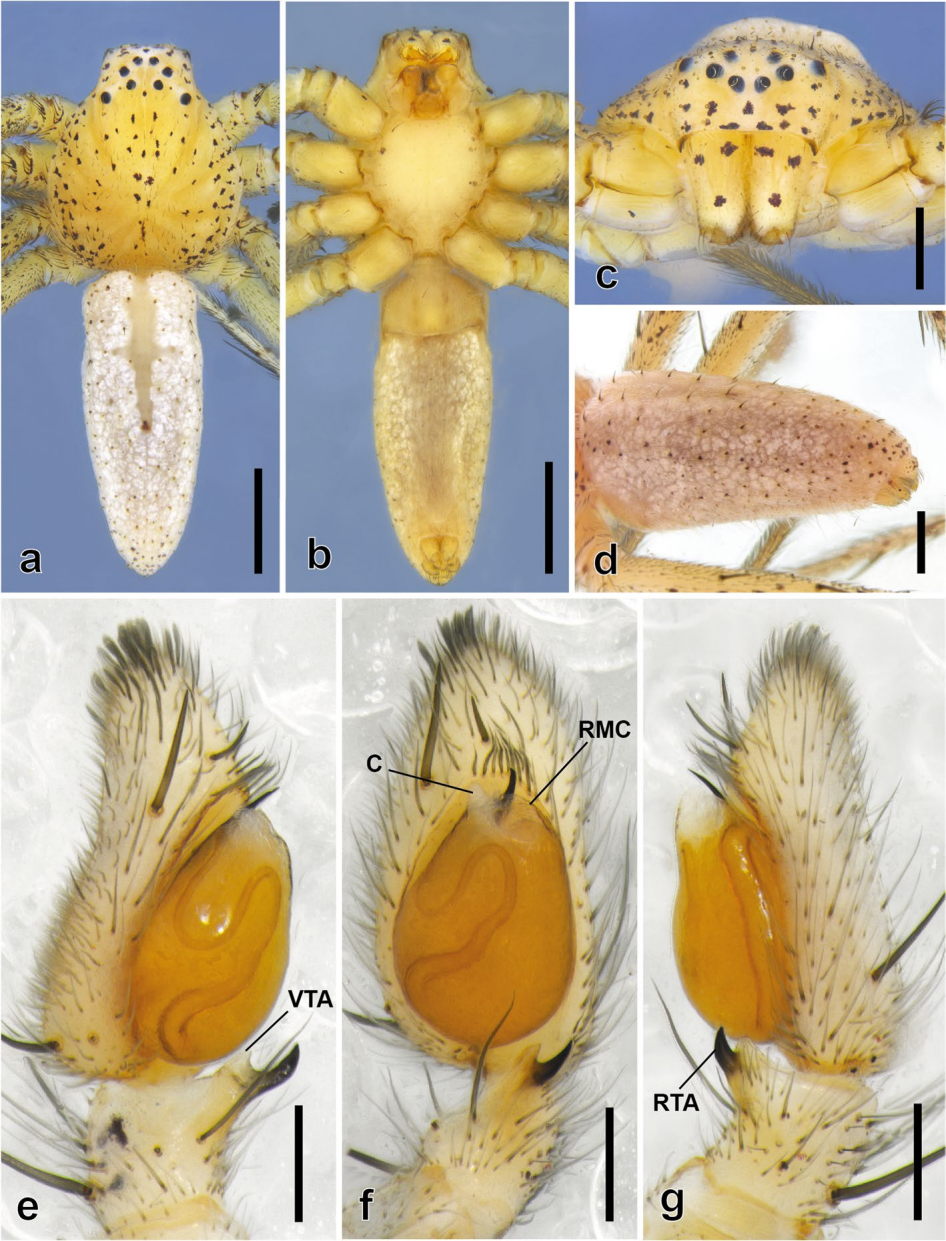
*Tibelloides punctulatus* (Taczanowski, 1872) **comb. nov.**, male (UFRJ 1971). **a**, dorsal habitus; **b**, ventral habitus; **c**, cephalothorax frontal; **d**, abdomen lateral. **e–g** left palpus (**e**, prolateral slightly slanted; **f**, ventral slightly slanted; **g**, retrolateral). Abbreviations: C, conductor; RMC, retrolateral marginal conductor; RTA, retrolateral tibial apophysis; VTA, ventral tibial apophysis. Scale bars: **a**,**b** 1 mm; **c**,**d** 0.5 mm; **e–g**, 0.2 mm

**Fig. 9:**
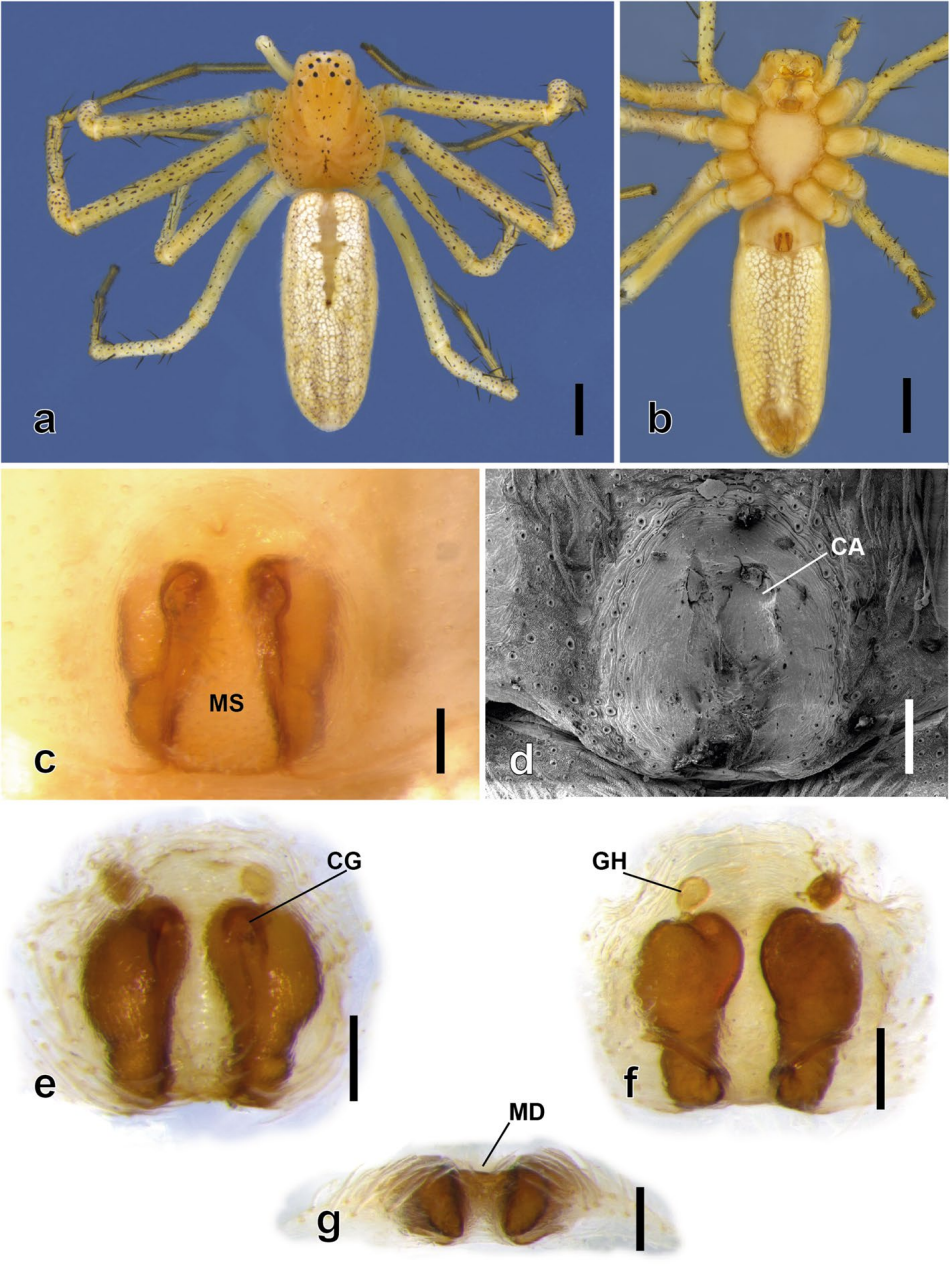
*Tibelloides punctulatus* (Taczanowski, 1872) **comb. nov.**, female. **a**, dorsal habitus; **b**, ventral habitus; **c**,**d**, epigynum; **e–g**, vulva (**e**, ventral; **f**, dorsal; **g**, posterior). **a–c**, (UFRJ 1972); **d**, (UFRJ 0484); **e–g**, (UFRJ 0486). Abbreviations: CA, copulatory atria; CG, copulatory guides; GH, glandular head of spermatheca; MD, mesal depression; MS, median septum. Scale bars: **a**,**b**, 1 mm; **c–g**, 0.1 mm

Figures 5, 6, 7, 8 and 9

*Thanatus*: Taczanowski, 1872: 73 (in part).

*Tibellus*: Keyserling, 1880: 197 (in part).

*Cleocnemis*: Mello-Leitão, 1929: 114 (in part).

*Apollophanes*: Gertsch, 1933: 14 (in part).

*Tibelloides*: Mello-Leitão, 1939: 76–77.

*Tibellus:* Mello-Leitão, 1945: 224 (S); Achitte-Schmutzler & Rubio, 2016: 146.

**Type species**: *Tibelloides spatuliferus* Mello-Leitão, 1939 (= junior synonym of *Tibelloides punctulatus* (Taczanowski, 1872) **comb. nov.**).

**Composition**: *Tibelloides bryantae* (Gertsch, 1933) **comb. nov**., *Tibelloides punctulatus* (Taczanowski, 1872) **comb. nov.**, *Tibelloides reimoseri* **nom. nov**. (new name for *Apollophanes paraguensis* Gertsch, 1933), and *Tibelloides taquarae* (Keyserling, 1891). **comb. nov**.

**Diagnosis**: Species of *Tibelloides* resemble the typical Holarctic species of *Tibellus* in general morphology, with long body and legs, but they lack the usual median longitudinal dark stripe over the whole length of carapace and abdomen found in that genus. *Tibelloides* species have instead a large pale median longitudinal band on carapace, with a V-shaped dark mark or just irregular dark dots (Figs. 5b,c, 6a,d, 7a, 8a and 9a). On the abdomen, *Tibelloides* usually have a cardiac mark surrounded by dark spots (Figs. 5b,c, 6a, 7a, 8a and 9a). Males of *Tibelloides* bear a conspicuous RTA, consisting of an elongated sclerotized rod projecting over the cymbium, sometimes with a larger rectangular or rounded expanded lobe (Figs. 6e,f and 8e–g), while *Tibellus* usually has no RTA or sometimes a rudimentary one, mostly membranous and not clearly delimited from the tibial rim. In females of *Tibelloides*, the epigynum is placed wholly in a concave, somewhat darkened epigynal area, with median septum (MS) elongated and presenting lateral borders raised over a shallow mesal depression. Copulatory guides placed usually over the inner margin of the anterior portion of the main spermathecae and curved around the copulatory atria, which bears the copulatory openings (CO) (Figs. 7c–e and 9c–e). The epigynum of *Tibelloides* is clearly separated from that of typical *Tibellus* species, which presents MS large and raised and CO placed near the epigastric furrow and inside deep lateral pockets. The vulva also is differentiated as *Tibelloides* have main spermathecae large, elongated, and with grooves and GH originates at its anterior half, while *Tibellus* have spermathecae round to oval, not enlarged or grooved, with GH originating at its posterior half.

**Description**: Total body length 4.00 mm – 7.20 mm in males and 4.05 mm – 7.92 mm in females. Carapace longer than wide, usually wider at the level of leg III, and narrowed anteriorly; background color usually yellow, a paler wide longitudinal median band with a V-shaped dark mark, or irregularly disposed dark spots between two wider and darker lateral bands, with many darker brown streaks; many covering setae, particularly on its margins, and a set of long macrosetae at the eye region. Sternum slightly longer than wide; usually pale yellow and bordered with some dark spots in some species. Labium generally wider than long, yellow, and usually with dark spots. Median eyes slightly smaller than lateral eyes, in general, with PLE usually larger than others (Figs. 6a,b, 7a,b, 8a,b and 9a,b).

Clypeus vertical ornated with set of long macrosetae. Chelicerae with paturon yellow with dark spots. Legs yellow, metatarsi and tarsi usually darker, and with many dark spots, leg formula variable, II > I > IV > III, II > IV > I > III or II > I = IV > III. Femora, patellae, tibiae, metatarsi and tarsi ornated with conspicuous erect macrosetae and bristles, tibiae I-II with 2 to 3 pairs of ventral macrosetae (or spines), metatarsi I-II with 2 pairs of ventral macrosetae. Trochanters with distinct set of bristles. Abdomen longer than wide, two times longer or more in some species (*T. bryantae* and *T. reimoseri*), (Figs. 6a and 7a) dorsally covered with erect bristles (Fig. 8d). Male palpus with small tibia, almost as long as wide; VTA small and poorly sclerotized, represented by light lobe pressed against RTA or only small projection in concavity at inner side of the RTA, shaped as a simple globular lobe (*T. bryantae* and *T. taquarae*) (Fig. 6e,f), sinuous keel (*T. reimoseri*), or being more sclerotized and forming a small claw (*T. punctulatus*) (Fig. 8e–g); RTA thin and elongate, thoroughly sclerotized and projected in acute or blunt point. Cymbium oblong, almost egg-shaped; with tip round and narrower than its middle, with tuft of tenant hairs at prolateral edge of its tip. Tegulum discoid in ventral view, but wider at base and thinner at the apex in lateral view, with an almost water drop-like shape. Embolus emerges from distinct EB located at prolateral edge of apical margin, shaped as projected roundish mound, that is followed by concavity of variable size at retrolateral region. Embolus originates from the retrolateral distal edge of the EB, forming a black projection that varies from short and almost straight to relatively long and regularly curved claw. MF occupying most of retrolateral half and extending to prolateral side of distal cymbial area just before EB. Conductor membranous formed by thin transparent veil covering from near the distal margin of the cymbium to the base of EB. Retrolateral marginal conductor (RMC) between C and the retrolateral margin of cymbium, rounded, usually thin and translucent. Sperm duct usually visible through most of its extension, forming initially large curved tube near the retrolateral margin of the cymbium, disappearing near the basal margin of tegulum and reappearing near the prolateral lower margin of tegulum as sinuous S-shaped tube, wider at its long median loop and tapering in its distal portion, vanishing in the black embolus (Figs. 6e,f and 8e–g). Epigynum with a very large mesal atrium that covers most of its area, forming a large and shallow mesal depression (MD), MS elongated, varying from very wide to thin, shaped as long rectangle (*T. bryantae*) (Fig. 7c–e), a trapezoid wider at its posterior (*T. punctulatus*) (Fig. 9c–e) or anterior (*T. reimoseri*) extremity, or with large concavities at its median part which may even touch each other at the middle line (*T. taquarae*), MS with sclerotized, raised posterior rim, which is thin, almost vertical and relatively wide, reaching less than half of the width of the epigynal area. CG usually thin and short, appearing as a comma-like keel, placed over the anterior inner border of main spermathecae (S). Copulatory openings (CO) placed near anterior edge of S, within rounded or elliptic copulatory atria (CA). Guide pockets or lateral plates absent. S longer than wide, with similar width throughout, or with rounded extremities, but also may present a clear basal constriction (*T. punctulatus*) (Fig. 9c,e,f). Glandular head of spermathecae with constricted base and a globular head (Figs. 7f and 9f), placed near to anterior edge of S, but varying its insertion point from near middle line to close to outer border.

**Natural history**: Most specimens were found in grasslands, commonly near to anthropized areas or open forests, where they are sampled mainly through sweeping (Fig. 5a–c). Specimens assume a cryptic posture on grass leaves, stretching the legs I, II and IV, and bending legs III (which are typically smaller than others) (Fig. 5a,b), a behavior also observed in species of *Tibellus*.

**Distribution.** The five described species of *Tibelloides* are found from northern Venezuela and Guiana, through Brazil, Bolivia, Paraguay, and Uruguay, to Argentina. There are also unverified literature records from Peru ([41], p. 236; [42], p. 263). We examined additional specimens from Chile (IBSP).

**Taxonomic notes**: We have not been able to find any significant genitalic or morphological character to tell apart *Tibelloides punctulatus* **comb. nov.** from the five species included in unrelated lineage comprising “*Cleocnemis*” *taquarae* recovered in our molecular phylogenies*.* Therefore, we treat all six species as belonging to the newly revalidated *Tibelloides* **gen. rev.**, notwithstanding the conflicting results of the molecular phylogeny.

*Tibelloides punctulatus* **comb. nov.** is transferred from *Tibellus* to *Tibelloides* **gen. rev.** and newly proposed as senior synonym of *Tibellus paraguensis* Simon, 1897 **syn. nov.** The strong similarity of *Tibelloides punctulatus* **comb. nov.** and *Tibellus paraguensis* had been already noticed by Gertsch ([43], p. 10). The last species was already considered a senior synonym of *Tibelloides spatuliferus* Mello-Leitão, 1939, the type-species of *Tibelloides* by monotypy.

*Tibelloides spatuliferus* was based on a female from Paraguay ([44], p. 76). Later, Mello-Leitão ([45], p. 224) himself synonymized *Tibelloides* with *Tibellus* without further explanation, by just including *Tibelloides spatuliferus* as junior synonym of *Tibellus paraguensis* in a list of species from some Argentinian provinces. This species was also described from Paraguay and was previously recorded from Bolivia and Argentina [1]. *Tibellus paraguensis* was recently redescribed by Achitte-Schmutzler & Rubio [22], which newly described the male and provided many new records of that species.

Examination of photos of the holotype of *Tibelloides spatuliferus* and abundant specimens of *Tibellus paraguensis* from Paraguay allowed us to confirm the previous synonymy and to propose *Tibellus paraguensis* Simon, 1897 **syn. nov.** as a junior synonym of *Tibelloides punctulatus* **comb. nov.** (described as *Thanatus punctulatus* Taczanowski, 1872) (photos of type specimens and abundant material examined), a common species recorded from northern Brazil to Argentina. This species was also erroneously included in *Cleocnemis *sensu lato and clearly belongs to the Group II (= *Tibelloides* **gen. rev.**).

In relation to the implicit generic synonymy of the genus *Tibelloides* with *Tibellus*, we do not agree with Mello-Leitão [45], taking in account the clear-cut somatic and genitalic gap between *Tibelloides punctulatus* **comb. nov.** (= *Tibellus paraguensis* **syn. nov.**) plus remaining species of Group II, all from Neotropical region, and the typical *Tibellus*, mostly from Holarctic region.

*Tibellus* is one of the largest genera of Philodromidae, with 50 described species, most of which are Holarctic, but with 17 African, one Australian, and five Neotropical species [1]. Some Holarctic species are pretty common and well-known, as the type species *Tibellus oblongus* (Walckenaer, 1802) and *Tibellus duttoni* (Hentz, 1857). Contrasting to *Tibelloides*, the typical species of *Tibellus* are predominantly from the Holarctic region and share a series of diagnosable characters, such as, presence of a dark longitudinal median stripe throughout the body; RTA absent or reduced to a small lobe; epigynum with large median septum (or median plate) and two large lateral copulatory bursae, containing the copulatory openings, which are placed posteriorly, closer to the epigastric furrow than to the anterior margin of the epigynum; and glandular heads placed at outer sides of the main spermathecae and usually with a thin stalk-like duct (see [46–48]). Some African species may lack one or more of these characteristics, such as the median stripe or the lateral position of the glandular heads (for examples, see [48]).

With the removal of *Tibellus paraguensis*, only four Neotropical species described in *Tibellus* remain in the genus: *Tibellus affinis* O. Pickard-Cambridge, 1898 (immature female, Mexico), *Tibellus chilensis* Mello-Leitão, 1943 (female, Chile), *Tibellus insularis* Gertsch, 1933 (female, Cuba), and *Tibellus spinosus* Schiapelli & Gerschman, 1941 (female, Argentina). Unfortunately, these four Neotropical *Tibellus* have not been collected or recorded again after their original description [1]. Only *Tibellus affinis* and *Tibellus insularis* seem typical *Tibellus*, with dark median stripe in the body and posterior copulatory openings. Also, *Tibellus insularis* is similar to *Tibellus duttoni* (Hentz, 1847) after Gertsch [49]. The other two Neotropical species are not easily placed in described genera or recognized due to the lack of information in the original descriptions. Summing up, there is no published confirmed record of a typical *Tibellus* species from South America.

Taking in account the arguments above, we propose the revalidation of *Tibelloides* Mello-Leitão, 1939 **gen. rev**. Besides the type-species, *Tibelloides* includes three other described species previously placed in *Cleocnemis*: *Tibelloides bryantae* (Gertsch, 1933) **comb. nov.**, *T. taquarae* (Keyserling, 1891) **comb. nov.**, and *T. reimoseri* **nom. nov.** (Table 2). The latter is a new name proposed for replacing *Apollophanes paraguensis* Gertsch, 1933, in honor of E. Reimoser (1864–1960, Austrian arachnologist who collected the type series of the species). This species was later transferred to *Cleocnemis* by Dondale & Redner [7], but it is herein considered as a *Tibelloides*. This generic placement creates a secondary homonymy as *Tibellus paraguensis* Simon, 1897 (herein considered a junior synonym of = *Tibelloides punctulatus*), has also been transferred to *Tibelloides* by us. Taking in account the articles 23.3.6 and 23.4 of the International Code of Zoological Nomenclature [40], which state that a junior synonym is available for priority and homonymy cases, we were compelled to propose a new name for Gertsch´s species. So, we propose *Tibelloides reimoseri* **nom. nov.** as a replacement name for *Apollophanes paraguensis* Gertsch, 1933.

***Tibelloides bryantae*** (Gertsch, 1933) comb. nov.

Figures 6 and 7

*Apollophanes bryanti* Gertsch, 1933: 14, figs. 22, 26.

*Cleocnemis bryanti* Dondale & Redner, 1975b: 1175.

*Cleocnemis rudolphi* Mello-Leitão, 1943b: 168 **syn. nov.**

**Type-material:** *Apollophanes bryanti*: Female holotype, PARAGUAY: **Asunción**: Asunción (MCZ, Reimoser Collection, photos). *Cleocnemis rudolphi*: Male holotype, BRAZIL: **Paraíba**: Campina Grande, R. von Ihering col. (MNRJ 41,991, examined, in loan).

**Additional material examined**: provided in Additional file 1.

**Diagnosis:** Females of *T. bryantae* have long and thin almost rectangular median septum, with long and straight darker lateral margins reaching from the rounded and flattened copulatory atria to epigastric furrow (Fig. 7c–e). Males have RTA elongated, with its tip appearing as a small, curved, black horn and VTA as a light rounded lobe covering the base of the black tip of RTA in ventral view, but RTA ending in relatively wide and excavated tip, with VTA appearing as a translucent semicircular lobe fitting in the RTA excavation in retrolateral view (Fig. 6f), embolus formed by a thin claw directed anteriorly forming a clearly slanted angle in relation to its transversal embolic base in ventral view (Fig. 6e), and appearing as a vertical needle in retrolateral view (Fig. 6f).

**Description. Male (Fig. ****6****) (UFRJ 1561).** Carapace longer than wide, wider between legs II and III, narrowed anteriorly; background color yellow, with wide pale longitudinal median band, bearing conspicuous V-shaped dark brown spot disposed from middle to anterior margin of carapace and with two parallel dark stripes placed anteriorly, reaching eye region, on its sides, at cephalic region, two conspicuous rows of three dark dots each, and near lateral margins of cephalic region, two brown lines displaced from anterior lateral eyes, passing aside posterior lateral eyes, and reaching middle of carapace. Two wide dark lateral bands, with many dark streaks. Numerous covering setae, especially at posterior margin of carapace (Fig. 6a, d). Clypeus vertical and yellow, with irregular dark spots, along with covering setae and a set of thin bristles particularly on its margins (only sockets left). Chelicerae with paturon yellow with irregular dark spots, with two teeth on promargin, cheliceral mound with a set of bristles, fangs brown (Fig. 6c). Labium wider than long, dark yellow, with rounded apex, slightly surpassing middle of endites. Endites longer than wide, yellow, with one depression on its inner margin near its base and another on its outer margin near its apex. Sternum with a slightly concave anterior margin and a narrow and rounded posterior margin, pale yellow, with few irregular dark spots, near its lateral margins (Fig. 6b).

Legs covered by different types of setae and with many long macrosetae disposed on femora, patellae, tibiae and metatarsi, with two ventral pairs on tibia and metatarsus I-II. Coxae yellow, femora, patellae, tibiae, metatarsi and tarsi pale yellow. Trochanters with set of erect bristles at distal margin of dorsolateral face. Tarsal claws unequal and pectinated; prolateral claw (mesal) with a row of 9–10 barely blunt teeth, very close to each other; and retrolateral claw (ectal) with only 4 teeth spaced out from each other. Claw tuft with abundant scopulated setae, and scopulae conspicuous on tarsi and metatarsi.

Abdomen oval, more than two times longer than wide, with a clear notch at middle, and covered with many long erect dark bristles (most only with sockets left). Dorsum mostly whitish, with a lot of visible guanine crystals, and two pairs of dark brown longitudinal bands, one pair disposed along lateral margins of dorsum, and another pair placed near margins of the cardiac mark, reaching all length of abdomen, but more visible from its middle to its posterior margin. Gray cardiac mark, reaching the middle of the abdomen, with a dark brown spot at the posterior tip. Laterals pale yellow, venter whitish grey with conic yellow spinnerets (Fig. 6a,b).

Measurements. Total length 5.28. Carapace 2.19 long, 1.73 wide, 0.71 high. Chelicerae 0.69 long, 0.38 wide. Clypeus 0.24 high. Labium 0.25 long, 0.33 wide. Endites 0.44 long, 0.42 wide. Sternum 1.18 long, 1.06 wide. Abdomen 3.22 long, 1.14 wide, 1.12 high. Leg: I. femur 3.08; patella 1.06; tibia 2.71; metatarsus 2.33; tarsus 1.33; total length 10.51; II. 3.75; 1.22; 2.40; 2.28; 1.72; 11.37; III. 2.62; 0.84; 1.86; 1.71; 0.78; 7.81; IV. 3.40; 0.98; 2.54; 2.51; 1.12; 10.55. Leg formula II > IV > I > III. Eye diameters and eye interdistances. AME 0.06, ALE 0.07, PME 0.06, PLE 0.08, AME–AME 0.18, AME–ALE 0.08, ALE–ALE 0.42, PME–PME 0.23, PME–PLE 0.30, PLE–PLE 0.82. MOQ 0.34 long in dorsal view, anterior width 0.28, posterior width 0.35.

Palpus (Fig. 6e,f) pale yellow, with dark brown spots at lateral faces. Tibia small, almost as longer as wide, with one large curved macroseta near retrolateral margin of basal half of its dorsal surface and other one longer at same region near prolateral margin, ventrally, one sinuous long macroseta in median region near its distal margin, and distinct set of abundant and erect thin bristles pointed distally and retrolaterally close to base of RTA. Tibial apophyses placed at ventral edge of distal margin, forming a robust stem divided into a distal globular pale yellow translucent lobe (VTA), and a basal dark claw shaped projection, pointed distally in ventral view (RTA apex). In lateral view, dark basal projection is almost as wide as base of RTA and presents a rounded base, and acute apex, it hides most of VTA, but its distal part remains visible. Cymbium oblong, with tip round and narrower than its middle, with a tuft of tenant hairs at edge of its tip; dorsally with two large macroseta at basal third near its prolateral margin, and two near its retrolateral margin. Ventrally, one large macroseta at basal third near prolateral margin of alveolus, and another at median, third near retrolateral margin of alveolus. Alveolus with a distinct set of small setae near tip of embolus. Tegulum discoid, dark yellow and with a distinct embolic base located at prolateral edge of apical margin, that forms a projected triangular mound and is followed by slight concavity at retrolateral region. MF occupying most of retrolateral half and extending to prolateral side of distal cymbial area just before EB. Conductor (C) membranous formed by a thin transparent veil covering from near distal margin of cymbium to base of embolic base (EB). Retrolateral marginal conductor (RMC) between C and the retrolateral margin of cymbium, rounded, thin and translucent. Embolus black, forming a relatively long and regularly curved claw, pointed ventroapically, and originating from retrolateral distal edge of EB. Sperm duct clearly visible through most of its extension, forming initially a large curved tube near retrolateral margin of cymbium, disappearing near basal margin of tegulum and reappearing near prolateral lower margin of tegulum as a sinuous S-shaped tube, wider at its long median loop and tapering in its distal portion, vanishing in black embolus.

**Description. Female (Fig. ****7****) (UFRJ 1560).** Color and structure usually as in male. Abdomen generally as in male, but proportionally wider, especially at its posterior half (Fig. 7a,b).

Measurements. Total length 5.98. Carapace 2.33 long, 1.84 wide, 0.76 high. Chelicerae 0.74 long, 0.43 wide. Clypeus 0.25 high. Labium 0.34 long, 0.38 wide. Endites 0.58 long, 0.43 wide. Sternum 1.18 long, 1.08 wide. Abdomen 3.64 long, 1.56 wide, 1.33 high. Leg: I. femur 2.85; patella 1.17; tibia 2.33; metatarsus 2.07; tarsus 1.14; total length 9.56; II. 3.26; 1.22; 3.02; 2.51; 1.43; 11.44; III. 2.40; 0.80; 1.70; 1.54; 0.86; 7.30; IV. 3.12; 0.92; 2.60; 2.18; 0.92; 9.74. Leg formula II > IV > I > III. Eye diameters and eye interdistances. AME 0.05, ALE 0.07, PME 0.05, PLE 0.08, AME–AME 0.17, AME–ALE 0.10, ALE–ALE 0.44, PME–PME 0.25, PME–PLE 0.32, PLE–PLE 0.86. MOQ 0.37 long in dorsal view, anterior width 0.29, posterior width 0.35.

Epigynum (Fig. 7c–e) longer than wide, with a long, thin and almost rectangular median septum, with long and straight darker lateral margins reaching from the rounded and flattened copulatory atria to epigastric furrow (Fig. 7c–e). Copulatory guides comma-like, very thin and placed over the anterior inner border of main spermathecae (S), curved around the rounded copulatory atria which bear the copulatory openings. Main spermathecae large, longer than wide, with inner margins almost touching at its anterior third and posterior third, being slightly concave at its median third. External margins sinuous, with grooves that forms round lobes at its anterior half. Posterior third projects dorsally as large tubes that taper to fertilization ducts. Glandular head placed anteriorly and directed externally, being small and round, surpassing anterior margin of S.

**Variation.** Length variation: 5.10 to 7.60 in females (n = 10) and 4.90 to 7.10 (n = 10) in males. Some specimens display a darker color pattern, especially on the lateral bands of carapace and sides of abdomen. Also, tibia I-II may bear three ventral macrosetae.

**Distribution.** This species is found from central Venezuela to Paraguay and also in all regions of Brazil.

**Taxonomic notes.** This species was erroneously described as *Apollophanes bryanti* Gertsch, 1933, but it should be amended to *Apollophanes bryantae*, as it was clearly a name in honor of Elizabeth Bryant, former arachnologist of Museum of Comparative Zoology (MCZ), following World Spider Catalog [1]. Although not cited in the original description, the homage was clear, as the holotype was from MCZ and Gertsch acknowledges the help from Bryant in relation to loans, drawings and information on the species in the introduction of the paper (see articles 31.1.2 and 32.5.1 in [40]). Its holotype is a female from Asunción, Paraguay (MCZ, photos examined), that presents the diagnostic characters of *Tibelloides* **gen. rev..** Mello-Leitão ([18], p. 168) described *Cleocnemis rudolphi* based on a male holotype from Campina Grande, Rio Grande do Norte state, Brazil (MNRJ examined). We paired both sexes based on overall similarity and several vials containing both males and females from throughout the distribution area of the species. Thus, we established the new synonymy *Cleocnemis rudolphi* Mello-Leitão, 1943 syn. nov. = *Tibelloides bryantae* (Gertsch, 1933) **comb. nov.**

***Tibelloides punctulatus*** (Taczanowski, 1872) comb. nov.

Figures 8 and 9

*Thanatus punctulatus* Taczanowski, 1872: 73.

*Tibellus punctulatus* Keyserling, 1880: 197, pl. 5, fig. 108.

*Tibellus punctulatus* Gertsch, 1933: 9, fig. 13.

*Cleocnemis punctulata* Caporiacco, 1955: 412.

*Tibellus paraguensis* Simon, 1897: 7 **syn. nov.**

*Tibelloides spatuliferus* Mello-Leitão, 1939: 76, figs. 60–62.

*Tibellus paraguensis* Mello-Leitão, 1945: 224 (synonymy of *T. spatuliferus*).

*Tibellus paraguensis* Achitte-Schmutzler & Rubio, 2016: 146, figs. 1a-f.

**Type-material**: *Tibelloides spatuliferus*: Female holotype, PARAGUAY, Dr. Ch. Ternetz Col. (NHMB, photos). *Thanatus punctulatus*: Male syntype, BRAZIL, **Amapá**, Uassa [not French Guiana, currently Uaçá in the state of Amapá], K. Jelski Col. (MIZ, photos); female syntype, FRENCH GUIANA: **Saint Laurent de Maroni**, K. Jelski Col. (MIZ, photos). *Tibellus paraguensis*: Female holotype, PARAGUAY: **Asunción** (MNHN, Simon Collection, not examined).

**Additional material examined**: provided in Additional file 1.

**Diagnosis**: Females of *T. punctulatus* have trapezoidal median septum, narrowed anteriorly near the deep elliptic copulatory atria and much wider posteriorly near the epigastric furrow, with posterior margin around twice as wide as anterior margin, and with its darker lateral margins forming a slightly concave arch. Spermathecae long, rounded, its anterior portion larger than posterior one (Fig. 9c–g). Males with RTA formed by a wide triangular base in retrolateral view, from which emerges a robust and short black horn, hiding most of VTA, which appears as a small pointed protuberance pressed against inner arch of RTA horn, in ventral view RTA appears as a more elongated lobe, with robust and slightly curved horn of variable width and length, bearing a VTA shaped as a triangular mound of variable length at the base of RTA horn, both RTA horn and VTA sometimes remembering pincers of unequal sizes, embolus formed by a long and curved spine-like tip raising straight up from its small triangular EB, but the large translucent conductor hides most of these structures in ventral view, with remaining embolus tip appearing as short black spine (Fig. 8e–g).

**Description. Male (Fig. ****8****) (UFRJ 1971).** Carapace longer than wide, wider at level of legs II, narrowed anteriorly; background color yellow, with many conspicuous dark dots, which become more numerous on margins and many dark brown erect bristles, which are often found on dark spots, especially at cephalic region, abundant covering setae (Fig. 8a). Clypeus vertical and yellow, with two rows of irregular dark spots, covering setae and a set of thin bristles particularly on its margins (most of them with only sockets left). Chelicerae with paturon yellow with irregular dark spots, with two teeth on promargin, fangs brown (Fig. 8c). Labium slightly wider than long, dark yellow, with rounded apex, reaching the middle of the endites. Endites slightly longer than wide, yellow, with one depression on inner margin near its base and another on its outer margin near apex. Sternum with a slightly concave anterior margin and a narrow and rounded posterior margin, pale yellow, with few irregular dark spots, near its margins (Fig. 8b).

Legs pale yellow, covered by different types of setae and with many long macrosetae disposed on femora, patellae, tibiae and metatarsi, with two ventral pairs on tibia and metatarsus I-II. Trochanters with a set of erect bristles at distal margin of dorsolateral face. Tarsal claws unequal and pectinated; prolateral claw (mesal) with a row of 9–10 barely blunt teeth, very close to each other; and retrolateral claw (ectal) with only 4 teeth spaced out from each other. Claw tuft with abundant setae, and scopulae conspicuous on tarsi and metatarsi.

Abdomen oval, more than three times longer than wide and with a clear notch at middle, and covered with many long erect dark bristles upon dark spots. Dorsum mostly whitish, with a lot of visible guanine crystals, and with two pairs of reddish brown sigillae. Gray cardiac mark, reaching middle of abdomen, with a dark brown spot at posterior tip and a horizontal stripe at middle conferring to it a cross shape. Laterals as dorsum, venter pale yellow and conic yellow spinnerets (Fig. 8a,b,d).

Measurements. Total length 5.15. Carapace 2.20 long, 1.91 wide, 0.85 high. Chelicerae 0.65 long, 0.37 wide. Clypeus 0.26 high. Labium 0.26 long, 0.35 wide. Endites 0.44 long, 0.41 wide. Sternum 1.17 long, 1.06 wide. Abdomen 3.08 long, 1.15 wide, 1.07 high. Leg: I. femur 3.28; patella 1.13; tibia 2.82; metatarsus 2.71; tarsus 1.36; total length 11.30; II. 4.24; 1.24; 3.79; 3.68; 1.81; 14.76; III. 2.56; 0.88; 2.00; 1.94; 0.93; 8.31; IV. 3.48; 0.96; 2.61; 2.69; 1.12; 10.86. Leg formula II > I > IV > III. Eye diameters and eye interdistances. AME 0.06, ALE 0.06, PME 0.06, PLE 0.08, AME–AME 0.13, AME–ALE 0.09, ALE–ALE 0.40, PME–PME 0.22, PME–PLE 0.27, PLE–PLE 0.78. MOQ 0.27 long in dorsal view, anterior width 0.24, posterior width 0.34.

Palpus (Fig. 8e–g) pale yellow, with dark brown spots at lateral faces. Tibia small, a little longer than wide, with one long and robust macroseta near prolateral margin of basal half of its dorsal surface. Tibial apophyses placed at ventral edge of distal margin, forming a robust bifurcated lobe shaped as a chela, distal tip is small and pale yellow (fusioned VTA) and basal tip is conspicuously claw shaped and dusky, pointed apically (RTA apex). Cymbium oblong, with tip round and narrower than its middle, with a tuft of tenant hairs at edge of its tip, a large macroseta at basal third of its prolateral face, another near retrolateral face, and two more displaced towards apical third of ventral face, closer to apical border of alveolus, smaller than others and directed apically. Alveolus with a distinct set of small setae near tip of embolus. Tegulum discoid and dark yellow. Embolus placed on a distinct embolic base located at prolateral edge of apical margin, that forms a projected roundish mound and is followed by a clear concavity at retrolateral region. MF occupying most of retrolateral half and extending to prolateral side of distal cymbial area just before EB. Conductor (C) formed by thin translucent lobe placed near distal prolateral margin of tegulum, and covering the EB. Retrolateral marginal conductor (RMC) placed between C and the retrolateral margin of cymbium, and formed by a rounded, thin and translucent veil. Embolus black, forming a relatively long and regularly curved claw, pointed ventroapically, and originating straight from retrolateral upper edge of the relatively small and somewhat triangular embolic base. Sperm duct clearly visible through most of its extension, forming initially a large curved tube near retrolateral margin of cymbium, disappearing near lower margin of tegulum and reappearing near prolateral lower margin of tegulum as a sinuous S-shaped tube, with a long median loop and its distal portion tapering and vanishing in black embolus.

**Description. Female (Fig. ****9****) (UFRJ 1972).** Color and structure usually as in male. Sternum totally pale yellow. Abdomen generally as in male, but wider, especially at its posterior half, and almost three times longer than wide.

Measurements. Total length 7.06. Carapace 2.60 long, 2.19 wide, 0.95 high. Chelicerae 0.85 long, 0.55 wide. Clypeus 0.35 high. Labium 0.35 long, 0.45 wide. Endites 0.64 long, 0.52 wide. Sternum 1.37 long, 1.31 wide. Abdomen 4.45 long, 2.24 wide, 1.51 high. Leg: I. femur 3.24; patella 1.14; tibia 2.87; metatarsus 2.44; tarsus 1.19; total length 10.88; II. 3.35; 1.26; 3.46; 2.97; 1.58; 12.62; III. 2.54; 0.87; 1.89; 1.72; 0.93; 7.95; IV. 3.31; 1.01; 2.26; 2.48; 1.00; 10.06. Leg formula II > I > IV > III. Eye diameters and eye interdistances. AME 0.05, ALE 0.07, PME 0.05, PLE 0.07, AME–AME 0.15, AME–ALE 0.10, ALE–ALE 0.45, PME–PME 0.29, PME–PLE 0.31, PLE–PLE 0.91. MOQ 0.26 long in dorsal view, anterior width 0.26, posterior width 0.39.

Epigynum (Fig. 9c–g) longer than wide, with trapezoidal median septum narrowed anteriorly near copulatory atria (CA) and much wider posteriorly near the epigastric furrow, with posterior margin around twice wider than anterior margin, and with long darker lateral margins forming a slightly concave arch (Fig. 9c–e). Copulatory guides as short conspicuous comma-like keels, at level of anterior third of spermathecae (S), tapering posteriorly and curving externally around deep elliptic CA which bear the copulatory openings. S large, reniform. anterior half larger than its posterior half, inner margin closer to each other anteriorly and gradually separating posteriorly, anterior margin with a median notch, dividing it into two lobes and posterior half wrinkled tapering posteriorly where it projects dorsally tapering to fertilization ducts (FD). Glandular heads aligned to anterior median notch of S, being small and piriform, surpassing anterior margin of S, and clearly visible at dorsal and ventral view (Fig. 9 e,f).

**Variation.** Length variation: 5.20 to 7.80 in females (n = 10) and 4.30 to 5.70 in males (n = 10).

**Distribution.** We examined numerous specimens from all regions of Brazil and also from Argentina and Paraguay. There are additional records in the literature for Venezuela ([21], p. 412) and Bolivia ([22], p. 146). The record from Peru ([42], p. 253) ([43], p. 9) s not considered here, as it is based on an immature female that was not examined by us.

**Taxonomic notes.** For details on the synonymies and other taxonomic decisions see above the taxonomic notes of the genus.

## Discussion

### Implications to higher-level Philodromidae systematics

Firstly, it is worth noting that neither the monophyly of Philodromidae, nor the phylogenetic status of Philodrominae and Thanatinae were main subjects of the present study. Nevertheless, some interesting preliminary results are discussed below.

The basic division of Philodromidae into two groups named Thanatini and Philodromini by Schick [6] and also recovered by Wheeler et al. [5] was obtained at least in part in our results. Conversely, Griotti et al. [8] recovered only Philodromini, with the Thanatini groups scattered at the basis of the tree. Following the rise of Philodrominae to family level by Homann [2], the use of subfamilies instead of tribes to name the two main groups of the family should be straightforward. However, Muster [3] did not apply any formal taxon category to the taxa recovered in his analysis, as his “Philodromini” was a paraphyletic assemblage containing a “Thanatini” distally inserted in his phylogeny. On the other hand, Wheeler et al. [5] and Griotti et al. [8] just referred to the old usage of tribe names by Schick [6]. We consider the two main branches of Philodromidae as two subfamilies, Philodrominae **new stat.** and Thanatinae **new stat.** as they were recovered in both analyses (Fig. 1). The position of *Titanebo* was already a point of disagreement between the preferred tree in the morphological analysis of Muster [3], where it was considered sister-group to Thanatini and other ill-placed “Philodromini” taxa, and the molecular analyses of Wheeler et al. [5], where it was recovered as sister group to remaining Philodromini, and the molecular and morphological analysis of Griotti et al. [8], where a non-monophyletic *Titanebo* was placed within Philodromini. Our analyses indicate another possibility, with *Titanebo* as sister-group to both subfamilies, as already recovered in the implied weighted analysis of Muster [3]. However, any decision on the correct placement of *Titanebo* and precise limits of Philodromidae subfamilies should await new analyses, including a larger taxonomic coverage and additional molecular markers.

Philodrominae as recovered by our analyses (Fig. 1) includes *Philodromus*, *Gephyrellula*, *Pagiopalus*, *Petrichus*, *Pedinopistha*, and “*Cleocnemis*” *mutilata*. Despite Philodrominae not being recovered with significant clade support (SH-aLRT/UFBoot = 80.3/85, PP = 0.76), it is interesting to note that two of the diagnostic features given by Schick [6] for Philodromini are clearly recognizable in “*Cleocnemis*” *mutilata*: tegular suture (TS) and ventral bulbar apophysis (VBA), which is represented by a massive tegular scutum.

Furthermore, our results suggest that Thanatinae should also be expanded to include *Cleocnemis *sensu stricto, *Fageia*, and *Tibelloides* **gen. rev.**, besides the formerly included genera *Apollophanes*, *Thanatus*, and *Tibellus*, resulting in a much larger clade than considered before. Some of the characters of Thanatinae given by Schick [6] are observed in these genera, e.g. posterior eye row typically strongly recurved, PME typically more closely approximated than in the Philodrominae, interdistance index usually less than 1.2; male palpus without paraconductor bulbar apophysis, tegular suture absent, and large membranous conductor. However, internal relationships among Thanatinae genera are not well-resolved and would benefit from adding representatives of other lineages of the already studied genera.

### Identity and phylogenetic status of *Cleocnemis *sensu stricto

When *Cleocnemis* was proposed, Simon [13] provided an abbreviated description of the genus and a longer description of an adult male and an immature female of *C. heteropoda*, both from Tijuca, Rio de Janeiro State. The type-material should have been in MNHN (Paris, France), but it is considered lost. A careful examination of the MNHN collection was made by our collaborator (Pedro Castanheira, pers. comm.), but no trace of syntypes or any other specimen identified as *C. heteropoda* by Simon was found. The cardfile for this species in MNHN was written by Mello-Leitão, judging by his characteristic handwriting, and includes records of four different samples attributed to *C. heteropoda*, namely one from Paraguay (juvenile, MNHN9041) and three from Brazil: Bahia: Santo Antônio da Barra (now Condeúba, two males, one female, MNHN11501), Pará: Santarém (one male, MNHN 16,078), and Ceará: Serra de Baturité (one male, MNHN16078). Except of the latter specimen, we were able to find the other three lots at MNHN, all identified and with labels written by Mello-Leitão. Samples from Paraguay and Bahia referred as *C. heteropoda,* actually belong to specimens that can be placed in Group IV (which includes “*Cleocnemis lanceolata*”). The only sample that matches the original description of Simon and specimens collected at Tijuca is the male putatively from Santarém. However, this locality is probably wrong, as all examined specimens of *C. heteropoda* come from southeastern and southern Brazil (see 15 section).

In the genus description, Simon [13] considered *Cleocnemis* similar to *Thanatus* C. L. Koch, 1837, emphasizing eye arrangement and leg proportion. As Simon stated, posterior eyes are in a more curved line, middle eyes evidently smaller than lateral ones and more distant from the lateral than from each other. Median eye area a little longer than wide and a little shorter anteriorly than posteriorly. In *C. heteropoda* description, Simon [13] gave additional and important details on morphology and color pattern of the type species: F [immature] 3,5 mm long, cephalothorax anteriorly low, shortened and truncated, a little depressed in the middle, pale brown, infuscated and reticulated with black pigment at the sides, pars cephalica bearing at its posterior part a dark stripe with two branches. Clypeus flat, vertical, clearly shorter than median eyes area. Abdomen wide, oblong, flattened, truncated and notched at anterior margin, a little wider behind, dorsum with black spots irregularly spread at the margins and a brownish median stripe narrowing posteriorly, venter grayish brown, covered with whitish plumose setae. Sternum pale brown with blackish margins. Legs pale brown, robust and short, with many macrosetae and setae. Legs I and II with many black dots irregularly placed, leg III with more black dots and patella and tibia almost black, leg IV with less dots. Palpi pale brown with some black pigment. Vulva not entirely developed. M 3,5 mm long. Cephalothorax wider [than in female], median stripe less contrasting and reddish brown, thinner and not so marked posteriorly, covered with wide and long setae, plumose or not, of yellowish hue. Legs longer, darker and with reddish brown and black dots, leg III with patella, tibia and metatarsus almost entirely black. Palpi short and robust, femur short, with 1–2 macrosetae at apical part, patella subquadrate, tibia short and thin, its apical margin bearing, at the ventral side, a small RTA, which is oblique, flattened and truncated at its apex, tarsus wide, oval and a little pointed, bulb long and simple, with embolus short and black at its apex, directed outwards and free at most of its extension.

Simon [13] also cited legs short and robust, very spiny and bristly, with legs 1, 2 and 3 subequal and leg IV only slightly smaller and thinner than others and tarsi more or less distinctly scopulated. Nine years later, Simon [14] redescribed the genus in similar words, but omitted some characters, like the proportion of the legs, and added that the anterior lateral eyes separated by the same distance from the anterior median eyes and the posterior median eyes.

Considering all these features, as well as material examined from the type locality of *C. heteropoda*, we have established the identity of the species. As a result of many collecting trips to Tijuca and examination of available material in scientific collections, we were able to find only one Philodromidae species in that locality. This relatively common species agrees well with the description Simon [13] gave for *Cleocnemis heteropoda*, in eye arrangement, leg proportion, color pattern, and conformation of retrolateral tibial apophysis of the palpus, thus allowing a precise identification. Moreover, this species was the only one matching Simon’s [13] description that we were able to find in Rio de Janeiro city. Taking into account the disappearance of the type material of *C. heteropoda* and the fact that it is the type species of *Cleocnemis*, whose identity and composition was not clear, the need for a neotype designation is fully justified. Besides the phylogenetic placement of the species (Fig. 2), we redescribed and designated a neotype in the taxonomy section of this paper (Figs. 3 and 4a,b).

Misconceptions of the identity of *Cleocnemis* began with Simon [14], who mentioned that four species from South America described as *Thanatus* by Keyserling should be placed in *Cleocnemis*, without giving their names. He was probably citing *T. chorillensis* Keyserling, 1880 (Peru), *T. granadensis* Keyserling, 1880 (Colombia), *T. maculatus* Keyserling, 1880 (Peru), and *T. taquarae* Keyserling, 1891 (southern Brazil), as they were the only species described by Keyserling as *Thanatus* from South America at that time [50, 51]. Among these, the only species validly placed in the genus is *Cleocnemis taquarae* (Keyserling, 1891), transferred by Mello-Leitão [15]. The other three species remain in *Thanatus*, although their identity and placement are dubious, as there is no other paper dealing with them besides the original description.

The current heterogeneous concept of *Cleocnemis* was developed mainly by Mello-Leitão [15], who described and transferred most species to the genus in his revision of Brazilian Philodromidae. He probably followed the hint by Simon [14] about *C. taquarae*, but not about the other three species. Species he considered as *Cleocnemis* in his 1929 paper [15] fall in five different informal species groups, four of which are dealt in our morphological analysis (see 11 in 9 section), with distinct somatic and genitalic characters: Group I, including *C. heteropoda,* the type-species of *Cleocnemis*; Group II, including *C. taquarae*; Group IV, including *C. lanceolata*; and Group V, including *C. mutilata*, *C. serrana* and *C. xenotypa*. Besides, he also placed *Philodromus meridionalis* Keyserling, 1891 in *Cleocnemis*, but it was already transferred correctly to *Petrichus* by Dondale & Redner [16]. Also, Mello-Leitão [15] redescribed *Gephyrina imbecilla* and described two new genera *Berlandiella* and *Fageia*, which are all relevant to *Cleocnemis* delimitation. Following our morphological analyses, *Gephyrina imbecilla* belongs to *C. mutilata* group (Group V). Later, Mello-Leitão [17, 19] described two additional *Cleocnemis* species which actually belong to *Fageia*, *Cleocnemis moschata* and *Cleocnemis rosea*, thus adding a sixth distinct species group (Group III) to his widened concept of *Cleocnemis*. In our analyses, we worked with specimens of all species groups loosely considered as *Cleocnemis* by Mello-Leitão, including *Petrichus*, a very distinct genus of southern South American Philodromidae recently revised by Griotti et al. [8].

The dubious identity of *Cleocnemis* and its type-species probably contributed a lot to the messy taxonomic history of the genus and also of several other Neotropical Philodromidae. The present molecular phylogeny resulted in six different lineages with representatives of eight from the 14 species previously placed in *Cleocnemis* (Fig. 1). The association of the results of molecular phylogeny analyses with morphological comparisons made it possible to split *Cleocnemis* into five recognizable species groups, from which three could be allocated herein in previsouly described genera and two are putative new genera. Explanations for each of these groups are provided below, along with a discussion about conflicts between morphological and molecular analyses. A summary of our taxonomic conclusions is given above (Table 2).

Our molecular phylogenetic analyses recovered *Cleocnemis *sensu stricto as a monophyletic group distinct from other species previously placed in *Cleocnemis*, with strong to maximum support (Fig. 1, SH-aLRT/UFBoot = 97.8/97, PP = 1.00). Nevertheless, species representing *Cleocnemis *sensu stricto were recovered as a clade only in COI gene tree, but without significant support (PP = 0.57), and in 28S tree, but represented by only two terminals (see Additional file 4). The poor resolution of gene trees strengthen the critical role of multiloci analyses in resolving phylogenetic relationships of the group.

### Taxonomic status of the remaining *Cleocnemis *sensu lato

#### *Tibelloides* Mello-Leitão, 1939 gen. rev.

Despite our present proposal of revalidating *Tibelloides* to harbor the species from the Group II (species in red in Fig. 1), our phylogenetic results do not provide support for this decision. In our analyses, we did find strong support (SH-aLRT/UFBoot = 100/100, PP = 1.00) for a clade including most species of the morphologically defined *Tibelloides*: *Tibelloides bryantae* **comb. nov.**, *Tibelloides reimoseri* **nom. nov.** and *Tibelloides taquarae* **comb. nov.**, besides two undescribed species. However, this clade was not recovered in any of the multiloci analyses as sister-group of the type species, *Tibelloides punctulatus*, which was grouped with typical *Tibellus* instead*.* Nonetheless, COI gene tree (Additional file 4c) supported the monophyly of *Tibelloides*, as presently defined (except of *T. reimoseri*, which was not sampled), without significant support (PP = 0.74). The close association of *Tibelloides punctulatus* with the two species of *Tibellus* had low to no significant support (SH-aLRT/UFBoot = 88.8/ < 50, PP = 0.55). So, taking into consideration the weak support for the last grouping and the many somatic and genitalic characters that *Tibelloides punctulatus* shares with the other species of Group II, we decided to treat them all in *Tibelloides* for now, instead of erecting a new taxon without diagnostic morphological characters. Future phylogenetic analyses will hopefully add new taxa, molecular markers, and morphological characters to try to further resolve the monophyly of *Tibelloides*.

#### *Fageia* Mello-Leitão, 1929

Species of the Group III of *Cleocnemis *sensu lato are *Cleocnemis moschata* Mello-Leitão, 1943 (species in green in Fig. 1), from Rio Grande do Sul, Brazil, and *Cleocnemis rosea* Mello-Leitão, 1944, from La Plata, Argentina. The former is known only from the original description (holotype lost in MNRJ fire), but we examined many additional specimens from Southeastern and Southern Brazil and also from Paraguay. The juvenile holotype of *Cleocnemis rosea*, deposited at Museo de La Plata was examined. Taking into account photographs, original drawings, original descriptions, and additional specimens from those species, it was possible to state that they are clearly related to *Fageia amabilis* Mello-Leitão, 1929, the type-species of *Fageia* Mello-Leitão, 1929, from Bahia State, Northeastern Brazil. All three species have a wide and flattened carapace, a typical large, flat, and pentagonal abdomen, with conspicuous spatulated setae, along with congruent leg proportion and color pattern. Hence, the two species placed in *Cleocnemis* are herein transferred to and referred to as *Fageia moschata* (Mello-Leitão, 1943) **comb. nov.** and *Fageia rosea* (Mello-Leitão, 1944) **comb. nov.**

The phylogenetic position of *Fageia* was incongruent in the different analyses, while it was placed as sister-group of *Thanatus* in the ML with no significant (SH-aLRT/UFBoot = 76.8/ < 50, PP = 0.69), it was recovered in a polytomy with all other Thanatinae in the BI.***.***

#### Group IV

*Cleocnemis lanceolata* (species in yellow in Fig. 1) species belongs to another species group of *Cleocnemis *sensu lato, the Group IV. It includes spiders with short carapace and abdomen, subequal legs, and a conspicuous trifurcated black stain dorsally on abdomen, usually collected on the ground or over grasses in open woods and grasslands. The holotype of *C. lanceolata* is a female, from Mato Grosso, Brazil, but additional specimens were found from Southern Brazil and Paraguay. Through analyses of photographs of the type-material and additional specimens of both sexes, we concluded that this species cannot be placed in any of the described genera of Philodromidae, due to lack of information and specimens. Thus, this species is referred to as “*Cleocnemis*” *lanceolata* and considered as “*incertae sedis*”.

Another species belonging to the Group IV is *Paracleocnemis apostoli*, based in a male from Corrientes, Argentina, which is closely related to “*Cleocnemis*” *lanceolata*, based on the darker body, rigid setae on carapace and abdomen and large RTA with tip projected ventrally. It is clearly not congeneric with the enigmatic *Paracleocnemis termalis* Schiapelli & Gerschman, 1942, also from Argentina. Based only on female characters, *Paracleocnemis* may be set apart from Group IV by its paler body, without rigid setae on abdomen, epigynum with MS not reaching the epigastric furrow and restricted to the anterior half of the epigynal field, and with a large posterior GP on each side delimiting a deep concavity close to the epigastric furrow, and median-sized, oblong main spermathecae, with clearly defined basal lobe. So, this species is referred to as “*Paracleocnemis” apostoli* and considered as “*incertae sedis*”.

Group IV probably represents an undescribed genus related to *Paracleocnemis* and *Apollophanes*, but we decided to not formally describe it without additional morphological and molecular information on those genera. In our phylogenetic analysis, Group IV, represented only by “*Cleocnemis*” *lanceolata*, was recovered as sister-group to *Apollophanes*, but with low support (SH-aLRT/UFBoot = 89.6/77, PP = 0.94).

#### Group V

*Cleocnemis mutilata* (species in purple in Fig. 1), from Rio de Janeiro City, and *Cleocnemis serrana* and *Cleocnemis xenotypa*, both from Petrópolis (Rio de Janeiro, Brazil), belong to Group V of *Cleocnemis *sensu lato. They are all known only based on their descriptions and illustrations (types destroyed at the MNRJ fire at 2018). The first described species from Group V was *Gephyrina imbecilla* Mello-Leitão, 1917, based on an immature female from Rio de Janeiro city (type also destroyed at MNRJ fire). We collected many specimens belonging to only one species of Group V from Rio de Janeiro City, which is widespread in Southeastern Brazil. Following the first reviewer principle (article 24.2 in [40]), we herein consider *Cleocnemis mutilata* a senior synonym to *Gephyrina imbecilla* Mello-Leitão, 1917 **syn. nov.** The original descriptions of each of those species, published in the same paper, are very similar. *Cleocnemis mutilata* was based on a female and its poor original illustration ([52], fig. 11) clearly depicts the epigynum of the only species we collected in Rio de Janeiro, what led us to keep *C. mutilata* as the valid name for this species. On the other hand, the original description of *Gephyrina imbecilla* is not accompanied by illustrations and does not mention an epigyne, which indicates that it was probably based on an immature specimen. Moreover, Mello-Leitão ([15], fig. 32) included a good illustration of the holotype of *G. imbecilla*, which depicts a pale juvenile specimen with typical color pattern of the species. *C. mutilata* may also be a senior synonym of *Cleocnemis serrana* and *Cleocnemis xenotypa*, but a sure conclusion awaits collection of new specimens from Serra dos Órgãos.

So, Group V includes “*Cleocnemis” mutilata* (Mello-Leitão, 1917) (senior synonym of *Gephyrina imbecilla* Mello-Leitão, 1917 **syn. nov.**), *“Cleocnemis” xenotypa* Mello-Leitão, 1929, and *“Cleocnemis” serrana* Mello-Leitão, 1929, all from Southeastern Brazil. This group comprises flattened laterigrade spiders, with unequal legs, bearing a second pair clearly longer than the others. Females have very simple genitalia, with two small reniform spermathecae, while the males have very distinct palpus, with large VBA forming prominent tegular scutum, very large sinuous PCA, absence of membranous conductor, unique TC with distal furrow and keels to harbor embolus tip and relatively long tubular embolus entirely placed at prolateral face of tegulum. Many specimens were caught on the foliage of bushes and small trees in open woods and orchards.

This species group seems to be distinct from all other Philodromidae genera, judging by their palpus and epigynal morphology, but is not formally described by now pending new morphological and molecular data. The presence of tegular suture and large PCA and VBA indicates that it is a representative of the Philodrominae (see [3, 6]), a taxonomic placement also supported by our phylogenetic analyses that found the species as weakly related to *Philodromus cespitum* + *Philodromus aureolus* (SH-aLRT/UFBoot = 84.6/86, PP = 0.51).

### Other genera associated with *Cleocnemis *sensu lato

Two other Philodromidae genera have been compared to *Cleocnemis* in the literature, *Procleocnemis* and *Paracleocnemis*, but they do not seem to be related to *Cleocnemis *sensu stricto or to any other of the four genera containing species formerly placed in *Cleocnemis*.

*Procleocnemis* was only cited by Mello-Leitão in its original description ([15], p. 111–112). The holotype of its type-species, *Procleocnemis concolor* Mello-Leitão, 1929, was a female from Petrópolis, Rio de Janeiro State, Brazil, but it was lost even before the tragic 2018 fire at MNRJ. Its position is uncertain, but the good original illustration of the habitus of the female holotype ([15], fig. 34) indicates that it is not a Philodromidae. It is transferred herein to Thomisidae, and should be compared to *Tmarus* Simon, 1875 or other Misumeninae genus, judging by the wide carapace, lateral eyes clearly with a whitish covering around their bases, two anterior pair of legs much longer than posterior ones, and pentagonal elevated abdomen “remembering a *Misumena*” after Mello-Leitão’s words.

*Paracleocnemis* was erected to accommodate *P. termalis*, based on a female holotype from Santiago del Estero, Argentina (type examined). A second species described was *P. apostoli*, based on a male from Corrientes, Argentina. The type-species has a very different epigynal shape, with a large unpaired atrium covering most of the epigynal area, and a large-sized body, especially the abdomen, that are enough to separate it from all other Neotropical Philodromidae genera. The second species, “*Paracleocnemis'' apostoli,* belongs to Group IV (see above). *Paracleocnemis* may be related to *Apollophanes, Thanatus*, and Group IV, but additional material, in particular an actual male of the type-species, is needed to evaluate its position in relation to other Philodromidae.

### Notes on genitalic morphology of Philodromidae

There is a large and confusing variety of names applied to different structures of male and female genitalia of Philodromidae (ex. [3, 6, 16]). Many names are used in a different sense from those commonly applied to spider genitalia in other families (ex. [26, 53–61]). Below we discuss some of those names and give our reasons to choose among them.

The genital bulb represents the male secondary genitalia and is formed by a modification of the last article of pedipalps. It defines a large array of different structures that evolved independently in many spider taxa [6, 53, 59]. Several structures of the bulb and other parts of the pedipalp are responsible to guide the intromittent piece of the bulb, called embolus (E), into the copulatory opening of the female. For example, in Philodromidae and many other Araneomorphae families of the so-called RTA clade [5], the pedipalp tibia develops an apophysis called the retrolateral tibial apophysis (RTA) that also helps to position the embolus during the coupling process. In many Philodromidae species, there is an additional apophysis on the tibia, the ventral tibial apophysis (VTA). When both apophyses are present, VTA may be separate from RTA or be closely pressed or partially fused to the RTA base [6]. At least two additional apophyses may appear in unrelated groups of Philodromidae. In the nominal *Philodromus* subgenus, there is an additional ventral apophysis placed mesally at its distal margin, the Mesal Ventral Tibial Apophysis (mVTA) ([62], pl. 11; [63], pl. 3e). Another *Philodromus* subgenus with an additional tibial apophysis is *Artanes*, which presents an apophysis at the dorsal corner of the distal margin of the tibia in retrolateral view, the Dorsal Tibial Apophysis (DTA) ([3], figs. 14, 15).

The genital bulb is a rounded or flattened structure placed inside a cavity of the pedipalp tarsus, the alveolus, while surrounding portions of the tarsus are called the cymbium. The bulb itself is usually divided in three portions: subtegulum, tegulum, and embolic (or apical) division. Inside the bulb, we find the receptaculum seminis, a hollow tube that holds sperm after the induction process, which is usually divided in three parts, a basal collapsible fundus at the subtegulum, a median and longer reservoir at the tegulum, and a terminal ejaculatory duct traversing the embolic division [6, 53, 59]. In Philodromidae, the subtegulum is hidden by the tegulum, which joins the embolic division without a membranous hematodocha between them. The tegulum bears several different structures in Philodromidae, but lacks a median apophysis [6].

A conductor is the most widespread of tegular structures in the family, and functions as support to the embolus. Structures named as conductor in Philodromidae may not be homologous, as they differ substantially in shape, constitution, and position [3]. The most common structure is the translucid, soft conductor placed at the distal retrolateral area of tegulum, but not occupying the margin itself, as in most Thanatinae (Figs. 2d,e, 6d,e and 8e–g; [64], figs. 9–16) and in several Philodrominae ([65], figs. 1–3, 7–8; [63], pls. 3 g,i, 6a–c). This soft conductor may be called just conductor (C) as we assume it may be the primitive type of conductor, judging by its widespread occurrence in many genera of Cheiracanthiidae and Miturgidae (see [66] and [4]). The soft C may be formed by just a membranous lobe (Figs. 2d,e, 6d,e and 8d,e; [64], figs. 1–3, 7–8) or by a membranous veil ([46], figs. 135,137; [63], pls. 3i, 6a–c), which is sometimes very elongated and extended over the prolateral area of tegulum ([63], pl. 3e, h). The texture of the soft C may vary even inside one genus, as in *Tibelloides, Tibellus,* or *Philodromus*, where some species have a more fleshy (Fig. 6d,e; [62], pl. 11) or even a very rigid structure ([67], figs. 7–10). In many groups, the retrolateral distal margin of the tegulum is modified to serve as a guide for the tip of the embolus, named as retrolateral marginal conductor (RMC). This structure is usually named just as conductor and is found, for example, in *Titanebo* ([68], figs. 30, 36–38), *Apollophanes* ([7], figs. 7–10), or *Gephyrellula* ([10], figs. 10–12), forming a thin lamella near the cymbium. In *Tibelloides* and *Cleocnemis*, both the soft C and the RMC seem to occur side by side, with a more transverse, pointed and smaller median portion representing the soft C, and a larger keel-like, elongated retrolateral portion, the RMC (Figs. 2d,e, 6d,e and 8e,f,g). In some groups of Philodromidae, the conductor seems to be a complex structure formed by the soft C and modifications of most of the distal part of the tegulum, as in the large conductor of some species groups of *Philodromus* ([7], figs. 1, 3, 7, 9; [46], figs. 206–207; [3], figs. 10–13; [69], figs. 186–187), which may also include secondary sclerites as the conductor process (CoP) ([3], figs. 10–13).

Other special tegular structures found in several different groups of Philodromidae are dealt with below. Following Schick [6], the paraconductor bulbar apophysis (PCA) or paraconductor for short is a usually strongly produced, elongated, and heavily sclerotized apophysis originating from a distal membranous field (MF) between the retrolateral edge of the conductor and the retrolateral margin of tegulum ([6], fig. 81; [46], figs. 188–191; [65], figs. 4–6), and it is also called retinaculum [3]. However, the shape and degree of sclerotization of the PCA may be variable intraspecifically ([65], figs. 4–8, 10, 11). PCA should not be confused with the RMC, as the latter is not sclerotized and originates at the retrolateral border of the tegulum, while the former usually originates from the center of MF. Schick [6] also recognized a ventral bulbar apophysis (VBA) as a projecting or emarginate structure placed at the distal region of tegulum near the apex of the middle loop of the reservoir. In Thanatinae, it is represented by a short, distally projecting and truncate structure ([6], figs. 116, 121, 127), called tegular apophysis by Logunov [70] and Kastrygina & Kovblyuk [64], while in Philodrominae it appears as a lobe associated to the PCA ([6], figs. 36, 45, 46) that is sometimes enlarged and contains the whole of PCA and MF ([71], figs. 1–2, 4–5; [63], pl. 3 h), an elongated, arcuate plate below the veil C ([62], pl. 11; [63], pl. 3e), or a very large shield-like structure as in “*Cleocnemis*” *mutilata*. In most Philodrominae ([6], figs. 3, 14, 24; [65], figs. 8, 11) and also in some genera like *Pagiopalus* ([72], figs. 103, 108, 113, 118) and *Titanebo* ([68], figs. 24, 30, 38), there is a characteristic tegular suture (TS), an elongated furrow between the arms of the middle loop of the reservoir.

The embolic division of Philodromidae may be clearly delimited from the tegulum ([73], figs. 1a–b, 2a–b) or appear as a more sclerotized terminal lobe of it (Figs. 2d,e, 6d,e and 8e,f). It is formed by a basal portion usually elongated, called the embolic base (EB), and a terminal embolus (E) which is very rigid and usually black. The E may be very variable, usually appearing as a short straight spine ([7], figs. 7–9; [74], figs. 32–33) or curved claw (Figs. 2d,e, 6d,e and 8e–g; [7], figs. 8, 10), a moderately elongated and curved prong ([25], figs. 1, 17, 29, 33), or even a very long or filiform curved structure ([6], fig. 116; [10], figs. 10–12, 19–21; [63], pls. 3i, 6a–c), sometimes covering most of the perimeter of the bulb ([69], figs. 186, 187; [63], pl. 3d). The EB and E usually originate from the distal prolateral region of the tegulum (Figs. 2d,e, 6d,e and 8e–g; [6], fig. 116; [46], figs. 76, 81; [10], figs. 10–12, 19–21), but its position may vary from the basal prolateral ([67], figs. 1, 3; [63], pl. 3d, h) to the distal ([63], pls. 3i, 6a–c) or even basal ([46], fig. 206) retrolateral regions of that structure.

In general usage, the female genitalia of typical entelegyne taxa have a circuit arrangement, with two different kinds of openings to the outside: the primary gonopore, hidden within the epigastric furrow and functioning as the laying opening for eggs, and copulatory openings (CO), serving as the entrance way for sperm. The CO are formed through the invagination of the primary epigynal folds, which form a series of surface structures with variable degrees of sclerotization, the epigynum, with a protective and guiding function. The epigynal fold invaginations give origin to all the chitinized structures of the fully-grown vulva and connect it to the *uterus externus* [60]. After the CO, there is a copulatory duct (CD), a tube or folded channel of variable length and sclerotization, which is crossed by the male embolus during copulation and leads to some kind of chitinized sperm receptacula, which is usually represented by a pair of spermathecae. Most entelegyne taxa present a division of the spermathecae in a larger and round base (BS) and a smaller glandular head (GH), usually connected by a thinner glandular head stalk (GS) of variable size and length. The CD may be connected directly to BS or join the GS instead. In the latter example, the male embolus may cross not only the CD, but part of the GS before depositing sperm in the BS ([75], fig. 2). During the fertilization process, sperm is conducted from BS through the fertilization ducts (FD) up to an inner area of the *uterus externus* where eggs are fertilized. After fertilization, eggs are laid through the gonopores [75–77].

We prefer to use CO (sensu Dondale & Redner, 1976 [16]) instead of intromittent orifices (sensu Schick, 1965 [6] and Muster, 2009a [3]) as it is a designation widely used outside Philodromidae. CD (same as copulatory tube sensu Dondale & Redner, 1976 [16]) is preferred over *bursa copulatrix* (sensu Schick, 1965 [6]), as it is also widely employed. Besides, *bursa copulatrix* is usually applied instead to a membranous cavity following the CO and connected to the *uterus externus*, which is found in many Mygalomorphae and haplogyne spiders, but also to a membranous pocket present in some entelegyne spiders [58, 75–77]. Muster [3] used *bursa copulatrix* in a particular sense, referring only to the “canal or three-dimensional region […] of the vulva that is passed by the embolus during copulation before it enters the receptacula and that is NOT connected with the duct leading to the glandular heads”. He also added that the *bursa copulatrix* is usually “less strongly sclerotised than the receptacula”. Muster reserved the designation copulatory duct to the situation when “the intromittent canal is merged with the ducts of the glandular heads”, forming a structure which is “often strongly sclerotised”. Muster’s usage is not in accordance with the prevailing terms employed to describe female genitalia in spiders in general and also does not adequately describe the whole amount of variation we found.

As in spiders in general, the association of the CD and the glandular head stalk (GS) varies widely in Philodromidae (see [75] for example). In many species, it is difficult to determine the exact position of CO, which makes it hard to assess the size of the CD and its relation to GS. They may be completely separated, as in some *Philodromus* species without GS and with GH partially fused to BS ([3], fig. 21b), but are usually fused during at least some part of their trajectory. The GS seems to connect the GH directly to the CO region without a distinctly separated CD in species from genera belonging to different lineages, as, for example, *Pedinopistha* Karsch, 1880 ([72], fig. 135,140), *Apollophanes* ([7], figs. 29, 34), *Tibellus* ([78], figs. 2077, 2081), *Ebo* ([46], figs. 75, 78), *Gephyrellula* ([10], figs. 22, 23), *Rhysodromus* ([65], figs. 19, 20), and many *Philodromus* ([67], figs. 48, 56, 70). Most of those species bear thin and not well sclerotized GH, as in *Pedinopistha* Karsch, 1880 ([72], figs. 135, 140), *Tibellus* ([78], fig. 2081) or some *Philodromus* ([67], figs. 67, 71). A distinct CD of variable length is also found, forming a complex with a long fused CD + GS region in many *Titanebo* ([68], figs. 27, 35, 39–41, [25], fig. 5) and very elongated and twisted around BS with a small CD + GS near GH in *Philodromus imbecillus* group ([46], figs. 211–213, 224–226). In *Philodromus*, we found the largest amount of variation regarding inner genital morphology. Some species display a robust and more sclerotized GH and an indistinct CD ([67], figs. 45, 50, 55). Others present a small GH followed by a long CD + GS ([71], figs. 8, 10, [63], pl. 5e), sometimes quite elongated ([63], pl. 5b, d). Sometimes the area around the CO is placed inside large and complex folds of the epigynum, making it difficult to determine its location and CD/GS association, as in many *Philodromus* species ([67], figs. 76; [46], figs. 107, 157–158, 182–183, 196–198, 279, 287–288).

The several structures of epigynum also received many different names in literature. The epigynum is placed at the area surrounding the CO, just before the epigastric furrow (or genital groove), and is usually formed by several different plates and other structures with variable degree of sclerotization. We follow Schick [6] and Dondale & Redner [46] in using median septum (MS) to designate any median sclerotized plate of the epigynum, which may cover most or the entire length of the median section of the epigynal area (Fig. 3d, e; [79], figs. 163, 210; [7], figs. 26, 34), or be restricted to the anterior (as in *Paracleocnemis termalis*, photo of type examined) or posterior section of it ([46], fig. 97; [62], pl. 11; [63], pl. 4a; [11], fig. 4). MS is usually flat or regularly curved, but may also be excavated at its mesal area ([3], fig. 25a; [80], figs. 9, 11, 15). In some species of *Pulchellodromus* and *Philodromus*, there is a median keel placed over the MS, usually extending only through its anterior portion ([81], figs. 32–34; [63], pl. 4b) or all along its middle line. The inner lateral margin of the epigynal folds (or spermathecal apodemes) are called epigynal suture after Schick [6], demarcating the MS from the lateral areas.

The lateral areas around MS are poorly sclerotized and flat in many genera (ex. [72], figs. 125, 130; [7], figs. 29, 34; [46], figs. 75, 78; [10], figs. 13, 22). Sometimes there are distinct lateral plates (LP), with varied degrees of sclerotization (Figs. 3d,e; [25], figs. 19, 35). Those LP may be clearly more elevated than the median area, which may have a central furrow, as in several *Halodromus* ([25], fig. 35) and species of *Philodromus aureolus* group ([16], fig. 196; [62], pl. 11).

Albeit somewhat confusing, most cavities or pits on the epigynal area are called atria in the literature, despite the word etymology implies a cavity placed at the entrance of a larger structure, as the CO itself is the opening for the inner sclerotized genitalia. Usually, each CO is placed below a copulatory guide (CG) (name preferred instead of guide pocket sensu Schick, 1965 [6]), which serves as a guiding structure for the male embolus [3]. The CG is represented by a hood or keel, often associated with each anterior arch of MS (Figs. 7c,d and 9c–e; [67], figs. 37, 39, 46), but sometimes placed more posteriorly on the MS ([46], figs. 139–140, 210) or on the rims of the somewhat elevated LP ([7], fig. 29, 34; [16], fig. 106; [46], figs. 192–195; [65], fig. 19). Just around the CO, each CG usually partially delimit a small copulatory atrium (CA), which may be associated to one single or a pair of larger cavities. Following Schick [6], the single cavity that covers the anterior mesal portion of the epigynum just before a relatively short and posteriorly placed MS is called mesal atrium or simply atrium (AT) (ex. [62], pl. 11; [3], fig. 25a; [63], pl. 4a; [11], fig. 4). In some Philodromidae, the mesal AT may become a large and shallow mesal depression (MD) that covers most or all of the epigynal area and is better seen in posterior view, as in *Cleocnemis* and *Tibelloides* (Figs. 3d,e,g, 7d,e,g and 9d,e,g). This MD includes the whole MS length except by its posterior margin, which is elevated and sclerotized, in some cases forming a posterior rim (PR), and sometimes limited by small lateral plates (LP), CG and GP (Fig. 3d–g). In many groups, where a long MS divides most of the epigynal area in two lateral portions, the cavities at each side of MS are called bilateral atria (BA) following Schick [6] ([79], Fig. 4; [7], figs. 29, 31; [46], figs. 139, 210, 410). In some groups, the CO lies at the inner portion of a large and deep AT and it is not possible to recognize a CG or even the precise position of CO ([46], figs. 131, 152, 157, 176–179).

Instead of lateral guide pockets sensu Schick [6], we prefer to call simply guide pocket (GP) a pair of additional structures not directly related to the CO area and functionally and morphologically different from the CG. In this way, we avoid calling distinct structures by very similar names as in Schick [6]. GP are usually placed at the posterior lateral area of the epigynum ([46], fig. 97; [62], pl. 11; [3], fig. 25a), but they may also be found at the anterior area (Fig. 3d, e). Also, we do not apply the term atria to the cavities delimited by the GP keels, as they are not placed around CO, but may sometimes be merged with the CG and CA (as in *Thanatus* and *Tibellus*, ex. [62], pl. 11, *Paracleocnemis termalis* or “*Cleocnemis*” *lanceolata*). We avoid the use of epigynal grooves sensu Muster [3] as it is not necessary and may be misleading.

## Conclusions

All 14 species placed in *Cleocnemis* before this study were here scrutinized in order to solve the puzzle that this genus has become over the years. Firstly, by analyzing the original description of the type-species we concluded that *Cleocnemis* Simon, 1886 is a senior synonym of *Berlandiella* Mello-Leitão, 1929 and *Metacleocnemis* Mello-Leitão 1929. Eight species previously placed in the genus were included in our phylogenetic analyses based on molecular characters. It resulted in six distinct lineages for *Cleocnemis*, five within Thanatinae and one within Philodrominae.

However, combining these results with our morphological analysis, it indicated that 13 of the species previously included in *Cleocnemis* actually belong to five recognizable groups: *Cleocnemis *sensu stricto (Group I), including *C. heteropoda* and the species previously described in *Berlandiella*; *Tibelloides* **gen. rev.** (Group II); Group III, representing the genus *Fageia*; Group IV, including *“Cleocnemis” lanceolata* and *“Paracleocnemis” apostoli*; and Group V, including *“Cleocnemis” mutilata* and associated species. *Tibelloides* **gen. rev.** was the only taxon recovered as non-monophyletic, however, by the reasons exposed throughout discussion, it was treated as a recognizable and valid taxon. The only species previously placed in *Cleocnemis* which was not allocated in any taxonomic group, due to the lack of information, was *Cleocnemis nigra*, treated herein as *nomen dubium* and *incertae sedis*.

Our molecular phylogenies provide interesting implications on the systematics of the family and even on the composition of its subfamilies, Thanatinae and Philodrominae. Furthermore, this study helps to improve the understanding of Philodromidae systematics through the assessment of neglected Neotropical taxa, such as *Fageia* and *Cleocnemis *sensu lato, and also provides insights into the terminology of genital structures of the family.

## Supplementary Information

**Additional file 1. **List of additional material examined.

**Additional file 2. **Primers used for amplification and sequencing of molecular markers used in the phylogeny of Philodromidae. F – Forward, R – Reverse.

**Additional file 3. **Partition scheme and respective model suggested by BIC in ModelFinder for the concatenated molecular dataset of Philodromidae.

**Additional file 4. **Resulting phylogenetic trees of Philodromidae: (a) ML of concatenated matrix with clade supports of SH-aLRT/UFBoot, and BI analyses of (b) concatenated matrix, (c) COI, (d) H3, (e) 16S, and (f) 28S with posterior probabilities values.

## Data Availability

The collection data of the specimens used in this study are available within this article (and its additional files). The molecular datasets of cytochrome oxidase I (COI), histone H3, 16S rDNA and 28S rDNA genes generated to this study are available on GenBank® under the accession numbers OM773126-OM773137, OM936911-OM936924, OM913603-OM913615 and OM902669-OM902673 respectively.
