# Correction: Solving a running crab spider puzzle: delimiting *Cleocnemis* Simon, 1886 with implications on the phylogeny and terminology of genital structures of Philodromidae

**DOI:** 10.1186/s40850-022-00162-5

**Published:** 2023-01-18

**Authors:** André Wanderley do Prado, Renner Luiz Cerqueira Baptista, Hector Baruch Pereira Schinelli, Daniela Maeda Takiya

**Affiliations:** 1 Laboratório de Diversidade de Aracnídeos, Universidade Do Brasil/Universidade Federal Do Rio de Janeiro, Av. Carlos Chagas Filho 373, 21941-902, Ilha Do Fundão, Rio de Janeiro, RJ Brazil; 2 Programa de Pós-Graduação em Biodiversidade e Biologia Evolutiva, Universidade Federal Do Rio de Janeiro, Rio de Janeiro, RJ Brazil; 3 Laboratório de Entomologia, Departamento de Zoologia, Instituto de Biologia, Universidade Federal do Rio de Janeiro, Cidade Universitária, Rio de Janeiro, RJ 21941-902 Brazil

**Correction: BMC Zool 7, 51 (2022)**

**https://doi.org/10.1186/s40850-022-00136-7**

Following the publication of the original article, Prado et al. 2022 [1], authors reported that since the amendments to the International Code of Zoological Nomenclature (ICZN) [2] dealing with registration of nomenclatural acts in The Official Registry of Zoological Nomenclature (ZooBank) [3], in article 8 of the online electronic ICZN (https://www.iczn.org/the-code/the-code-online/), new requirements were made for availability of nomenclatural acts published in exclusively electronic journals. Those new demands led to an increasing need for errata and addenda works intended solely to meet these new ICZN´s requirements and avoid the unavailability of proposed nomenclatural acts on legitimate, original taxonomic works (ex. [4, 5]). Relevant questions have been raised about the easiness or the advisability of declaring unavailable or suppress nomenclatural acts of bona fide contemporary works on zoological taxonomy (ex. [6]), on the grounds that those procedures would hamper taxonomical progress and that they are “deeply intrusive into the intellectual freedom of scientific work”. Those questions would be better left to acceptance or not by the scientific community. Anyway, the amendments to article 8 of ICZN are mandatory and should currently be followed by all zoologists proposing nomenclatural changes.

Recently, we provide a proper ZooBank LSID publication code [urn:lsid:zoobank.org:pub:8881A48C-D0CC-4DE1-AB41-254F0F351561] for Prado et al. 2022 [1], that was not included in the original publication by lapsus. However, this procedure does not fulfill the requirements of ICZN article 8.5.3.3 in order to validate the nomenclatural proposals in our original publication. To avoid unavailability of our nomenclatural acts, this *erratum* was proposed as a separate publication, with its proper ZooBank LSID publication code [urn:lsid:zoobank.org:pub:EC98ACD1-C760-42C9-9345-F1B061B1606E]. All the nomenclatural acts proposed in Prado et al. (2022) [1] are made available in the present *erratum*, including new names, new combinations, new status, synonymies*, nomina dubia,* and genus transferences, all followed by its proper ZooBank LSID acts, when possible, and with cross-references to the rationale for each act in our original article. Unfortunately, the ZooBank is not yet fully implemented, dealing primarily with new scientific names or new combinations. However, other important and common taxonomical acts, such as establishing a new synonym or a new status do not yet receive proper ZooBank LSID nomenclatural acts codes at this stage.

Additionally, we also present a section for minor corrections, followed by a corrected “Additional material examined” section, published originally as Additional file 1 in Prado et al. (2022) [1]. Issues with the data of some lots are now corrected and presented herein in the new version of “Additional material examined”.

TAXONOMY

Order Araneae Linnaeus, 1758

Family Philodromidae Thorell, 1870

Subfamily Philodrominae Thorell, 1870 **status nov.**

Subfamily Thanatinae Schick, 1965 **status nov.**

As a result of our molecular phylogenetic results and our morphological discussion, we decided to elevate tribes Philodromini and Thanatini recognized by Schick (1965) [7] to the subfamily level (see [1], p. 6-7), thus establishing new status for those two taxa:

Philodrominae Thorell, 1870 **status nov.**

[urn:lsid:zoobank.org:act:735A2FAC-EF39-4A01-A0B6-DB113445085A]

Thanatinae Schick, 1965 **status nov.**

[urn:lsid:zoobank.org:act:88F3DCC2-9E23-44D0-AB5C-FA2FAC8FCD2D]

Genus *Cleocnemis* Simon, 1886

The type-species of the genus, *Cleocnemis heteropoda* Simon, 1886, is redescribed, with the proposition of a neotype (UFRJ 1562), and considered as the senior synonym of three other described species: *Berlandiella polyacantha* Mello-Leitão, 1929 **syn. nov.**, *Berlandiella meridionalis* Mello-Leitão, 1929 **syn. nov.** and *Metacleocnemis borgmeyeri* Mello-Leitão, 1929 **syn. nov.** ([1], p. 12). We also proposed *Cleocnemis* Simon, 1886 as senior synonym of *Metacleocnemis* Mello-Leitão, 1929 **syn. nov.** and *Berlandiella* Mello-Leitão, 1929 **syn. nov.** ([1], p. 12), taking in consideration that *M. borgmeyeri* is the only species of *Metacleocnemis*, and that *Cleocnemis heteropoda* shares all diagnostic characters with *Berlandiella*. Consequently, the following five new combinations for the other species of *Berlandiella* are herein proposed (see [1], p. 8):

*Cleocnemis insignis* (Mello-Leitão, 1929) **comb. nov.**

[urn:lsid:zoobank.org:act:7F8C3B20-6D56-4F61-B374-DCC2158251B3],

*Cleocnemis magna* (Mello-Leitão, 1929) **comb. nov.**

[urn:lsid:zoobank.org:act:8E9969A8-13E1-4A22-9980-8283EB1F1552],

*Cleocnemis querencia* (Lise & Silva, 2011) **comb. nov.**

[urn:lsid:zoobank.org:act:2A32CD66-72BE-485E-928F-D7579EA205D9]

*Cleocnemis robertae* (Lise & Silva, 2011) **comb. nov.**

[urn:lsid:zoobank.org:act:4ADB3474-8E8B-492A-A62F-001E897E024E]

*Cleocnemis zabele* (Pantoja, Drago-Bisneto & Saturnino, 2020) **comb. nov.**

[urn:lsid:zoobank.org:act:B850667E-9731-40DD-B3C1-D38BA89A1C47].

Another species clearly belonging to this genus is *Cleocnemis spinosa* Mello-Leitão, 1947, but it is considered a *nomen dubium* by now, as its holotype was not found at Museu Paranaense Capão de Imbuia and the original description and drawing do not allow a sure identification (see [1], p. 8).

Genus *Tibelloides* Mello-Leitão, 1939 **gen. rev.**

We also revalidated *Tibelloides* Mello-Leitão, 1939 **gen. rev.**, removing it from the synonymy of *Tibellus* Simon, 1875 (see [1], p. 8), and transferred four species previously placed in *Cleocnemis* to it, resulting in three new combinations and one *nomen novum* (see [1], p. 8, 21):

*Tibelloides bryantae* (Gertsch, 1933) **comb. nov.**

[urn:lsid:zoobank.org:act:F94DB3CC-65E6-4D36-B7A8-4BDF808E1DF1]

*Tibelloides punctulatus* (Taczanowski, 1872) **comb. nov.**

[urn:lsid:zoobank.org:act:46A45248-1FAA-4644-8620-DE498377BDD3]

*Tibelloides reimoseri* **nom. nov.** (new name for *Apollophanes paraguensis* Gertsch, 1933) [urn:lsid:zoobank.org:act:79A6A69A-D8E6-4B17-86BF-54714087E929]

*Tibelloides taquarae* (Keyserling, 1891). **comb. nov.**

[urn:lsid:zoobank.org:act:83E8083C-068A-4187-825B-B6CF29442756].

*Tibelloides punctulatus* (Taczanowski, 1872) **comb. nov.** is considered senior synonym of *Tibelloides spatuliferus* Mello-Leitão, 1939 **syn. nov.** and *Tibellus paraguensis* Simon, 1897 **syn. nov.** (see [1], p. 21). *Tibelloides bryantae* Gertsch, 1933) **comb. nov.** is considered senior synonym of *Cleocnemis rudolphi* Mello-Leitão, 1943 **syn. nov**. (see [1], p. 21). *Tibelloides reimoseri* is proposed as *nomen novum* for *Apollophanes paraguensis* Gertsch, 1933 (see [1], p. 22-23) to avoid secondary homonym with *Tibellus paraguensis* Simon, 1897, which is treated as a junior synonym of *Tibelloides punctulatus* (Taczanowski, 1872) **comb. nov.**

Genus *Fageia* Mello-Leitão, 1929

Two species placed in *Cleocnemis* previous to our original work were transferred to *Fageia* Mello-Leitão, 1929, resulting in the following new combinations (see [1], p. 28):

*Fageia moschata* (Mello-Leitão, 1943) **comb. nov.**

[urn:lsid:zoobank.org:act:15A46C76-62D4-4AEC-B629-C78C4F1EBEDD]

*Fageia rosea* (Mello-Leitão, 1944) **comb. nov.**

[urn:lsid:zoobank.org:act:FF65F06E-2FC9-470E-8E28-B5BBDCCE02AA]**.**

Species *incertae sedis*, *nomina dubia* and other nomenclatural acts

Finally, we summarize below the *nomina dubia*, *incertae sedis* and remaining nomenclatural acts, proposed in Prado et al. (2022) [1]:

*“Cleocnemis” mutilata* Mello-Leitão, 1917 is deemed as senior synonym of *Gephyrina imbecilla* Mello-Leitão, 1917 **syn. nov.**, but also *incertae sedis*, as it does not belong to *Cleocnemis* and should be included in a new genus we are describing (see [1], p. 8, 28).

*“Cleocnemis” xenotypa* Mello-Leitão, 1929 and *“Cleocnemis” serrana* Mello-Leitão, 1929 are both also *incertae sedis*, related to *“Cleocnemis” mutilata*. Furthermore, they can not be recognized by now and are considered also as *nomina dubia* (see [1], p. 8)*.*

*“Paracleocnemis” apostoli* Mello-Leitão, 1945 is removed from the now monotypic genus *Paracleocnemis* Schiapelli & Gerschman, 1942, and treated as *incertae sedis* (see [1], p. 28), as it should be included in a new genus we are describing.

*“Cleocnemis” nigra* Mello-Leitão, 1943 is considered a *nomen dubium*, as its succinct description without illustration does not allow recognition and its holotype was lost (MNRJ). Besides its scarcity of details, the original description clearly allows its removal from *Cleocnemis*, but it is not enough to include the species in another Philodromidae genus. Thus, *“Cleocnemis” nigra* is considered *incertae sedis* (see [1], p. 8)*.*

The monotypic genus *Procleocnemis* Mello-Leitão, 1929, alongside its type-species *Procleocnemis concolor* Mello-Leitão, 1929, is transferred to Thomisidae and considered *incertae sedis*, but is clearly a Misumeninae, probably near *Tmarus* Simon, 1875, *Titidius* Simon, 1895 or related genera (see [1], p. 29)*.*

MINOR CORRECTIONS ON THE MAIN TEXT

The distribution section of *Tibelloides* in the original text starts with “The five described species of *Tibelloides* are found…” ([1], p. 20), however there are only four valid described species for the genus as stated in the genus composition just some lines before in the original publication.

A corrected distribution section of *Tibelloides bryantae* is provided below:

*Tibelloides bryantae* is found in Paraguay and Brazil, with records from Northeastern and Southeastern Brazilian regions.

CORRECTED ADDITIONAL MATERIAL EXAMINED

*Cleocnemis heteropoda*

Additional material examined. **BRAZIL: Minas Gerais:** Inconfidentes, Bairro Monjolinho, Fazenda Santa Luzia, 22.3336°S, 46.2974°W, 1♀, i-ii.2015, Souza, M. M. (UFMG 19728); **Paraná:** Ponta Grossa, Fazenda Paiquerê, 1♀, ii.2002, Nascimento, E. (IBSP 040980), São José dos Pinhais, 25.605°S, 49.1936°W, 18♂, 6♀, 02.i.2014-01.vii.2015, Domahovski, A.C. (MCTP 39023), 2♂, 9.xii.1986, Eq. Profaupar (MCTP 19445), 2♂, 10.xi.1986, Eq. Profaupar (MCTP 19443), 1♂, 3.xi.1986, Eq. Profaupar (MCTP 19447), 3♂, 5♀, 09.xii.2015, Domahovski, A.C. (MCTP 39087), 2♂, 24.xi.1986, Eq. Profaupar (MCTP 19446), Tijucas do Sul, Morro do Cabral, Lagoa,1♂, x.2000, Ricetti, J. (IBSP 039237), 1♀ x.2000, Ricetti, J. (IBSP 039153), 1♀ x.2000, Ricetti, J. (IBSP 039231); **Rio de Janeiro:** Angra dos Reis, Ilha Grande, 1♂, xi.2017, Queiroz, R., Silveira, L., Campello, L. (UFRJ 1641); Angra dos Reis, Ilha Grande, Pico do Papagaio, 2♂, i.2018, Campello, L., Queiroz, R., Silveira, R. (UFRJ 1645), 1♂ i.2018, Campello, L., Queiroz, R., Silveira, R. (UFRJ 1637), 1♂, xii.2017, Queiroz, R., Silveira, L., Campello, L. (UFRJ 1648); Rio de Janeiro, PN da Tijuca, 1♂, 04.x.2016, Eq. Lab. Entomologia UFRJ (UFRJ 1562), 1♂, 04.x.2016, Eq. Lab. Entomologia UFRJ (UFRJ 2000), Rio de Janeiro, PN da Tijuca, Archer, 2♀, 19.i.2005, Moreira, T. S. (MNRJ 06527), Rio de Janeiro, PN da Tijuca, Gávea, 1♀, 18.i.2005, Villareal M. O. (MNRJ 06526), 1♀, 18.i.2005, Villareal M. O. (MNRJ 06525), Teresópolis, PN da Serra dos Órgãos, P2, 1♂, 1♀, 29.xi.2013, Prado, A.W. (UFRJ 1630), 2♂, 04.i.2014, Equipe LEI (Lab. Ecol. Insetos) (UFRJ 1632), Teresópolis, PN da Serra dos Órgãos, P3, 3♂, 21.xi.2013, Equipe LEI (Lab. Ecol. Insetos) (UFRJ 1631), Teresópolis, PN da Serra dos Órgãos, Trilha Suspensa, 5♂, 6♀, 16.xii.2019, Oliveira, G.A. & Schinelli, H. B. (UFRJ 1650), 3♂, 1♀, 16.xii.2019, Baptista, R. L. C. (UFRJ 1644), 2♂, 3♀, 16.xii.2019, Prado, A. W. (UFRJ 1647), 1♀, 16.xii.2019, Oliveira, G.A. & Schinelli, H. B. (UFRJ 1638), 1♀, 18.xii.2019, Schinelli, H. (UFRJ 1640), Teresópolis, PN Serra dos Órgãos, 2♂, 4.i.2014, Lab. Ecol. Insetos, UFRJ (UFRJ 1969), 1♂, 1♀, 29.xi.2013, Lab. Ecol. Insetos, UFRJ (UFRJ 1970); **Rio Grande do Sul:** Cambará do Sul, Itaimbezinho, 29.0478°S, 50.1447°W, 1♀, 1J, 6.i.1985, Lise, A. (MCTP 21419), São Francisco de Paula, 29.4481°S, 50.5836°W, 1♂, 4♀, 09-12.i.1997, Lise, A. (MCTP 10743), São Francisco de Paula, Potreiro Velho, 29.4806°S, 50.175°W, 5♀, 9.x.1994, Braul, A. & Ott, R. (MCTP 05483), 3♀, 21-24.iii.1995, Lise, A. (MCTP 11990), 1♂, 3♀, 5-9.xii.1997, Lise, A. (MCTP 15972), 3♂, 13♀, 14-17.xii.1996, Lise, A. et al. (MCTP 10959), 2♂, 2♀, 12-15.xi.1998, Lise, A. et al. (MCTP 14386), 1♂, 1♀, 24.x.1996, Ott, R. (MCTP 10664), 3♂, 7♀, 5-8.xii.1996, Lise, A. et al. (MCTP 13898), 7♂, 2♀, xii.2001, Bertoncello, L. A. & Lise, A. (MCTP 19476), 1♂, 1♀, xii.2001, Bertoncello, L. A. & Lise, A. (MCTP 19477), São Francisco de Paula, Potreiro Velho, Pró-Mata, 29.4481°S, 50.5836°W, 1♂, 1♀, 24.x.1996, Ott, R. (MCTP 10665), 2♂, x.2001, Bertoncello, L. A. (MCTP 19463), **Santa Catarina:** Blumenau, Parque Natural Municipal Nascentes do Garcia, 27 01 S, 49 09 W, 27.0167°S, 49.15°W, 1♀, 21-28.i.2003, Eq. Biota Fapesp (IBSP 138316), 1♀, 21-28.i.2003, Eq. Biota Fapesp (IBSP 138309), 1♀, 21-28.i.2003, Eq. Biota Fapesp (IBSP 138314), Florianópolis, Costão do Santinho, Morro das Aranhas, 1♀, 2007, F. Albertoni (IBSP 144188); **São Paulo:** Mogi das Cruzes, PNM Serra do Itapety, 23 29 S, 46 12 W, 23.4833°S, 46.2°W, 1♂, 13-19.x.2003, Eq. Biota Fapesp (IBSP 138233), Peruíbe, EE Juréia-Itatins, Despraiado, 1♂, 30.ix.1997, Bertani, R. (IBSP 013540), Salesópolis, EE Boracéia, 23.5333°S, 45.85°W, 1♀, 18-24.v.2001, Eq. Biota Fapesp (IBSP 138241), 2♀, 26-28.i.1999, Pinto-da-Rocha, R., Casari, Ram (MZSP 17525), 1♀, 26-28.i.1999, Pinto-da-Rocha, R., Casari, Ram (MZSP 17490), Santo André, Alto da Serra do Paranapiacaba, 2♂, 3♀, 14-16.xii.2003, Rheims, CA & Indicatti, R (IBSP 052082), São Luiz do Paraitinga, PE da Serra do Mar, Núcleo Santa Virgínia, 1♀, 21.i.2008, Maciel-Silva, A. S. (UFMG 06231).

*“Cleocnemis” lanceolata*

Additional material examined. **BRAZIL**: **Bahia**: Condeúba [ex Santo Antônio da Barra], 2♂, 1♀, [no date] (*Cleocnemis heteropoda*: Mello-Leitão det., MNHN 11501); **Mato Grosso**: Pontes e Lacerda, Usina Hidroelétrica de Guaporé, Vale São Domingos, 1♀, x.1999, Equipe Resgate (IBSP 041488B); **Mato Grosso do Sul**: Bonito, Anhumas , 21.1667°S, 56.5833°W, 1♀, 14-23.x.2002, Eq. BIOTA (IBSP 138362), Bonito, Baía Bonita, 21.15°S, 56.4333°W, 1♀, 14-23.x.2002, Eq. BIOTA (IBSP 138374), 1♀, 14-23.x.2002, Eq. BIOTA (IBSP 138365), 1♀, 14-23.x.2002, Eq. BIOTA (IBSP 13870); **Paraná**: Boa Vista da Aparecida, Flor da Serra, 1♂, 7-14.x.1996, Eq. IBSP (IBSP 21338A), Cascavel, Lago Municipal, 1♀, 13.i.2007, Silva, A. P. G. (IBSP 227801), Foz do Iguaçu, 25.5478°S, 54.5881°W, 1♂, 1♀, 16.xi.1991, Bonaldo, A.B. & Rodrigues, B.V.B (MCTP 01652); **Rio Grande do Sul**: Agudo, 1♂, 02.xi.2002, Morais, A.B.B. (MCTP 40437), Augusto Pestana, 1♀, 27.xii.2008, Silva, L. V. *et al.* (MCTP 26935), 1♀,Silva, L.V., Medeiros, L.B. (MCTP 30448), Cachoeira do Sul, Alto dos Casemiros, 2♀, 03.i.1994, Buss, R. G. (MCTP 04419), Cachoeira do Sul, Capanezinho, 30.0392°S, 52.8939°W, 2♀, 02.xi.1992, Buss, R. G. (MCTP 03324), Erval Grande, 1♀, i.1994, Braul, A. (MCTP 04490), Pântano Grande, 1♀, 05.iv.2008, Depra, G. (MCTP 40429), 2♀, 08.xii.2007, Depra, G. (MCTP 40430), Santa Cruz, 1♂, 1J, 06.iii.1994, Ott, R. leg. (MCTP 06571), Santa Maria, 2♂, 1♀, 2J, 16.xi.1998, Kotzian, C. B.; Indrusiak, L. (MCTP 39762), 3♀, 19.xi.1998, Kotzian, C.B., Indrusiak, L. (MCTP 40444), 1♂, 2♀, 16.xii.1997, Indrusiak, L., Monteiro, M. (MCTP 40434A), 1♀, 29.i.1998, Indrusiak, L., Monteiro, M. (MCTP 40432), 3♀, [no date], Kotzian, C. B.; Indrusiak, L. (MCTP 40431), Santa Maria, Cidade dos Meninos, 2♀, 22.i.1996, Ketzian, C.B., Indrusiak, L. (MCTP 40439), Santa Maria, Lar Metodista, 29.6842°S, 53.8069°W, 1♀, 15.xii.2004, Indrusiak, L. (MCTP 41414), Santa Maria, Perau Velho, 1♀, 19.01.1995, Ketzian, C.B., Indrusiak, L. (MCTP 40440), Santa Maria, São Marcos, 2♀, 10.i.1996, Ketzian, C.B., Indrusiak, L. (MCTP 40438), São Borja, Reserva Biológica São Donato, 28.6606°S, 56.0044°W, 1♀, 28.i.2012, Machado, M. (MCTP 34711), 1♀, 05.iii.2013, Machado, M. (MCTP 43389), 2♀, 05.iii.2013, Machado, M. (MCTP 43390), São Borja, Reserva São Donato, 28.6606°S, 56.0044°W, 1♀, 11.x.2012, Machado, M. (MCTP 36951), Vicente Dutra, 1♀, 07.ii.2006, Trescher, T.F. (MCTP 22528); **Santa Catarina**: Chapecó, Quebra-Queixo, 1♀, 26-27.ii.2002 (MCTP 12892), São Cristóvão do Sul, Monte Alegre, 1♀, 2001-2002, Moreira, J. M. (IBSP 141470); **São Paulo**: Barueri, 1♀, 14.xii.1965, Linka, K. (MZUSP 13299), Itapetininga, 1♀, 11-16.xi.2002, Equipe BIOTA (IBSP 138275). **PARAGUAY**: **Alto Paraguay [ex Chaco]**: PN Defensores del Chaco, Cerro León: Sítio #24: Vale Pupukú, 1♀, 25.xi.1984, Kochalka, J.A. (IBNP 2580B); **Amambay**: Chacurrú, 1♀, 9-11.xi.2018, Piñanez, Y. (IBNP 50025); **Canindeyú**: Mbaracayú, 1♀, 6. xi.2018, Piñanez, Y. (IBNP 2960); **Central**: Asunción, Jardín Botánico, 1♂, 14.i.1994, Kochalka, J.A. (IBNP 2584); **Itapúa**: Capitán Miranda, Hotel El Tirol, 27.1833°S, 55.7778°W, 1♂, 1♀, 16-19.xi.2018, Piñanez, Y. (IBNP 50010); **Paraguari**: Paraguari, Cerro Hú, 1♀, 2.xi.2018, Piñanez, Y. (IBNP 2994), PN Ybycui, 1♀, 31.x-7.xi.1990, Herranz, J. (IBNP 2905); **San Pedro**: Santa Barbara, RN Laguna Blanca, 1♂, 1♀, 21.ix.2011, Recalde, F. (IBNP 2646).

*“Cleocnemis” mutilata*

Additional material examined. **BRAZIL**: **Espírito Santo**: Apiacá, Fazenda Santa Maria, 21.1538°S, 41.5664°W, 1♀, 23.xi.1996, Baptista, R. L. C. (MNRJ 06517); **Minas Gerais**: Belo Horizonte, 19.9167°S, 43.9345°W, 1♂, 1J, 01.vi.1993, Dutra, G. F. (IBSP 026815); 2♀, 13.vii.1993, Ferreira, A.H.P. & Santos, A.J. (IBSP 026816); **Rio de Janeiro**: Bom Jesus do Itabapoana, 21.1453°S, 41.6826°W, 1♀, 16.xi.1986, Baptista, R. L. C. (MNRJ 06493); 1♂, 29.viii.1987, Baptista, R. L. C. (MNRJ 06494); Casimiro de Abreu, Fazenda, 22.4798°S, 42.2029°W, 1♀, 04-13.viii.2010, Tinoco, D.C. (UFRJ 2014); Macaé, Colégio Matias Neto, 22.3731°S, 41.7813°W, 1♀, 30.xi.2010, Gabriela (UFRJ 0561); Macaé, Escola J. Calil Filho, 22.3288°S, 41.7396°W, 1♂, 7.vii.2010, Gabriela (UFRJ 0562), Rio de Janeiro, Ilha do Fundão, Catalão, 22.8445°S, 43.2213°W, 1♂, 12J, 5.x.2018, Prado, A. W. & Baptista R. L. C (UFRJ 1535); 3♂, 2♀, 13J, 11.vii.2019, Prado, A. W. (UFRJ 1643), Rio de Janeiro, Ilha do Fundão, Catalão, Forte, 22.8445°S, 43.2213°W, 3♂, 1♀, 1J, 31.x.2018, Prado, A. W. & Baptista R. L. C (UFRJ 1536), 1♂, 1♀, 5J, 10.xii.2018, Prado, A.W. & Schinelli, H.B. (UFRJ 1537), Rio de Janeiro, Ilha do Fundão, Encruzilhada Vila Residencial, 22.8646°S, 43.2196°W, 2♂, 1J, 20.ix.2019, Guimarães, C.A; Oliveira, F.S.M. (UFRJ 1642), Rio de Janeiro, Museu da República, 22.9259°S, 43.1762°W, 1♂, 15.ii.1996, Baptista, R. L. C. (MNRJ 06518); **São Paulo**: Mogi das Cruzes, São João, 23.5262°S, 46.1885°W, 1♀, 28.ix.2003, Lemos, R. Y. (IBSP 041835); Santos, 23.9475°S, 46.3367°W, 1♀, 16.ii.2005, Prefeitura Municipal de Santos (IBSP 051230); São Paulo, 23.5508°S, 46.6356°W, 1♀, 28.iii.1996, Ribeiro, M. H. (IBSP 014037), 1♂, 04.ix.2005, Machado, E. C. (IBSP 056258), 1♂, 06.iv.2005, Vieira, A. (IBSP 051337), 1♂, 4♀, 11J, [no date], Cunha, F. S. (IBSP 035768), 4♂, 5♀, 15J, [no date], Candiani, D. F. (IBSP 035771), 1♀, 2J, [no date], Candiani, D. F. (IBSP 035782); São Paulo, Instituto Butantan, campus, 23.5678°S, 46.7188°W, 1♂, ix.2007, Borreli, C. (IBSP 123975); São Paulo, Jardim Rizzo, URB, USP, 23.5614°S, 46.7308°W, 1♂, 1♀, 04.ii.1999, Candiani, D. F. (IBSP 028799); 1♀, 09.iii.1999, Castro, M. P. (IBSP 028873), 1♂, 24.ii.1999, Cunha, F. S. (IBSP 028818), 2♀, 24.ii.1999, Cunha, F. S. (IBSP 028836); 1♀, 09.xi.1998, Brescovit, A.D. (IBSP 020677); 1♀, 11.iii.1999, Candiani, D. F. (IBSP 028966); 1♀, 01.iii.1999, Cunha, F. S. (IBSP 028889); São Paulo, Pinheiros, 23.5636°S, 46.6916°W, 1♀, 10.iii.2002, Cunha, F. S. (IBSP 033234); São Paulo, USP, Cidade Universitária, 23.5614°S, 46.7308°W, 1♂, 18.i.2000, Candiani, D. F. (IBSP 033351); 1♀, 12.v.2003, Cizauskas, i. (IBSP 131624); São Paulo, Vila Butantan, URB, USP, 23.5614°S, 46.7308°W, 1♀, 08.xii.200, Cunha, F. S. (IBSP 032968).

*Tibelloides bryantae*

Additional material examined. **BRAZIL**: **Bahia**: Iraquara, Pratinha, 12.3525°S, 41.5417°W, 1♂, 20.xii.1998, Rocha, L. S. (IBSP 020765), Senhor do Bonfim, UNEB, Campus VII, 1♀, iii-vii.2008, Costa, J. S. (IBSP 133758), 1♂, iii-vii.2008, Costa, J. S. (IBSP 133759), 1♂, iii-vii.2008, Costa, J. S. (IBSP 133760), 1♂, iii-vii.2008, Costa, J. S. (IBSP 133761), 1♀, iii-vii.2008, Costa, J. S. (IBSP 133762); **Minas Gerais:** Caeté, Gandarela, campos rupestres, 1♀, 5J, 22.i.2019, Prado, A. W. do (UFRJ 1542), Serra do Cipó, PN da Serra do Cipó, Cânion, 1♀, 04.xi.2021, Prado, A. W. do, Schinelli, H. S., Baptista, R. L. C. (UFRJ 2001); **Pernambuco**: Buíque, PN do Catimbau, Terra 01, 1♂, 14.iii.2019, Takiya, D.M. (UFRJ 1561), Buíque, PN do Catimbau, terra 02, 1♀, 14.iii.2019, Takiya, D.M. (UFRJ 1560); **Piauí:** PN da Serra das Confusões, Terra 1, 1♂, 2J, 10.xi.2018, Prando, J.S. (UFRJ 2002); **Rio Grande do Norte**: Parnamirim, Mata do Jiqui, EMP ARN - Empresa de Pesquisa Agropecuária do RN, 1♂, 24.ix.2008, Guedes, T. B. (IBSP 216559), 1♂, 24.ix.2008, Guedes, T. B. (IBSP 216183), 1♂, 24.ix.2008, Guedes, T. B. (IBSP 216253), 1♀, 24.ix.2008, Guedes, T. B. (IBSP 216266), 1♂, 24.ix.2008, Guedes, T. B. (IBSP 216293), 1♂, 24.ix.2008, Guedes, T. B. (IBSP 216304), 1♂, 24.ix.2008, Guedes, T. B. (IBSP 216339), 1♂, 17.ix.2008, Guedes, T. B. (IBSP 216909), 1♂, 17.ix.2008, Guedes, T. B. (IBSP 216624), 2♂, 17.ix.2008, Guedes, T. B. (IBSP 216714), 1♂, 17.ix.2008, Guedes, T. B. (IBSP 216761), 1♂, 17.ix.2008, Guedes, T. B. (IBSP 216825), 1♂, 17.ix.2008, Guedes, T.B. (IBSP 216697). **PARAGUAY**: **Amambay**: Chacurrú, 1♂, 2♀, 9.xi.2018, Piñanez, Y. (IBNP 50028); **Central**: Villeta, 2♀, 25.xi.2018, Piñanez, Y. (IBNP 2978); **Itapúa**: Alto Vera, Estación Biológica Kanguery: Campo natural, Estación n, 26.5167°S, 55.7833°W, 1♀, 12-16.i.2017, Piñanez, Y. (IBNP 2651), Nueva Arbolada, Museo del Árbol, 1♀, 19.xi.2018, Piñanez, Y. (IBNP 50070), San Miguel Potrero, entre Coronel Bogado y General Artigas, cauce del Arroyo Kambay, 1♀, 17.i.1996, Kochalka, J.A. (IBNP 2589); **Paraguari**: Paraguari, Hotel Gabriela, 1♂, 4.xi.2018, Piñanez, Y. (IBNP 2990).

*Tibelloides punctulatus*

Additional material examined. ARGENTINA: **Misiones**: San Javier, 1♀, 11-21.iv.1989, Proj. Garabi (MCTP 00562). BRAZIL: **Acre**: Rio Branco, Embrapa, Campus, 10.0252°S, 67.6933°W, 1♂, xi.2012, Costa, L. M. S. (UFMG 12433), 3♂, 1J, xii.2012, Costa, L. M. S. (UFMG 14152); **Mato Grosso**: Canarana, Beira do Rio Kuluene, 1♀, [no date], Falatti, C.Q. & Ribeiro, A.K. (IBSP 027062), Vale de São Domingos/Pontes e Lacerda, UHE de Guaporé, 1♀, x.1999, Equipe Resgate (IBSP 041487B); **Mato Grosso do Sul**: Anaurilândia, 2♂, 2♀, 12-19.iii.2001 , Cunha, F.S. & Souza, C.A.R. (IBSP 039294), Anaurilândia, UHE Engenheiro Sérgio Motta, 22.3831°S, 52.8118°W, 1♂, 7♀, 2J, 15.xi-23.xii.1998, Equipe IBSP (IBSP 023424), Brasilândia, UHE Sérgio Motta, 1♀, 2000, Equipe IBSP (IBSP 035515), Corumbá, , 1♀, 1994, Raizer, J. (IBSP 006474), Corumbá, Passo do Lontra, abobral, 1♀, 1997, Raizer, J. (IBSP 011442), 1♂, 1997, Raizer, J. (IBSP 011445), Dois Irmãos do Buriti, APP Rio Cachoeirão, Fazenda Taruana, 1♂, 1♀, 16-26.ii.2008, Bessi, R. & Bervian, C. (IBSP 167198), 1♀, 16-26.ii.2008, Bessi, R. & Bervian, C. (IBSP 215238), Dois Irmãos do Buriti, Fazenda Taruana, 2♂, 3♀, 16-26.ii.2008, Bessi, R. & Bervian, C. (IBSP 221484), Dourados, 1♂, 1♀, 23.ii.1982 (MCTP 18950), 1♀, 23.iii.1982 (MCTP 18954), 1♂, 01.iii.1982 (MCTP 18953), 1♂, 23.ii.1983 (MCTP 18952), Nhecolândia, Fazenda do Leque, EMBRAPA, 3♀, 11.xi.1987, Harris, S. (MNRJ 02631), Passo do Lontra, Base de Estudos do Pantanal - UFMS, 1♀, 22.v.1993, Raizer, J. (IBSP 020510), 1♀, 22.v.1993, Raizer, J. (IBSP 020513), Santa Rita do Rio Pardo, 1♀, 24.iv.2001, Bertani, R. & Kashimata, E.K. (IBSP 039528A); **Minas Gerais**: Jaboticatubas, PN da Serra do Cipó, 1♀, 28.ii-8.iii.2002, Álvares, E. S. S. (IBSP 138345), Montes Claros, UFMG, 1♀, 2001, Leite, G.L.D. (IBSP 036922), Prudente de Morais, 1♂, viii.2005, Morais, P. (IBSP 099084), Prudente de Morais, Fazenda Sapé, 19.5°S, 44.1167°W, 1♀, 02.ix.2000, Álvares, E. S. S. (UFMG 00618), Unaí, UHE Queimado, 1♀, viii.2003, Machado, E. O. (IBSP 072578); **Pará**: Altamira, Novo Progresso, 7.15194°S, 55.3056°W, 1♀, 20.xi.2005, Santos-Souza, D. R. (MPEG 002720), Marabá, Mina do Sossego, Serra Norte, Carajás, 6.60278°S, 50.3286°W, 1♀, 05-06.iii.2004, Marreco-Pedroso, A. (MPEG 003971), 2♂, 2♀, 05-06.iii.2004, Marreco-Pedroso, A. (MPEG 003974), 1♂, [no date], Marreco-Pedroso, A. (MPEG 003977), 1♀, [no date], Wanzeler, E. (MPEG 003978), 1♂, 2♀, 03.iii.2004, Marreco-Pedroso, A. (MPEG 003979), 1♂, 2♀, 06.iii.2004, Marreco-Pedroso, A. (MPEG 003981), 1♀, [no date] (MPEG 003983), 1♂, 06.iii.2004, Lobo, D. S. (MPEG 003984), 1♂, 05.iii.2004, Wanzeler, E. (MPEG 003986), 1♀, 06.iii.2004, Lobo, D. S. (MPEG 003987), 3♂, 6♀, 06.iii.2004, Wanzeler, E. (MPEG 003988), 1♂, 1♀, 2J, 06.iii.2004, Marreco-Pedroso, A. (MPEG 004103), 1♀, 06.iii.2004, Marreco-Pedroso, A. (MPEG 004104), 1♂, 06.iii.2004, Marreco-Pedroso, A. (MPEG 004105), 1♂, 1J, 06.iii.2004, Santos-Souza, D. R. (MPEG 004106), 4♂, 2♀, 2J, 03.iii.2004, Wanzeler, E. (MPEG 004107), 2♂, 23.ii-06.iii.2004, Fernandes, J. A. M. (MPEG 004108), 1♀, 03.iii.2004, Lobo & Wanzeler (MPEG 004099), **Paraná**: Boa Vista da Aparecida, Flor da Serra, 1♂, 1♀, 7-14.x.1998, Equipe IBSP (IBSP 021107), Capitão Leônidas Marques, Salto Caxias, Rio Iguaçu, 1♂, 5♀, 28.ii.1993, Bonaldo, A. (MCTP 04324), Céu Azul, 6♂, 4♀, 10J, 10.ii.2020, Prado, A.W. do (UFRJ 1971), Cruzeiro do Iguaçu, 1♀, 8-15.x.1998, Eq. IBSP (IBSP 021385), 1♂, 8-15.x.1998, Eq. IBSP (IBSP 021388), Dois Vizinhos, UHE de Salto Caxias, Foz do Chopim, Cruzeiro do Iguaçu, 1♀, 8-15.x.1998, Equipe IBSP (IBSP 021047), Francisco Beltrão, 1♂, 1♀, 4J, 09.ii.2020, Prado, A.W. do & Baptista R.L.C. (UFRJ 1973), Pinhão/Candói, Usina Hidrelétrica Santa Clara, Rio Jordão, 25.6478°S, 51.9536°W, 1♀, 2005, Ricetti, J. et. al. (IBSP 143576); **Rio de Janeiro**: Rio de Janeiro, Campo dos Afonsos, 1♂, 13.xi.2008, Eq. Lab. Entomologia Forense UCB (UFRJ 0481), 1♀, 29.x.2008, Eq. Lab. Entomologia Forense UCB (UFRJ 0484), 1♂, 23.xi.2008, Eq. Lab. Entomologia Forense UCB (UFRJ 0483), 1♀, 30.iv.2009, Eq. Lab. Entomologia Forense UCB (UFRJ 0486), 1♀, 22.x.2008, Eq. Lab. Entomologia Forense UCB (UFRJ 0482), 1♂, 21.iv.2009, Eq. Lab. Entomologia Forense UCB (UFRJ 0485), Rio de Janeiro, Ilha do Fundão, Capim Limão, 1♀, 7.viii.2012, Luisa (UFRJ 0611), Rio de Janeiro, Pedra de Guaratiba, APA das Brisas, 1♂, 2♀, 1J, 13.xi.2018, Prado, A.W. do & Baptista R.L.C. (UFRJ 1539); **Rio Grande do Sul**: Vicente Dutra, 1♂, 2J, 07.ii.2020, Prado, A.W. do & Baptista R.L.C. (UFRJ 1974); **São Paulo**: Avanhandava, 2♀, x.1982, Vizotto et al. (IBSP 004182), 1♂, 2♀, x.1982, Vizotto, Dino, L. (IBSP 042639), Onda Verde, Fazenda São João, 4♂, 1♀, 6J, i.1946, Lane, F. (MZSP 13297), Presidente Epitácio, UHE Sérgio Motta, 1♀, 16.i - 13.ii.1999, Equipe IBSP (IBSP 023118), Presidente Epitácio, UHE Sérgio Motta, Ilha da Lagoa do Machado, 1♀, 30.iii.2001, Indicatti, R.P. & Souza, C.A.R. (IBSP 038507), Primavera, Porto Primavera, 1♀, 23.ii.2001, Candiani, D. F. & Indicatti, R. P. (IBSP 039339B), Primavera, UHE Sérgio Motta, 1♀, i-ii.2000, Equipe BIOTA (IBSP 029650), 5♂, 15♀, 1J, i-ii.2000, Equipe BIOTA (IBSP 029738A), 6♂, 17♀, i-ii.2000, Equipe BIOTA (IBSP 029860A), 6♂, 13♀, i-ii.2000, Equipe BIOTA (IBSP 029873A), 1♀, i-ii.2000, Equipe BIOTA (IBSP 029881A), 4♂, 4♀, i-ii/2000, Equipe IBSP (IBSP 029897), Rosana, UHE Rosana, 2♂, 2♀, 3J, xii.1986, Equipe IBSP (IBSP 004463), São Paulo, 1♂, 1♀, [no date], Cunha, F.S. (IBSP 035777B), São Paulo, Jardim Rizzo URBUSP, 1♀, 24.ii.1999, Cunha, F.S. (IBSP 028834), Três Irmãos, Três Irmãos, 1♂, x.1990, Costa & Bertim (IBSP 004878); **Tocantins**: Gurupi, 11.7458°S, 49.0525°W, 2♂, 29.vi.2015, Tschoeke, O.H. (UFMG 19056), Miracema, UHE Luis Eduardo Magalhães, 1♂, 11-21.x.2001, Bertani, R. & Toledo, W.I. (IBSP 031499), Palmas, Jardim Taquari, Taquaralto, 1♀, 4-10.xi.2002, Knysak, I. & Martins, R. (IBSP 219564), Palmas, Rio Tocantins, margem direita, 1♀, i.2002, Candido, D.M. & Costa, M. (IBSP 040431). PARAGUAY: **Alto Paraguay**: PN Defensores del Chaco, Madrejón, 1♀, 8.xii.1981, Kochalka, J.A. (IBNP 2578), 1♀, 16.xii.1981, Kochalka, J.A. (IBNP 2579); **Amambay**: Chacurrú, , 1♂, 1♀, 9-12.xi.2018, Piñanez, Y. (IBNP 50024); **Central**: San Lorenzo, Infona, 1♂, 1J, 17.iii.2016, Piñanez, Y. (IBNP 2595), Villeta, 1♂, 4♀, 5J, 02.ii.2020, Prado, A.W. & Baptista, R.L.C. (UFRJ 1976); **Cordillera**: San Bernardino, 1♂, 7-8.xi.1987, Kochalka, J.A. (IBNP 2587), 9♂, 9♀, 15J, 31.i.2020, Prado, A.W. do & Baptista, R.L.C. (UFRJ 1972); **Guairá**: Colonia Independencia, Hotel Tilinski, 2♂, 2♀, 1J, 14.xi.2018, Piñanez, Yolanda (IBNP 2987); **Itapúa**: Carmen del Paraná, 1♀, 17.i.1996, Kochalka, J.A. (IBNP 2588), Nueva Arbolada, Museo del Árbol, 1♀, 19.xi.2018, Piñanez, Y. (IBNP 2969); **Paraguari**: Cerro Hú, Hotel Gabriela, 1♂, 1♀, 4J, 01.ii.2020, Prado, A.W. do & Baptista R.L.C. (UFRJ 1975).
