# Supplementary material for: Solving a running crab spider puzzle: delimiting Cleocnemis Simon, 1886 with implications on the phylogeny and terminology of genital structures of Philodromidae
Source: BMC Zool. 2022 Sep 7;7:51. doi: 10.1186/s40850-022-00136-7 (PMC10127072; doi:10.1186/s40850-022-00136-7)
Supplement: Supplementary file 1 — Additional file 1. List of additional material examined. [file 40850_2022_136_MOESM1_ESM.docx]

# Additional file 1. List of additional material examined.

*Cleocnemis heteropoda*

Additional material examined. **BRAZIL**: **Minas Gerais**: Inconfidentes, Bairro Monjolinho, Fazenda Santa Luzia, 22.3336°S, 46.2974°W, 1♀, i-ii.2015, Souza, M. M. (UFMG 19728); **Paraná**: Ponta Grossa, Fazenda Paiquerê, 1♀, ii.2002, Nascimento, E. (IBSP 040980), São José dos Pinhais, 25.605°S, 49.1936°W, 18♂, 6♀, 02.i.2014-01.vii.2015, Domahovski, A.C. (MCTP 39023), 2♂, 9.xii.1986, Eq. Profaupar (MCTP 19445), 2♂, 10.xi.1986, Eq. Profaupar (MCTP 19443), 1♂, 3.xi.1986, Eq. Profaupar (MCTP 19447), 3♂, 5♀, 09.xii.2015, Domahovski, A.C. (MCTP 39087), 2♂, 24.xi.1986, Eq. Profaupar (MCTP 19446), Tijucas do Sul, Morro do Cabral, Lagoa, 1♂, 2♀, x.2000, Ricetti, J. (IBSP 039237); **Rio de Janeiro**: Angra dos Reis, Ilha Grande, 1♂, xi.2017, Queiroz, R., Silveira, L., Campello, L. (UFRJ 1641); Angra dos Reis, Ilha Grande, Pico do Papagaio, 3♂, i.2018, L.C., R.Q., L.S.,S.V. (UFRJ 1645), 1♂ i.2018, L.C., R.Q., L.S.,S.V. (UFRJ 1637), 4♂, xii.2017, Queiroz, R., Silveira, L., Campello, L. (UFRJ 1648); Rio de Janeiro, PN da Tijuca, 1♂, 04.x.2016, Eq. Lab. Entomologia UFRJ (UFRJ 1562), 1♂, 04.x.2016, Eq. Lab. Entomologia UFRJ (UFRJ 2000), Rio de Janeiro, PN da Tijuca, Archer, 2♀, 19.i.2005, Moreira, T. S. (MNRJ 06527), Rio de Janeiro, PN da Tijuca, Gávea, 2♀, 18.i.2005, Villareal M. O. (MNRJ 06526), 1♀, 18.i.2005, Villareal M. O. (MNRJ 06525), Teresópolis, PN da Serra dos Órgãos, P2, 1♂, 1♀, 29.xi.2013, Prado, A.W. (UFRJ 1630), 2♂, 04.i.2014, Equipe LEI (Lab. Ecol. Insetos) (UFRJ 1632), Teresópolis, PN da Serra dos Órgãos, P3, 3♂, 21.xi.2013, Equipe LEI (Lab. Ecol. Insetos) (UFRJ 1631), Teresópolis, PN da Serra dos Órgãos, Trilha Suspensa, 5♂, 6♀, 16.xii.2019, Oliveira, G.A. & Schinelli, H. B. (UFRJ 1650), 1♀, 16.xii.2019, Oliveira, G.A. & Schinelli, H. B. (UFRJ 1638), 1♀, 18.xii.2019, Schinelli, H. (UFRJ 1640), Teresópolis, PN Serra dos Órgãos, 2♂, 4.i.2014, Lab. Ecol. Insetos, UFRJ (UFRJ 1969), 1♂, 1♀, 29.xi.2013, Lab. Ecol. Insetos, UFRJ (UFRJ 1970); **Rio Grande do Sul**: Cambará do Sul, Itaimbezinho, 29.0478°S, 50.1447°W, 1♀, 6.i.1985, Lise, A. (MCTP 21419), São Francisco de Paula, , 29.4481°S, 50.5836°W, 1♂, 4♀, 09-12.i.1997, Lise, A. (MCTP 10743), São Francisco de Paula, Potreiro Velho, 29.4806°S, 50.175°W, 5♀, 9.x.1994, Braul, A. & Ott, R. (MCTP 05483), 3♀, 21-24.iii.1995, Lise, A. (MCTP 11990), 1♂, 3♀, 5-9.xii.1997, Lise, A. (MCTP 15972), 3♂, 13♀, 14-17.xii.1996, Lise, A. *et al.* (MCTP 10959), 2♂, 2♀, 12-15.xi.1998, Lise, A. *et al.* (MCTP 14386), 1♂, 1♀, 24.x.1996, Ott, R. (MCTP 10664), 3♂, 7♀, 5-8.xii.1996, Lise, A. *et al.* (MCTP 13898), 7♂, 2♀, xii.2001, Bertoncello, L. A. & Lise, A. (MCTP 19476), 1♂, 1♀, xii.2001, Bertoncello, L. A. & Lise, A. (MCTP 19477), São Francisco de Paula, Potreiro Velho, Pró-Mata, 29.4481°S, 50.5836°W, 1♂, 1♀, 24.x.1996, Ott, R. (MCTP 10665), 2♂, x.2001, Bertoncello, L. A. (MCTP 19463), São Francisco de Paula, Potreiro Velho, Pró-Mata, 29.4145°S, 50.2567°W, 1♂, 1♀, 24.x.1996, Ott, R. (MCTP 10665), 2♂, x.2001, Bertoncello, L. A. (MCTP 19463); **Santa Catarina**: Blumenau, Parque Natural Municipal Nascentes do Garcia, 27 01 S, 49 09 W, 27.0167°S, 49.15°W, 3♀, 21-28.i.2003, Eq. Biota Fapesp (IBSP 138316), Florianópolis, Costão do Santinho, Morro das Aranhas, 1♀, 2007, F. Albertoni (IBSP 144188); **São Paulo**: Mogi das Cruzes, PNM Serra do Itapety, 23 29 S, 46 12 W, 23.4833°S, 46.2°W, 1♂, 13-19.x.2003, Eq. Biota Fapesp (IBSP 138233), Peruíbe, EE Juréia-Itatins, Despraiado, 1♂, 30.ix.1997, Bertani, R. (IBSP 013540), Salesópolis, EE Boracéia, 23.5333°S, 45.85°W, 1♀, 18-24.v.2001, Eq. Biota Fapesp (IBSP 138241), 3♀, 26-28.i.1999, Pinto-da-Rocha, R., Casari, Ram (MZSP 17525), Salesópolis, EE Boracéia, 1♀, 18-24.v.2001, Eq. Biota Fapesp (IBSP 138241), 3♀, 26-28.i.1999, Pinto-da-Rocha, R., Casari, Ram (MZSP 17525), Santo André, Alto da Serra do Paranapiacaba, 2♂, 3♀, 14-16.xii.2003, Rheims, CA & Indicatti, R (IBSP 052082), São Luiz do Paraitinga, PE da Serra do Mar, Núcleo Santa Virgínia, 1♀, 21.i.2008, Maciel-Silva, A. S. (UFMG 06231).

*“Cleocnemis” lanceolata*

Additional material examined. **BRAZIL**: **Bahia**: Condeúba [ex Santo Antônio da Barra], 2♂, 1♀, [no date] (*Cleocnemis heteropoda*: Mello-Leitão det., MNHN 11501); **Mato Grosso**: Pontes e Lacerda, Usina Hidroelétrica de Guaporé, Vale São Domingos, 1♀, x.1999, Equipe Resgate (IBSP 041488B); **Mato Grosso do Sul**: Bonito, Anhumas , 21.1667°S, 56.5833°W, 1♀, 14-23.x.2002, Eq. BIOTA (IBSP 138362), Bonito, Baía Bonita, 21.15°S, 56.4333°W, 3♀, 14-23.x.2002, Eq. BIOTA (IBSP 138374); **Paraná**: Boa Vista da Aparecida, Flor da Serra, 1♂, 7-14.x.1996, Eq. IBSP (IBSP 21338A), Cascavel, Lago Municipal, 1♀, 13.i.2007, Silva, A. P. G. (IBSP 227801), Foz do Iguaçu, 25.5478°S, 54.5881°W, 1♂, 1♀, 16.xi.1991, Bonaldo, A.B. & Rodrigues, B.V.B (MCTP 01652); **Rio Grande do Sul**: Agudo, 1♂, 02.xi.2002, Morais, A.B.B. (MCTP 40437), Augusto Pestana, 1♀, 27.xii.2008, Silva, L. V. *et al.* (MCTP 26935), 1♀,Silva, L.V., Medeiros, L.B. (MCTP 30448), Cachoeira do Sul, Alto dos Casemiros, 2♀, 03.i.1994, Buss, R. G. (MCTP 04419), Cachoeira do Sul, Capanezinho, 30.0392°S, 52.8939°W, 2♀, 02.xi.1992, Buss, R. G. (MCTP 03324), Erval Grande, 1♀, i.1994, Braul, A. (MCTP 04490), Pântano Grande, 1♀, 05.iv.2008, Depra, G. (MCTP 40429), 2♀, 08.xii.2007, Depra, G. (MCTP 40430), Santa Cruz, 1♂, 06.iii.1994, Ott, R. leg. (MCTP 06571), Santa Maria, 2♂, 1♀, 16.xi.1998, Kotzian, C. B.; Indrusiak, L. (MCTP 39762), 3♀, 19.xi.1998, Kotzian, C.B., Indrusiak, L. (MCTP 40444), 1♂, 2♀, 16.xii.1997, Indrusiak, L., Monteiro, M. (MCTP 40434A), 1♀, 29.i.1998, Indrusiak, L., Monteiro, M. (MCTP 40432), 3♀, [no date], Kotzian, C. B.; Indrusiak, L. (MCTP 40431), Santa Maria, Cidade dos Meninos, 2♀, 22.i.1996, Ketzian, C.B., Indrusiak, L. (MCTP 40439), Santa Maria, Lar Metodista, 29.6842°S, 53.8069°W, 1♀, 15.xii.2004, Indrusiak, L. (MCTP 41414), Santa Maria, Perau Velho, 1♀, 19.01.1995, Ketzian, C.B., Indrusiak, L. (MCTP 40440), Santa Maria, São Marcos, 2♀, 10.i.1996, Ketzian, C.B., Indrusiak, L. (MCTP 40438), São Borja, Reserva Biológica São Donato, 28.6606°S, 56.0044°W, 1♀, 28.i.2012, Machado, M. (MCTP 34711), 3♀, 05.iii.2013, Machado, M. (MCTP 43390), São Borja, Reserva São Donato, 28.6606°S, 56.0044°W, 1♀, 11.x.2012, Machado, M. (MCTP 36951), Vicente Dutra, 1♀, 07.ii.2006, Trescher, T.F. (MCTP 22528); **Santa Catarina**: Chapecó, Quebra-Queixo, 1♀, 26-27.ii.2002 (MCTP 12892), São Cristóvão do Sul, Monte Alegre, 1♀, 2001-2002, Moreira, J. M. (IBSP 141470); **São Paulo**: Barueri, 1♀, 14.xii.1965, Linka, K. (MZUSP 13299), Itapetininga, 1♀, 11-16.xi.2002, Equipe BIOTA (IBSP 138275). **PARAGUAY**: **Alto Paraguay [ex Chaco]**: PN Defensores del Chaco, Cerro León: Sítio #24: Vale Pupukú, 1♀, 25.xi.1984, Kochalka, J.A. (IBNP 2580B); **Amambay**: Chacurrú, 1♀, 9-11.xi.2018, Piñanez, Y. (IBNP 50025); **Canindeyú**: Mbaracayú, 1♀, 6. xi.2018, Piñanez, Y. (IBNP 2960); **Central**: Asunción, Jardín Botánico, 1♂, 14.i.1994, Kochalka, J.A. (IBNP 2584); **Itapúa**: Capitán Miranda, Hotel El Tirol, 27.1833°S, 55.7778°W, 1♂, 1♀, 16-19.xi.2018, Piñanez, Y. (IBNP 50010); **Paraguari**: Paraguari, Cerro Hú, 1♀, 2.xi.2018, Piñanez, Y. (IBNP 2994), PN Ybycui, 1♀, 31.x-7.xi.1990, Herranz, J. (IBNP 2905); **San Pedro**: Santa Barbara, RN Laguna Blanca, 1♂, 1♀, 21.ix.2011, Recalde, F. (IBNP 2646).

*“Cleocnemis” mutilata*

Additional material examined. **BRAZIL**: **Espírito Santo**: Apiacá, Fazenda Santa Maria, 21.1538°S, 41.5664°W, 1♀, 23.xi.1996, Baptista, R. L. C. (MNRJ 06517); **Minas Gerais**: Belo Horizonte, 19.9167°S, 43.9345°W, 1♂, 01.vi.1993, Dutra, G. F. (IBSP 026815); 2♀, 13.vii.1993, Ferreira, A.H.P. & Santos, A.J. (IBSP 026816); **Rio de Janeiro**: Bom Jesus do Itabapoana, 21.1453°S, 41.6826°W, 1♀, 16.xi.1986, Baptista, R. L. C. (MNRJ 06493); 1♂, 29.viii.1987, Baptista, R. L. C. (MNRJ 06494); Casimiro de Abreu, Fazenda, 22.4798°S, 42.2029°W, 1♀, 04-13.viii.2010, Tinoco, D.C. (BRD 0797); Macaé, Colégio Matias Neto, 22.3731°S, 41.7813°W, 1♀, 30.xi.2010, Gabriela (UFRJ 0561); Macaé, Escola J. Calil Filho, 22.3288°S, 41.7396°W, 1♂, 7.vii.2010, Gabriela (UFRJ 0562), Rio de Janeiro, Ilha do Fundão, Catalão, 22.8445°S, 43.2213°W, 1♂, 5.x.2018, Prado, A. W. & Baptista R. L. C (UFRJ 1535); 3♂, 2♀, 11.vii.2019, Prado, A. W. (UFRJ 1643), Rio de Janeiro, Ilha do Fundão, Catalão, Forte, 22.8445°S, 43.2213°W, 3♂, 1♀, 31.x.2018, Prado, A. W. & Baptista R. L. C (UFRJ 1536), 1♂, 1♀, 10.xii.2018, Prado, A.W. & Schinelli, H.B. (UFRJ 1537), Rio de Janeiro, Ilha do Fundão, Encruzilhada Vila Residencial, 22.8646°S, 43.2196°W, 2♂, 20.ix.2019, Guimarães, C.A; Oliveira, F.S.M. (UFRJ 1642), Rio de Janeiro, Museu da República, 22.9259°S, 43.1762°W, 1♂, 15.ii.1996, Baptista, R. L. C. (MNRJ 06518); **São Paulo**: Mogi das Cruzes, São João, 23.5262°S, 46.1885°W, 1♀, 28.ix.2003, Lemos, R. Y. (IBSP 041835); Santos, 23.9475°S, 46.3367°W, 1♀, 16.ii.2005, Prefeitura Municipal de Santos (IBSP 051230); São Paulo, 23.5508°S, 46.6356°W, 1♀, 28.iii.1996, Ribeiro, M. H. (IBSP 014037), 1♂, 04.ix.2005, Machado, E. C. (IBSP 056258), 1♂, 06.iv.2005, Vieira, A. (IBSP 051337), 5♂, 10♀, [no date], Cunha, F. S. (IBSP 035782); São Paulo, 23.5505°S, 46.6333°W, 1♀, 28.iii.1996, Ribeiro, M. H. (IBSP 014037); 1♂, 04.ix.2005, Machado, E. C. (IBSP 056258); 1♂, 06.iv.2005, Vieira, A. (IBSP 051337); 5♂, 10♀, [no date], Cunha, F. S. (IBSP 035782); São Paulo, Instituto Butantan, campus, 23.5678°S, 46.7188°W, 1♂, ix.2007, Borreli, C. (IBSP 123975); São Paulo, Jardim Rizzo, URB, USP, 23.5614°S, 46.7308°W, 1♂, 1♀, 04.ii.1999, Candiani, D. F. (IBSP 028799); 1♀, 09.iii.1999, Castro, M. P. (IBSP 028873), 1♂, 2♀, 24.ii.1999, Cunha, F. S. (IBSP 028836); 1♀, 09.xi.1998, Brescovit, A.D. (IBSP 020677); 2♂, 4♀, 11.iii.1999, Japyassu, H. F. (IBSP 028966); 1♀, 01.iii.1999, Cunha, F. S. (IBSP 028889); São Paulo, Pinheiros, 23.5636°S, 46.6916°W, 1♀, 10.iii.2002, Cunha, F. S. (IBSP 033234); São Paulo, USP, Cidade Universitária, 23.5614°S, 46.7308°W, 1♂, 18.i.2000, Candiani, D. F. (IBSP 033351); 1♀, 12.v.2003, Cizauskas, i. (IBSP 131624); São Paulo, Vila Butantan, URB, USP, 23.5614°S, 46.7308°W, 1♀, 08.xii.200, Cunha, F. S. (IBSP 032968).

*Tibelloides bryantae*

Additional material examined. **BRAZIL**: **Bahia**: Iraquara, Pratinha, 12.3525°S, 41.5417°W, 1♂, 20.xii.1998, Rocha, L. S. (IBSP 020765), Senhor do Bonfim, UNEB, Campus VII, 3♂, 2♀, iii-vii.2008, Costa, J. S. (IBSP 133762); **Goiás**: Parque Nacional das Emas, Mineiros, 1♂, 18-22.iv.2000, Rheims, C. A. (IBSP 026289); **Mato Grosso**: Campo Verde, Fazenda Mourão, 1♂, 13.xi.2003, Silva, E. P. (IBSP 056227); **Minas Gerais:** Caeté, Gandarela, campos rupestres, 1♀, 22.i.2019, Prado, A. W. do (UFRJ 1542), Serra do Cipó, PN da Serra do Cipó, 1♀, v.2005, Gonzaga, M. O. (IBSP 059575), Serra do Cipó, PN da Serra do Cipó, Cânion, 1♀, 04.xi.2021, Prado, A. W. do, Schinelli, H. S., Baptista, R. L. C. (UFRJ 2001), Uberlândia, Clube da Ca, 1♀, 02.ix.2003, Della, G. (IBSP 043939B); **Pará**: Portel, Comunidade Santa Rosa, 1.85078°S, 50.6816°W, 1♂, 27.v.2016, Saturnino, R. (MPEG 035220), São Geraldo do Araguaia, Serra das Andorinhas, 6.23472°S, 48.4578°W, 1♂, 1♀, 27.x.2011, Bonaldo, A.B. et al. (MPEG 035224); **Pernambuco**: Buíque, PN do Catimbau, Terra 01, 1♂, 14.iii.2019, Takiya, D.M. (UFRJ 1561), Buíque, PN do Catimbau, terra 02, 1♀, 14.iii.2019, Takiya, D.M. (UFRJ 1560); **Piauí:** PN da Serra das Confusões, Terra 1, 1♂, 10.xi.2018, Prando, J.S. (UFRJ 2002); **Rio Grande do Norte**: Parnamirim, Mata do Jiqui, EMP ARN - Empresa de Pesquisa Agropecuária do RN, 6♂, 1♀, 24.ix.2008, Guedes, T. B. (IBSP 216559), 6♂, 17.ix.2008, Guedes, T. B. (IBSP 216909), Parnamirim, Mata do Jiqui, EMPARN-Empresa de Pesquisa Agropecuária do RN, 1♂, 17.ix.2008, Guedes, T.B. (IBSP 216697) **PARAGUAY**: **Amambay**: Chacurrú, 1♂, 2♀, 9.xi.2018, Piñanez, Y. (IBNP 50028); **Central**: Villeta, 2♀, 25.xi.2018, Piñanez, Y. (IBNP 2978); **Itapúa**: Alto Vera, Estación Biológica Kanguery: Campo natural, Estación n, 26.5167°S, 55.7833°W, 1♀, 12-16.i.2017, Piñanez, Y. (IBNP 2651), Nueva Arbolada, Museo del Árbol, 1♀, 19.xi.2018, Piñanez, Y. (IBNP 50070), San Miguel Potrero, entre Coronel Bogado y General Artigas, cauce del Arroyo Kambay, 1♀, 17.i.1996, Kochalka, J.A. (IBNP 2589); **Paraguari**: Paraguari, Hotel Gabriela, 1♂, 4.xi.2018, Piñanez, Y. (IBNP 2990). **VENEZUELA**: **Guarico**: Parque Nacional Aguero - Guariquito, 4♂, 4♀, 14.ix.1987, Candia, R. (MCTP 41290).

*Tibelloides punctulatus*

Additional material examined. ARGENTINA: **Misiones**: San Javier, 1♀, 11-21.iv.1989, Proj. Garabi (MCTP 00562). BRAZIL: **Acre**: Rio Branco, Embrapa, Campus, 10.0252°S, 67.6933°W, 1♂, xi.2012, Costa, L. M. S. (UFMG 12433), 3♂, xii.2012, Costa, L. M. S. (UFMG 14152); **Mato Grosso**: Canarana, Beira do Rio Kuluene, 1♀, [no date], Falatti, C.Q. & Ribeiro, A.K. (IBSP 027062), Vale de São Domingos/Pontes e Lacerda, UHE de Guaporé, 2♀, x.1999, Equipe Resgate (IBSP 041487B); **Mato Grosso do Sul**: Anaurilândia, 2♂, 2♀, 12-19.iii.2001 , Cunha, F.S. & Souza, C.A.R. (IBSP 039294), Anaurilândia, UHE Engenheiro Sérgio Motta, 22.3831°S, 52.8118°W, 1♂, 7♀, 15.xi-23.xii.1998, Equipe IBSP (IBSP 023424), Brasilândia, UHE Sérgio Motta, 1♀, 2000, Equipe IBSP (IBSP 035515), Corumbá, , 1♀, 1994, Raizer, J. (IBSP 006474), Corumbá, Passo do Lontra, abobral, 1♂, 1♀, 1997, Raizer, J. (IBSP 011445), Dois Irmãos do Buriti, APP Rio Cachoeirão, Fazenda Taruana, 1♂, 2♀, 16-26.ii.2008, Bessi, R. & Bervian, C. (IBSP 215238), Dois Irmãos do Buriti, Fazenda Taruana, 2♂, 3♀, 16-26.ii.2008, Bessi, R. & Bervian, C. (IBSP 221484), Dourados, 1♂, 1♀, 23.ii.1982 (MCTP 18950), 1♀, 23.iii.1982 (MCTP 18954), 1♂, 01.iii.1982 (MCTP 18953), 1♂, 23.ii.1983 (MCTP 18952), Nhecolândia, Fazenda do Leque, EMBRAPA, 3♀, 11.xi.1987, Harris, S. (MNRJ 02631), Passo do Lontra, Base de Estudos do Pantanal - UFMS, 2♀, 22.v.1993, Raizer, J. (IBSP 020513), Santa Rita do Rio Pardo, 1♀, 24.iv.2001, Bertani, R. & Kashimata, E.K. (IBSP 039528 A); **Minas Gerais**: Jaboticatubas, PN da Serra do Cipó, 1♀, 28.ii-8.iii.2002, Álvares, E. S. S. (IBSP 138345), Montes Claros, UFMG, 1♀, 2001, Leite, G.L.D. (IBSP 036922), Prudente de Morais, 1♂, viii.2005, Morais, P. (IBSP 099084), Prudente de Morais, Fazenda Sapé, 19.5°S, 44.1167°W, 1♀, 02.ix.2000, Álvares, E. S. S. (UFMG 00618), Unaí, UHE Queimado, 1♀, viii.2003, Machado, E. O. (IBSP 072578); **Pará**: Altamira, Novo Progresso, 7.15194°S, 55.3056°W, 1♀, 20.xi.2005, Santos-Souza, D. R. (MPEG 002720), Marabá, Mina do Sossego, Serra Norte, Carajás, 6.60278°S, 50.3286°W, 2♂, 3♀, 05-06.iii.2004, Marreco-Pedroso, A. (MPEG 003974), 10♂, 9♀, 23.ii-06.iii.2004, Marreco-Pedroso, A. (MPEG 004108), 1♂, 2♀, [no date], Marreco-Pedroso, A. (MPEG 003983), 9♂, 9♀, 03.iii.2004, Lobo, D. S. (MPEG 004099), 1♂, 05.iii.2004, Wanzeler, E. (MPEG 003986); **Paraná**: Boa Vista da Aparecida, Flor da Serra, 1♂, 1♀, 7-14.x.1998, Equipe IBSP (IBSP 021107), Capitão Leônidas Marques, Salto Caxias, Rio Iguaçu, 1♂, 5♀, 28.ii.1993, Bonaldo, A. (MCTP 04324), Céu Azul, 6♂, 4♀, 10.ii.2020, Prado, A.W. do (UFRJ 1971), Cruzeiro do Iguaçu, 1♂, 1♀, 8-15.x.1998, Eq. IBSP (IBSP 021388), Dois Vizinhos, UHE de Salto Caxias, Foz do Chopim, Cruzeiro do Iguaçu, 1♀, 8-15.x.1998, Equipe IBSP (IBSP 021047), Francisco Beltrão, 1♂, 1♀, 09.ii.2020, Prado, A.W. do & Baptista R.L.C. (UFRJ 1973), Pinhão/Candói, Usina Hidrelétrica Santa Clara, Rio Jordão, 25.6478°S, 51.9536°W, 1♀, 2005, Ricetti, J. et. al. (IBSP 143576); **Rio de Janeiro**: Rio de Janeiro, Campo dos Afonsos, 1♂, 13.xi.2008, Eq. Lab. Entomologia Forense UCB (UFRJ 0481), 1♀, 29.x.2008, Eq. Lab. Entomologia Forense UCB (UFRJ 0484), 1♂, 23.xi.2008, Eq. Lab. Entomologia Forense UCB (UFRJ 0483), 1♀, 30.iv.2009, Eq. Lab. Entomologia Forense UCB (UFRJ 0486), 1♀, 22.x.2008, Eq. Lab. Entomologia Forense UCB (UFRJ 0482), 1♂, 21.iv.2009, Eq. Lab. Entomologia Forense UCB (UFRJ 0485), Rio de Janeiro, Ilha do Fundão, Capim Limão, 1♀, 7.viii.2012, Luisa (UFRJ 0611), Rio de Janeiro, Pedra de Guaratiba, APA das Brisas, 1♂, 2♀, 13.xi.2018, Prado, A.W. do & Baptista R.L.C. (UFRJ 1539); **Rio Grande do Sul**: Vicente Dutra, 1♂, 07.ii.2020, Prado, A.W. do & Baptista R.L.C. (UFRJ 1974); **São Paulo**: Avanhandava, 1♂, 4♀, x.1982, Vizotto *et al.* (IBSP 042639), Onda Verde, Fazenda São João, 4♂, 1♀, i.1946, Lane, F. (MZSP 13297), Presidente Epitácio, UHE Sérgio Motta, 1♀, 16.i - 13.ii.1999, Equipe IBSP (IBSP 023118), Presidente Epitácio, UHE Sérgio Motta, Ilha da Lagoa do Machado, 1♀, 30.iii.2001, Indicatti, R.P. & Souza, C.A.R. (IBSP 038507), Primavera, Porto Primavera, 1♀, 23.ii.2001, Candiani, D. F. & Indicatti, R. P. (IBSP 039339B), Primavera, UHE Sérgio Motta, 11♂, 33♀, i-ii.2000, Equipe BIOTA (IBSP 029860A), 10♂, 18♀, i-ii/2000, Equipe IBSP (IBSP 029897), Rosana, UHE Rosana, 2♂, 2♀, xii.1986, Equipe IBSP (IBSP 004463), São Paulo, 1♂, 1♀, [no date], Cunha, F.S. (IBSP 035777B), São Paulo, Jardim Rizzo URBUSP, 1♀, 24.ii.1999, Cunha, F.S. (IBSP 028834), Três Irmãos, Três Irmãos, 1♂, x.1990, Costa & Bertim (IBSP 004878); **Tocantins**: Gurupi, 11.7458°S, 49.0525°W, 2♂, 29.vi.2015, Tschoeke, O.H. (UFMG 19056), Miracema, UHE Luis Eduardo Magalhães, 1♂, 11-21.x.2001, Bertani, R. & Toledo, W.I. (IBSP 031499), Palmas, Jardim Taquari, Taquaralto, 1♀, 4-10.xi.2002, Knysak, I. & Martins, R. (IBSP 219564), Palmas, Rio Tocantins, margem direita, 1♀, i.2002, Candido, D.M. & Costa, M. (IBSP 040431). PARAGUAY: **Alto Paraguay**: PN Defensores del Chaco, Madrejón, 1♀, 8.xii.1981, Kochalka, J.A. (IBNP 2578), 1♀, 16.xii.1981, Kochalka, J.A. (IBNP 2579); **Amambay**: Chacurrú, , 1♂, 1♀, 9-12.xi.2018, Piñanez, Y. (IBNP 50024); **Central**: San Lorenzo, Infona, 1♂, 17.iii.2016, Piñanez, Y. (IBNP 2595), Villeta, 1♂, 4♀, 02.ii.2020, Prado, A.W. & Baptista, R.L.C. (UFRJ 1976); **Cordillera**: San Bernardino, 1♂, 7-8.xi.1987, Kochalka, J.A. (IBNP 2587), 9♂, 9♀, 31.i.2020, Prado, A.W. do & Baptista, R.L.C. (UFRJ 1972); **Guairá**: Colonia Independencia, Hotel Tilinski, 2♂, 2♀, 14.xi.2018, Piñanez, Yolanda (IBNP 2987); **Itapúa**: Carmen del Paraná, donde el Arroyo Tacuarí cruce la Ruta 1, 1♀, 17.i.1996, Kochalka, J.A. (IBNP 2588), Nueva Arbolada, Museo del Árbol, 1♀, 19.xi.2018, Piñanez, Y. (IBNP 2969); **Paraguari**: Cerro Hú, Hotel Gabriela, 1♂, 1♀, 01.ii.2020, Prado, A.W. do & Baptista R.L.C. (UFRJ 1975).
