# Supplementary material for: Solving a running crab spider puzzle: delimiting Cleocnemis Simon, 1886 with implications on the phylogeny and terminology of genital structures of Philodromidae
Source: BMC Zool. 2022 Sep 7;7:51. doi: 10.1186/s40850-022-00136-7 (PMC10127072; doi:10.1186/s40850-022-00136-7)
Supplement: Supplementary file 2 — Additional file 2. Primers used for amplification and sequencing of molecular markers used in the phylogeny of Philodromidae. F – Forward, R – Reverse. [file 40850_2022_136_MOESM2_ESM.docx]

**Additional file 2**. Primers used for amplification and sequencing of molecular markers used in the phylogeny of Philodromidae. F – Forward, R – Reverse.

| **Primer** | **Direction** | **Sequence (5’-3’)** | **Reference** |
| --- | --- | --- | --- |
| LCO-1490 | F | GGTCAACAAATCATAAAGATATTGG | Folmer *et al.* 1994 |
| C1-J-1718 | F | GGAGGATTTGGAAATTGATTAGTTCC | Simon *et al.* 1994 |
| HCO-2198 | R | TAAACTTCAGGGTGACCAAAAAATCA | Folmer *et al.* 1994 |
| HexAF | F | ATGGCTCGTACCAAGCAGACGGC | Ogden & Whiting 2003 |
| HexAR | R | ATATCCTTGGGCATGATGGTGAC | Ogden & Whiting 2003 |
| LR-J-12887 | F | CCGGTYTGAACTCARATCA | Takiya *et al.* 2006 |
| LR-N-13398 | R | CRMCTGTTTAWCAAAAACAT | Takiya *et al.* 2006 |
| 28S-A | F | GACCCGTCTTGAAGCACG | Wheeler *et al.* 2017 |
| 28S-Bout | R | CCCACAGCGCCAGTTCTGCTTACC | Wheeler *et al.* 2017 |
| 28S-Rd4.8a | F | ACCTATTCTCAAACTTTAAATGG | Wheeler *et al.* 2017 |
| 28S-Rd7b1 | R | GACTTCCCTTACCTACAT | Wheeler *et al.* 2017 |

**References:**

**Folmer O, Black M, Hoeh W, Lutz R, Vrijenhoek R.** **1994.** DNA primers for amplification of mitochondrial cytochrome c oxidase subunit I from diverse metazoan invertebrates, *Molecular Marine Biology and Biotechnology* 3: 294–299.

**Ogden TH & Whiting M.** **2003.** The problem with “the Paleoptera problem”: sense and sensitivity, *Cladistics* 19: 432–442.

**Simon C, Frati F, Beckenbach A, Crespi B, Liu H, Flook P.** **1994.** Evolution, weighting, and phylogenetic utility of mitochondrial gene sequences and a compilation of conserved polymerase chain reaction primers, *Annals of the Entomological Society of. America* 87: 651–701.

**Takiya, DM, Tran PL, Dietrich CH, & Moran NA.** **2006.** Co‐cladogenesis spanning three phyla: leafhoppers (Insecta: Hemiptera: Cicadellidae) and their dual bacterial symbionts, *Molecular ecology* 15(13): 4175–4191.

**Wheeler WC, Coddington JA, Crowley, LM, Dimitrov, D, Goloboff, PA, Griswold, CE, Hormiga, G., Prendini L, Ramírez MJ, Sierwald P, Almeida-Silva L, Alvarez-Padilla F, Arnedo MA, Silva LRB, Benjamin SP, Bond JE, Grismado CJ, Hasan E, Hedin M, Izquierdo MA, Labarque FM, Ledford J, Lopardo L, Maddison WP, Miller JA, Piacentini LN, Platnick NI, Polotow D, Silva-Dávila D, Scharff N, Szűts N, Ubick D, Vink CJ, Wood HM, Zhang J. 2017.** The spider tree of life: phylogeny of Araneae based on target-gene analyses from an extensive taxon sampling, *Cladistics* 33(6): 576–616.
