# Supplementary material for: Solving a running crab spider puzzle: delimiting Cleocnemis Simon, 1886 with implications on the phylogeny and terminology of genital structures of Philodromidae
Source: BMC Zool. 2022 Sep 7;7:51. doi: 10.1186/s40850-022-00136-7 (PMC10127072; doi:10.1186/s40850-022-00136-7)
Supplement: Supplementary file 3 — Additional file 3. Partition scheme and respective model suggested by BIC in ModelFinder for the concatenated molecular dataset of Philodromidae. [file 40850_2022_136_MOESM3_ESM.docx]

**Additional file 3**: Partition scheme and respective model suggested by BIC in ModelFinder for the concatenated molecular dataset of Philodromidae.

|  | **Partitions** | **MrBayes models** | **IQ-TREE models** |
| --- | --- | --- | --- |
| **1** | COI_pos1 | GTR+F+G4 | TIM2+F+G4 |
| **2** | COI_pos2 | HKY+F+I | TN+F+I |
| **3** | COI_pos3 | HKY+F+G4 | HKY+F+R2 |
| **4** | H3_pos1 + 28S | GTR+F+I+G4 | TIM+F+R2 |
| **5** | H3_pos2 | JC+I | JC+I |
| **6** | H3_pos3 | SYM+G4 | TIM2e+G4 |
| **7** | 16S | GTR+F+I+G4 | TIM2+F+I+G4 |
