# Supplementary material for: Solving a running crab spider puzzle: delimiting Cleocnemis Simon, 1886 with implications on the phylogeny and terminology of genital structures of Philodromidae
Source: BMC Zool. 2022 Sep 7;7:51. doi: 10.1186/s40850-022-00136-7 (PMC10127072; doi:10.1186/s40850-022-00136-7)
Supplement: Supplementary file 4 — Additional file 4. Resulting phylogenetic trees of Philodromidae: (a) ML of concatenated matrix with clade supports of SH-aLRT/UFBoot, and BI analyses of (b) concatenated matrix, (c) COI, (d) H3, (e) 16S, and (f) 28S with posterior probabilities values. [file 40850_2022_136_MOESM4_ESM.pdf]

Additional file 4. Resulting phylogenetic trees of Philodromidae: (a) ML of concatenated matrix with clade supports of SH-aLRT/UFBoot, and BI analyses of (b) concatenated matrix, (c) COI, (d) H3, (e) 16S, and (f) 28S with posterior probabilities values.

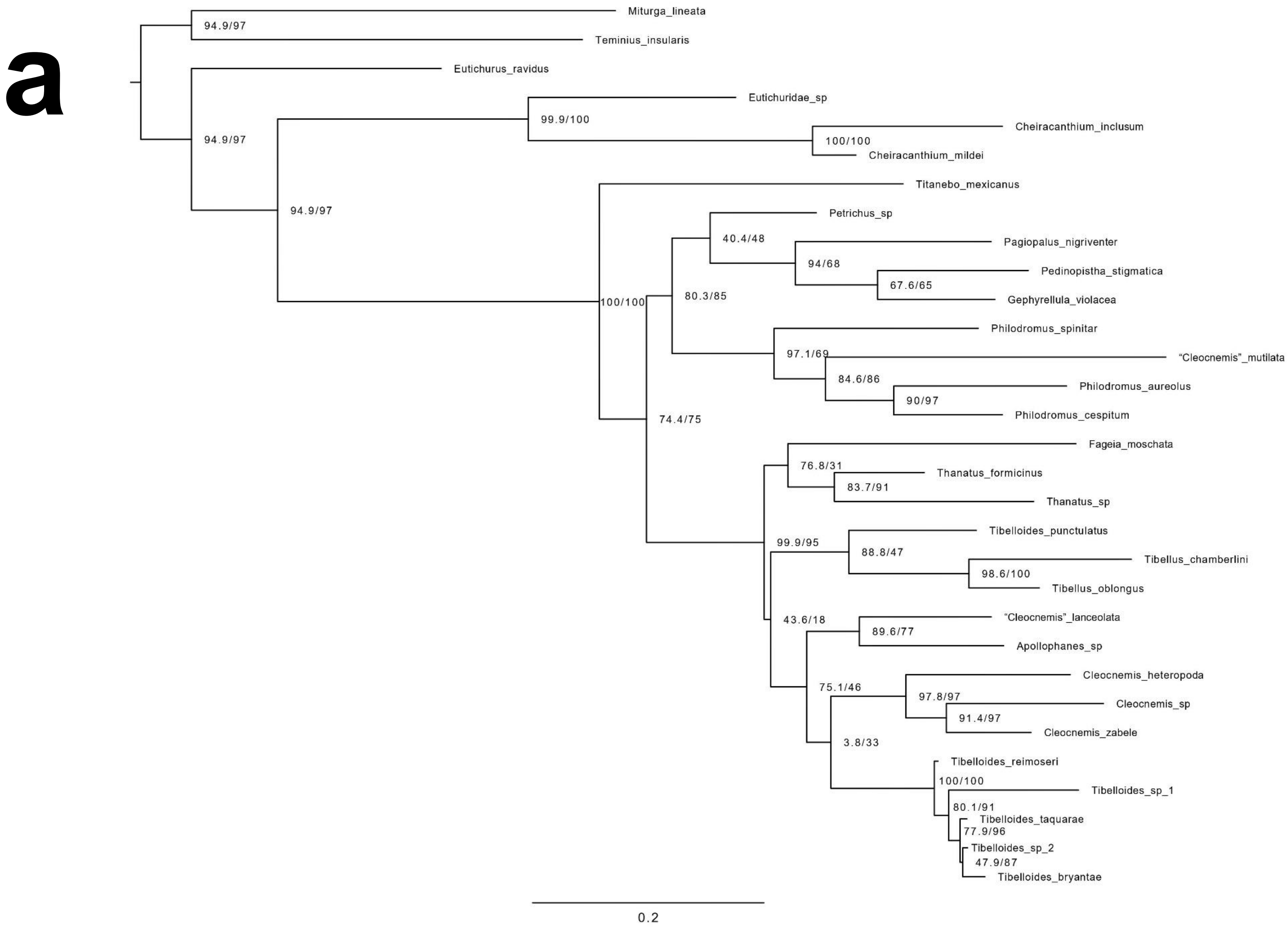

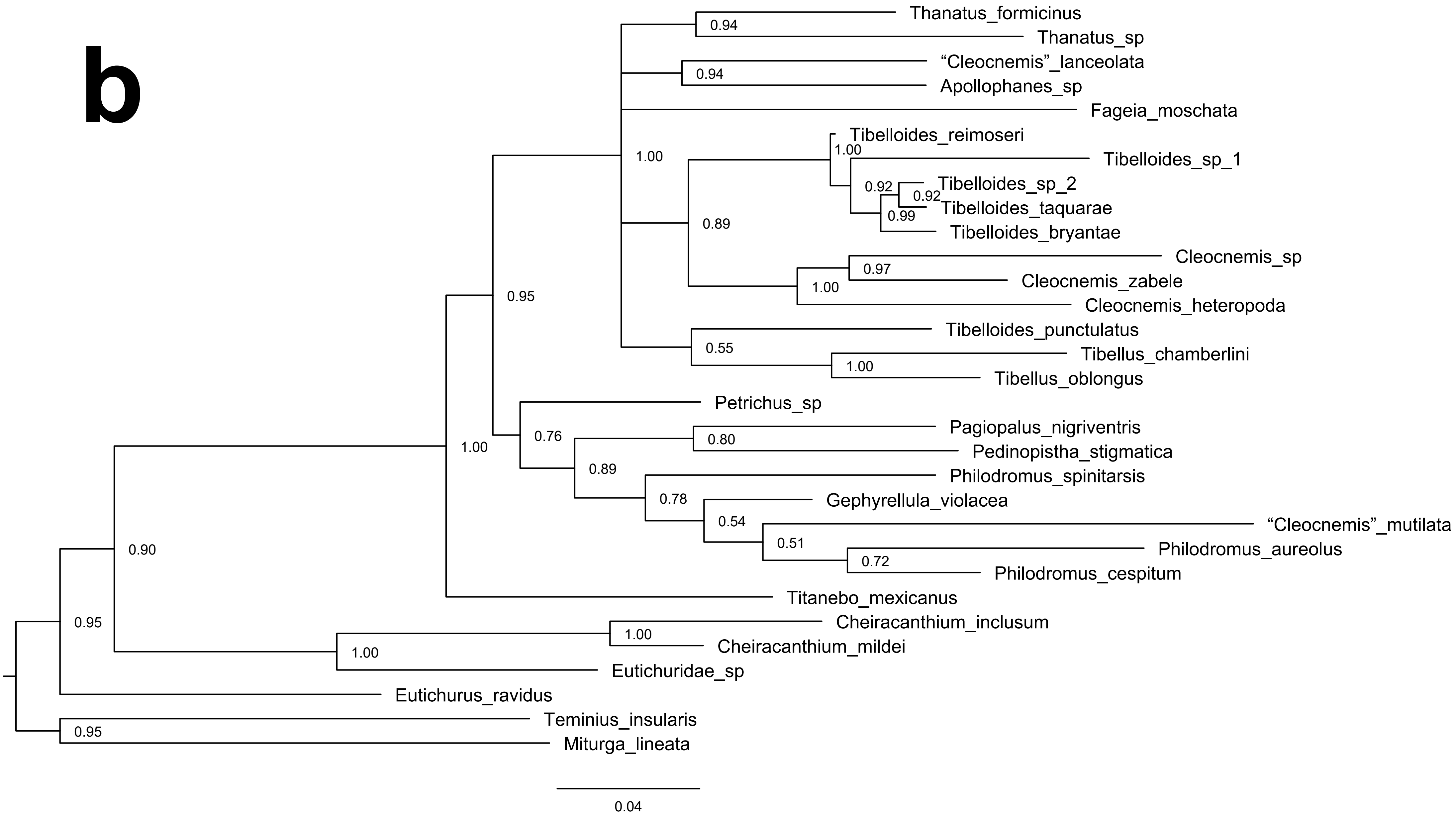
b
Thanatus_formicinus
0.94
Thanatus_sp
“Cleocnemis”_lanceolata
0.94
Apollophanes_sp
Fageia_moschata
Tibelloides_reimoseri
1.00
1.00
Tibelloides_sp_1
0.92
Tibelloides_sp_2
0.92
Tibelloides_taquarae
0.99
Tibelloides_bryantae
0.89
Cleocnemis_sp
0.97
Cleocnemis_zabele
1.00
Cleocnemis_heteropoda
0.95
Tibelloides_punctulatus
0.55
Tibellus_chamberlini
1.00
Tibellus_oblongus
Petrichus_sp
Pagiopalus_nigriventris
0.76
0.80
Pedinopistha_stigmatica
1.00
0.89
Philodromus_spinitarsis
0.78
Gephyrellula_violacea
0.54
“Cleocnemis”_mutilata
0.90
0.51
Philodromus_aureolus
0.72
Philodromus_cespitum
Titanebo_mexicanus
0.95
Cheiracanthium_inclusum
1.00
Cheiracanthium_mildei
1.00
Eutichuridae_sp
Eutichurus_ravidus
Teminius_insularis
0.95
Miturga_lineata
0.04

C

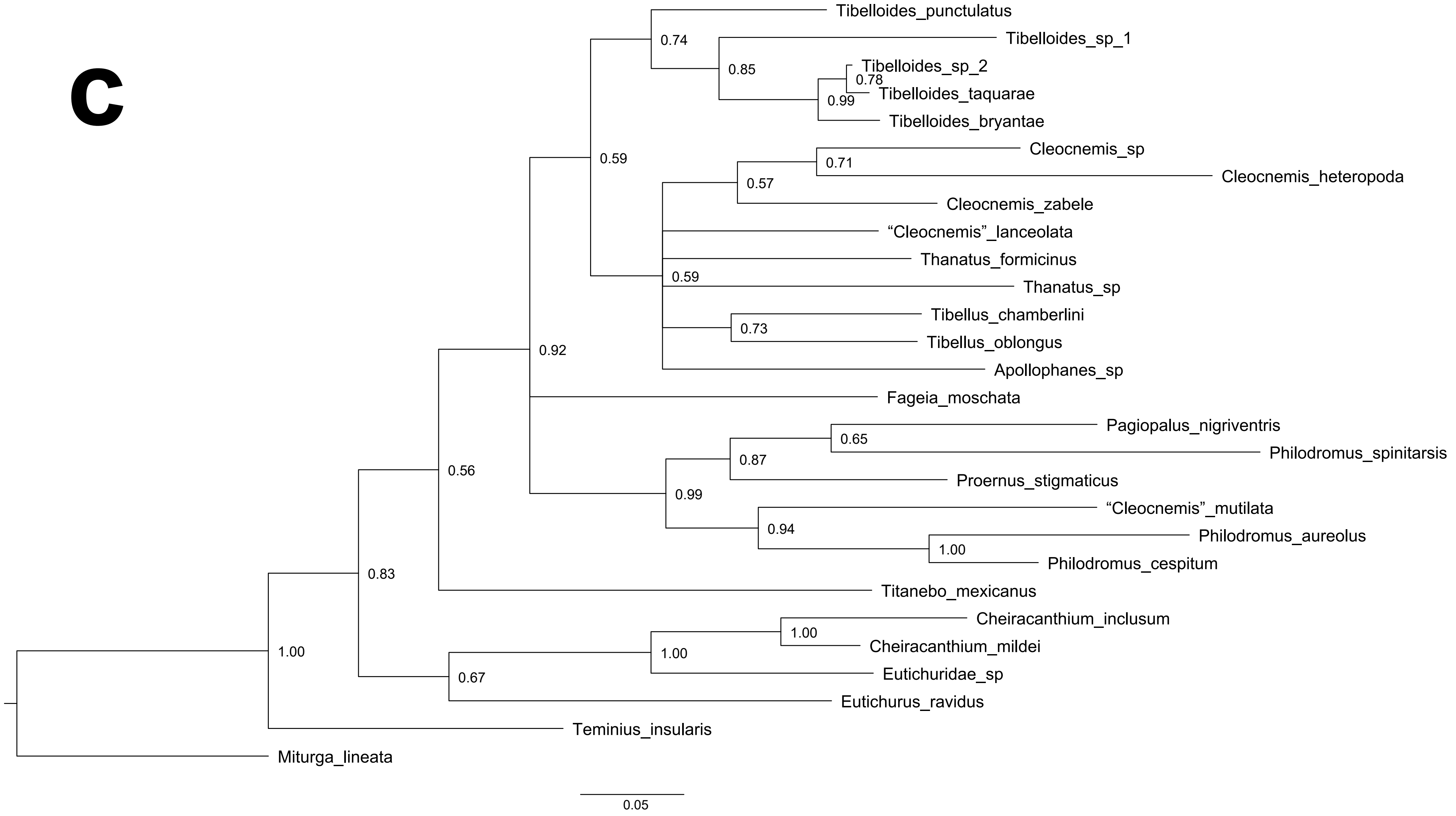
Tibelloides_punctulatus
Tibelloides_sp_1
Tibelloides_sp_2
Tibelloides_taquarae
Tibelloides_bryantae
Cleocnemis_sp
Cleocnemis_heteropoda
Cleocnemis_zabele
“Cleocnemis”_lanceolata
Thanatus_formicinus
Thanatus_sp
Tibellus_chamberlini
Tibellus_oblongus
Apollophanes_sp
Fageia_moschata
Pagiopalus_nigriventris
Philodromus_spinitarsis
Proernus_stigmaticus
“Cleocnemis”_mutilata
Philodromus_aureolus
Philodromus_cespitum
Titanebo_mexicanus
Cheiracanthium_inclusum
Cheiracanthium_mildei
Eutichuridae_sp
Eutichurus_ravidus
Teminius_insularis
Miturga_lineata
0.74
0.85
0.78
0.99
0.59
0.71
0.57
0.59
0.73
0.92
0.65
0.87
0.56
0.99
0.94
1.00
0.83
1.00
1.00
1.00
0.67
0.05

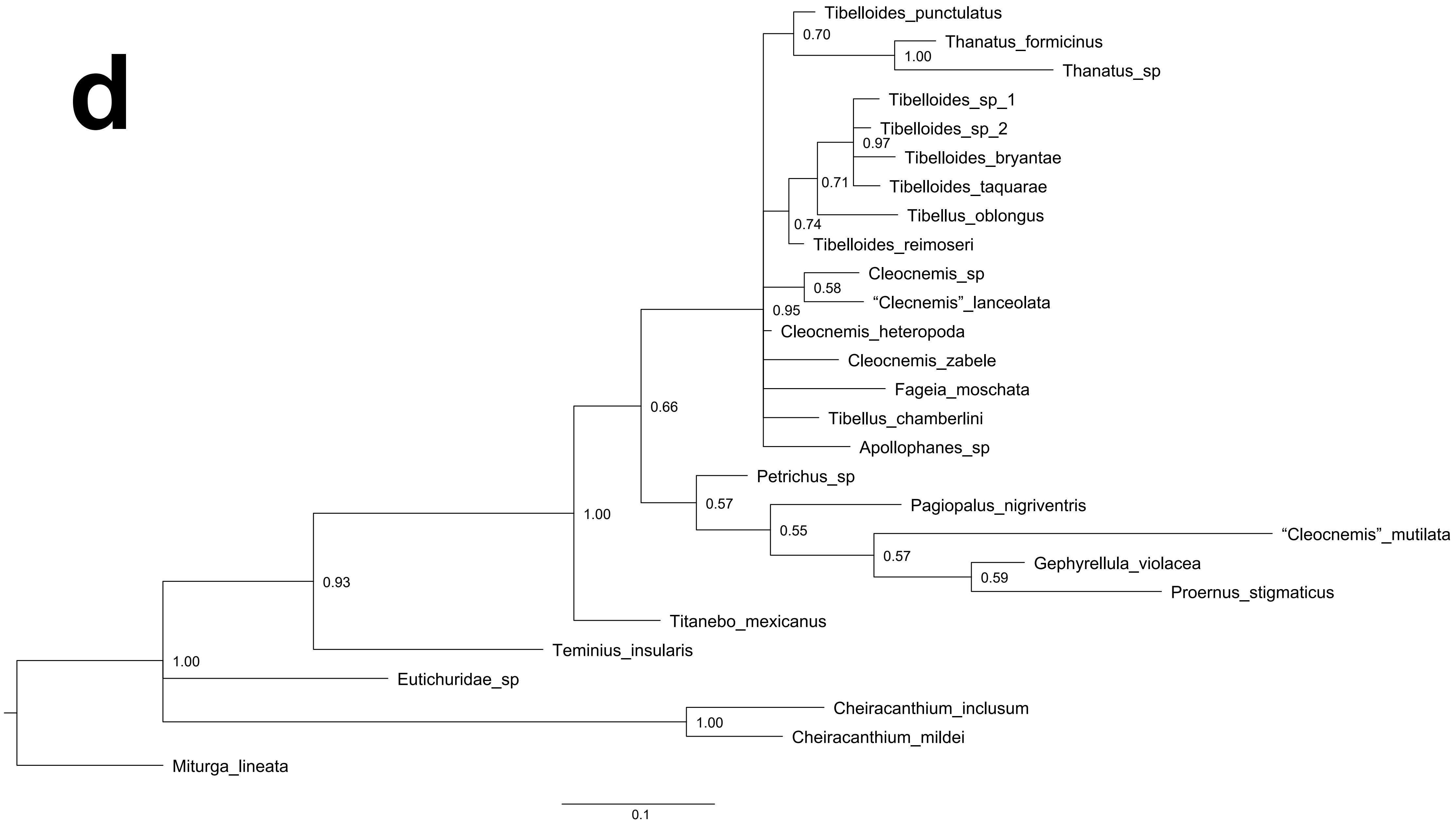
d
Tibelloides_punctulatus
0.70
Thanatus_formicinus
1.00
Thanatus_sp
Tibelloides_sp_1
Tibelloides_sp_2
0.97
Tibelloides_bryantae
0.71
Tibelloides_taquarae
Tibellus_oblongus
0.74
Tibelloides_reimoseri
Cleocnemis_sp
0.58
"Clecnemis"_lanceolata
0.95
Cleocnemis_heteropoda
Cleocnemis_zabele
Fageia_moschata
0.66
Tibellus_chamberlini
Apollophanes_sp
Petrichus_sp
0.57
Pagiopalus_nigriventris
1.00
0.55
"Cleocnemis"_mutilata
0.57
Gephyrellula_violacea
0.93
0.59
Proernus_stigmaticus
Titanebo_mexicanus
Teminius_insularis
1.00
Eutichuridae_sp
Cheiracanthium_inclusum
1.00
Cheiracanthium_mildei
Miturga_lineata
0.1

e

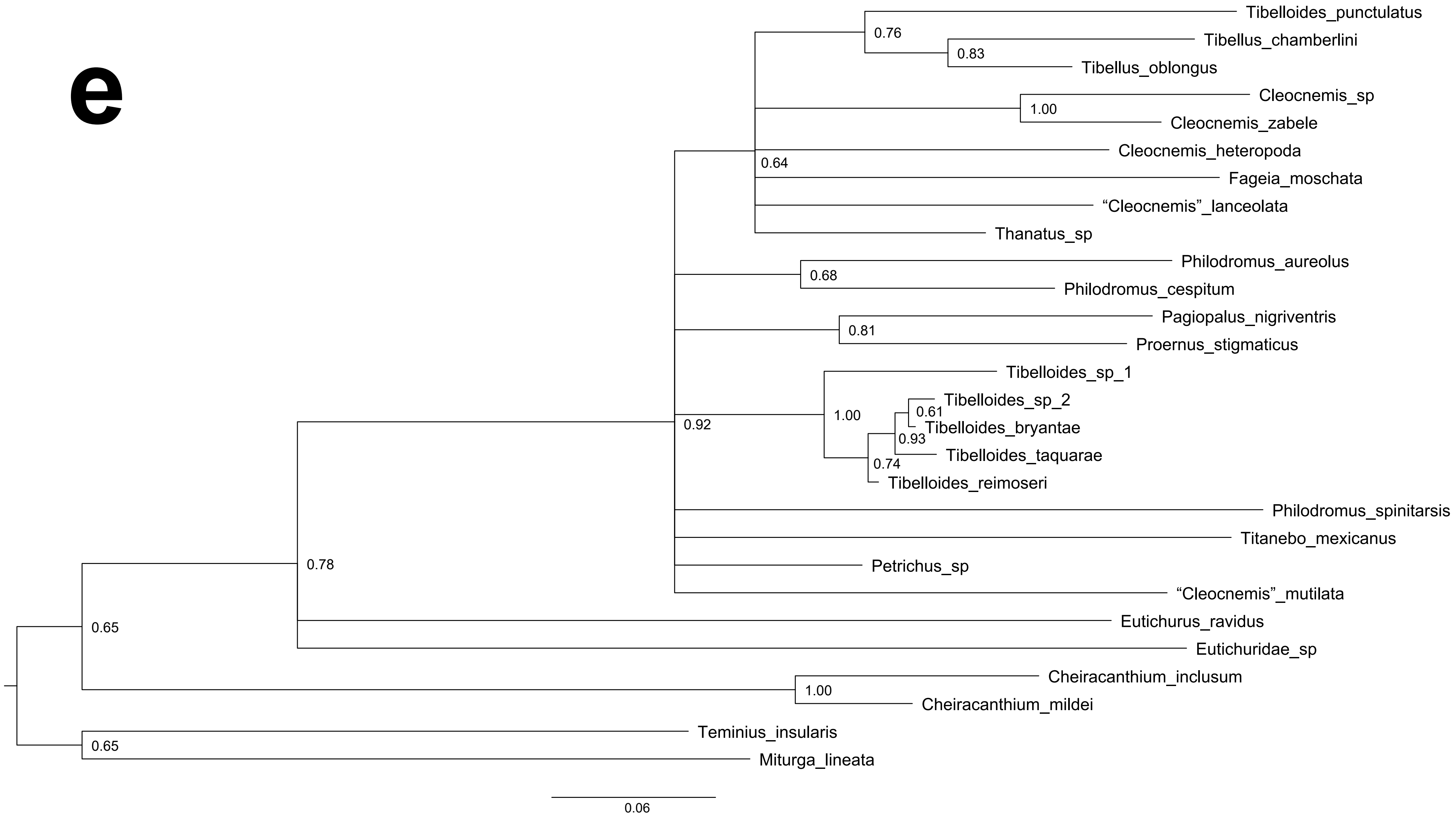

Tibelloides_punctulatus
0.76
Tibellus_chamberlini
0.83
Tibellus_oblongus
Cleocnemis_sp
1.00
Cleocnemis_zabele
Cleocnemis_heteropoda
0.64
Fageia_moschata
"Cleocnemis"_lanceolata
Thanatus_sp
Philodromus_aureolus
0.68
Philodromus_cespitum
Pagiopalus_nigriventris
0.81
Proernus_stigmaticus
Tibelloides_sp_1
Tibelloides_sp_2
1.00
0.61
0.92
Tibelloides_bryantae
0.93
Tibelloides_taquarae
0.74
Tibelloides_reimoseri
Philodromus_spinitarsis
Titanebo_mexicanus
0.78
Petrichus_sp
"Cleocnemis"_mutilata
Eutichurus_ravidus
0.65
Eutichuridae_sp
Cheiracanthium_inclusum
1.00
Cheiracanthium_mildei
Teminius_insularis
0.65
Miturga_lineata
0.06

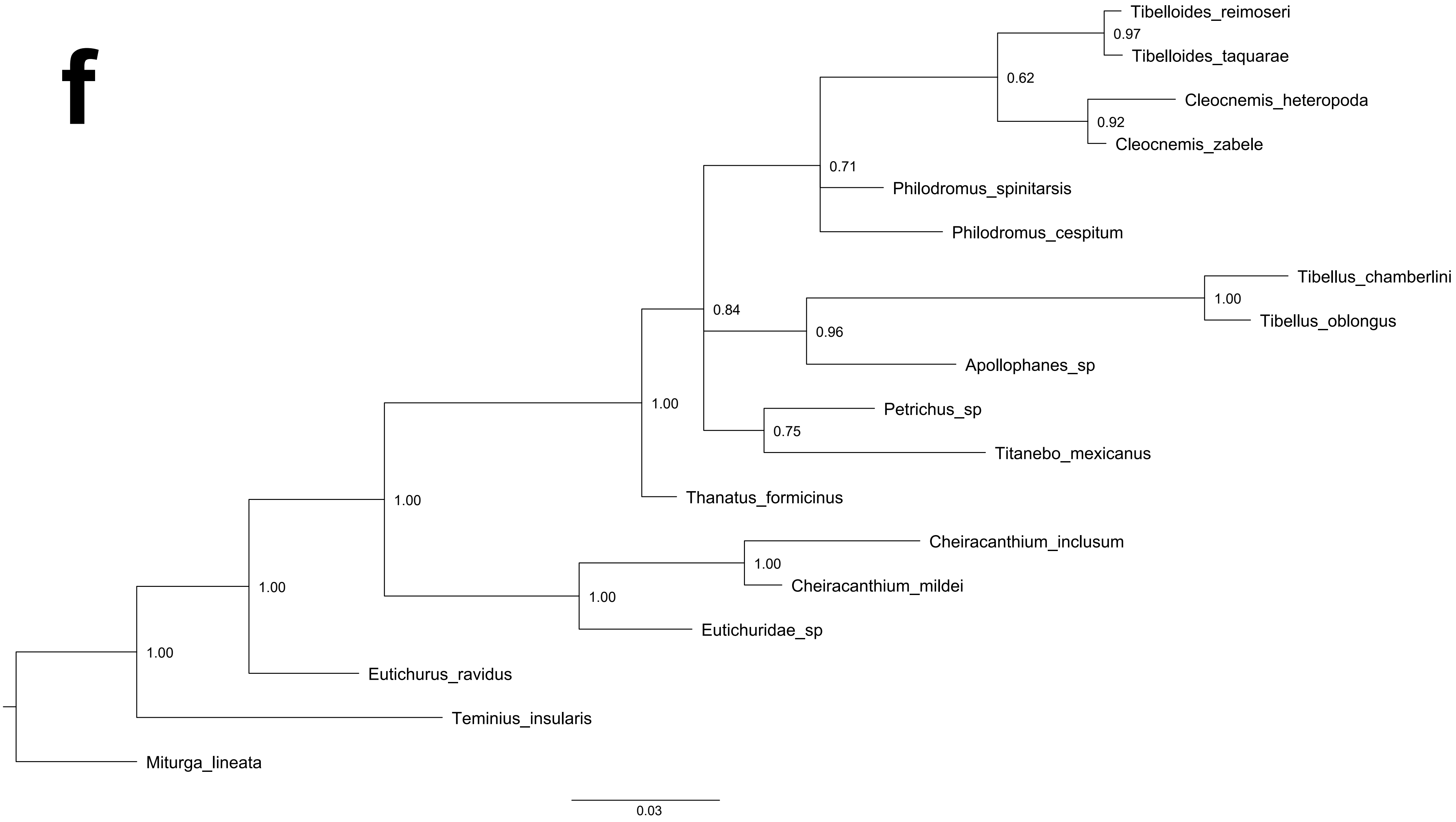
f
Tibelloides_reimoseri
0.97
Tibelloides_taquarae
0.62
Cleocnemis_heteropoda
0.92
Cleocnemis_zabele
0.71
Philodromus_spinitarsis
Philodromus_cespitum
Tibellus_chamberlini
1.00
Tibellus_oblongus
0.84
0.96
Apollophanes_sp
1.00
Petrichus_sp
0.75
Titanebo_mexicanus
1.00
Thanatus_formicinus
Cheiracanthium_inclusum
1.00
Cheiracanthium_mildei
1.00
1.00
Eutichuridae_sp
1.00
Eutichurus_ravidus
Teminius_insularis
Miturga_lineata
0.03
